# GeneInsight: Condensing gene set knowledge via language models

**DOI:** 10.1371/journal.pcbi.1014500

**Published:** 2026-08-05

**Authors:** Wee Loong Chin, Kevin Chen, Timo Lassmann

**Affiliations:** 1 National Centre for Asbestos-Related Diseases, University of Western Australia, Perth, Western Australia, Australia; 2 Department of Medical Oncology, Sir Charles Gairdner Hospital, Perth, Western Australia, Australia; 3 The Kids Research Institute Australia, University of Western Australia, Nedlands, Western Australia, Australia; Burnet Institute, AUSTRALIA

## Abstract

Gene set analysis often returns extensive annotations from multiple sources, requiring manual effort to identify coherent biological themes. We developed GeneInsight, an AI-powered tool that automates this by retrieving functional annotations from STRING-DB, clustering semantically related terms using sentence embeddings, and generating thematic summaries via large language model prompting. This enables researchers to identify biological themes that may be obscured when annotation sources are examined separately.

## Introduction

Gene set interpretation is a fundamental task in functional genomics, where researchers must derive biological insights from lists of genes identified in high-throughput experiments. Current approaches utilise statistical enrichment methods that query predefined functional databases, such as Gene Ontology [1] and KEGG pathways [2], to identify overrepresented biological processes [3,4]. Although powerful, current methods yield fragmented outputs, such as lists of enriched terms from various ontologies, leaving researchers to manually integrate these results to achieve functional insights, a process that is both inefficient and error-prone.

Exploratory gene set analysis has become increasingly challenging as the volume of annotated datasets grows. Researchers compare their findings not only with gene ontology term enrichments but also with signatures from knockdown experiments and diverse resources such as LINCS (Library of Integrated Network-based Cellular Signatures) [5] and the STRING database (STRING-DB) [6]. The main issue is that enrichment analysis tests for the over-representation of genes associated with specific terms. When gene lists overlap, the same genes often appear under many different functional terms. As a result, enrichment analysis can return many distinct-sounding terms that all point to the same underlying biology, making interpretation more difficult.

However, the challenge extends beyond simply removing duplicate information. Simplistic filtering of overlapping gene sets would obscure important biological relationships that only become apparent when analysing genes across the diverse resources mentioned above. These relationships often represent biological processes that bridge multiple databases and reveal insights not captured by any single resource. Consequently, manually curating enrichment outputs to identify both redundancies and meaningful biological patterns is not only error-prone and prohibitively time-consuming but also risks overlooking crucial biological connections.

We hypothesise that recently introduced topic modelling techniques can address this problem. By analysing how terms co-occur across texts, topic modelling reveals underlying themes without requiring predefined categories. These unsupervised statistical methods include Latent Dirichlet Allocation (LDA) [7] and Non-negative Matrix Factorization (NMF) [8], which identify recurring patterns in document collections. The resulting latent themes represent collections of related terms that frequently appear together and may correspond to biological processes, pathways, or functional modules not explicitly defined in current annotation databases.

Recent advances in large language models [9] (LLMs) create powerful new opportunities for gene set interpretation. LLMs have demonstrated remarkable capabilities in contextual understanding and natural language generation, potentially enabling automated synthesis of distributed biological knowledge. Several studies have explored this direction. Hu et al. [10] evaluated LLMs as “compressed databases” of biological literature, querying internal model weights to infer gene set function with external search used for post-hoc verification. GeneAgent [11] generates functional hypotheses from internal knowledge before fact-checking extracted claims against databases. llm2geneset [12] uses internal knowledge to dynamically construct gene set categories, with statistical testing applied afterwards.

Here we present GeneInsight, a tool that integrates LLMs with topic modelling to automate gene set interpretation. Our tool aggregates gene-specific annotations from the STRING database, applies topic modelling to identify coherent biological themes, and employs LLM-based summarisation to generate contextual interpretations of these themes. While existing LLM-based tools query the model’s internal knowledge to infer gene function, GeneInsight takes a different approach. We retrieve annotations directly from STRING and combine topic modelling with LLM summarisation to distil these into coherent biological themes, grounding interpretations in expert-curated database content.

## Design and implementation

**Fig 1:**
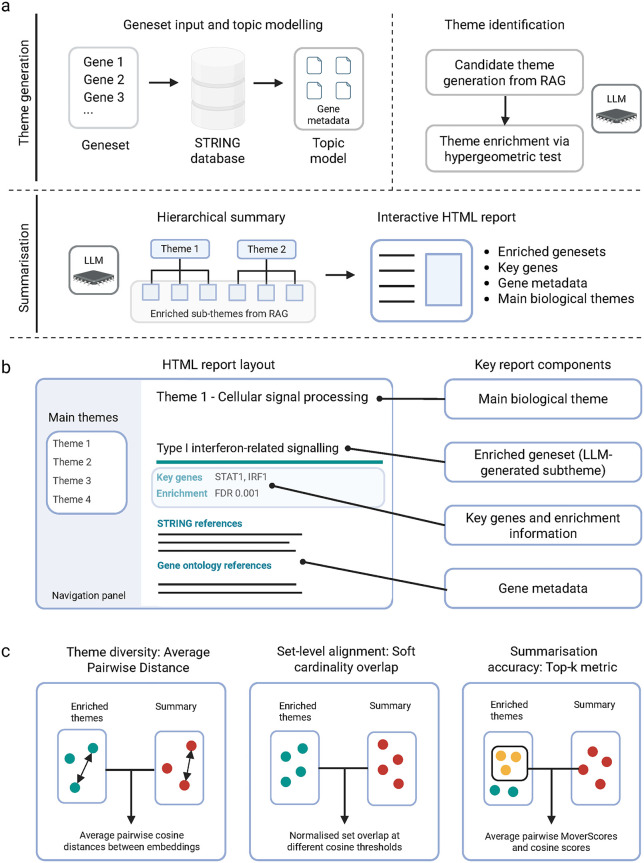
GeneInsight system architecture and workflow. **(a)** Schematic overview of the GeneInsight framework. The pipeline processes gene sets through two sequential stages: (1) Theme generation and (2) summarisation generates comprehensive reports with interactive visualisations. **(b)** Web interface components with components referenced in **(a)**. **(c)** Performance metrics used in benchmarking showing theme diversity using average pairwise distance, set-level alignment using soft cardinality overlap and summarisation accuracy using top-k metric. RAG, Retrieval augmented generation; LLM, Large language model; HTML, Hypertext markup language. Created in BioRender. Chin, W. (2026) https://BioRender.com/u37x4ls.

GeneInsight uses a two-stage approach to extract and organise biological information from gene sets (Fig 1a). In the biological theme generation stage, the system collects functional annotations from the STRING database for each input gene, creating a collection of gene-specific descriptions. This textual corpus is subjected to cluster-based topic modelling, which groups similar annotations into clusters (topics) and identifies key terms for each cluster. An LLM then converts representative annotations from each cluster into interpretable biological themes. Themes are then prioritised using an overlap-ratio threshold combined with an empirical p-value calibrated against a size-stratified random-query null (S1 File, Materials and Methods).

The second summarisation stage begins with another round of cluster-based topic modelling to identify key themes. This approach refines these enriched themes by measuring how consistently they appear as cluster representatives across multiple runs of topic modelling. The software then extracts the final summary by selecting themes to include based on user-defined length preferences. A large language model creates a hierarchical summary where major biological themes appear as main headings with related subheadings grouped beneath them. The final interactive HTML report (Fig 1b) links theme descriptions to their corresponding gene annotations. This integration enables researchers to easily navigate between overarching biological processes and their specific components. Moreover, every theme is directly tied to the original STRING-derived annotation outputs, allowing users to trace each summary theme back to its supporting gene-level descriptions and source annotations.

## Results

We first characterised GeneInsight’s theme-prioritisation stage by comparing its retained themes with the STRING database functional enrichment application programming interface (API). Using identical underlying gene-level information and 1,000 Molecular Signatures Database (MSigDB) [14] gene sets, this comparison directly assessed our tool’s effectiveness in identifying important biological themes. We measured performance through metrics (Fig 1c) that evaluated both the diversity of identified concepts and the degree of overlap between methods.

**Fig 2:**
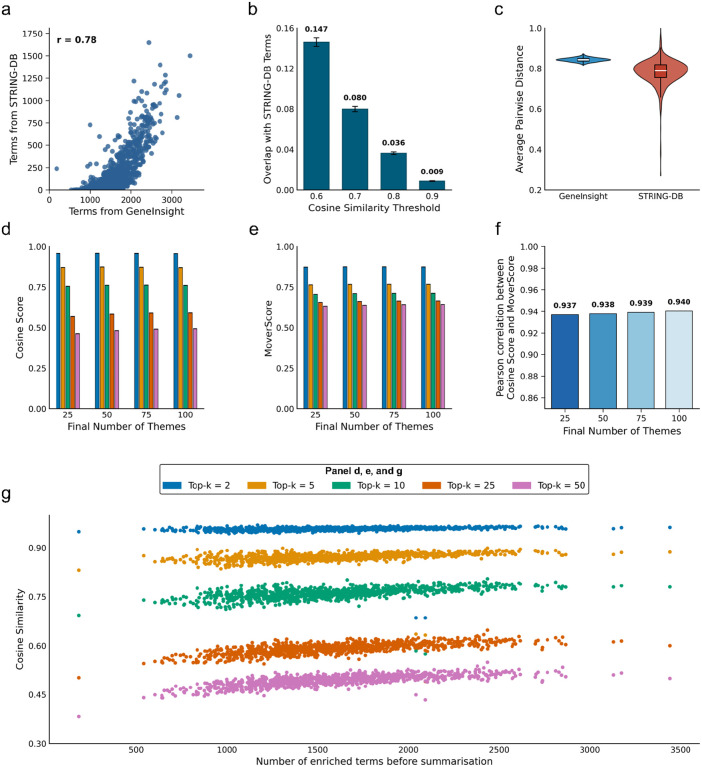
Evaluation of GeneInsight and STRING-DB. **(a)** Scatter plot showing the number of terms identified by GeneInsight versus STRING-DB API across 1,000 MSigDB gene sets. **(b)** Overlap of retained terms between GeneInsight and STRING-DB at various semantic similarity thresholds. **(c)** Average distance (AdPD) measurements comparing GeneInsight and STRING-DB enrichment results. **(d)** Top-k similarity scores for enriched gene sets across different user-defined summary levels (25, 50, 75, and 100 terms). **(e)** Top-k scores calculated using MoverScore for semantic similarity assessment. **(f)** Pearson correlation coefficients between cosine similarity and MoverScores at different levels of user-defined summarisation. **(g)** Top-k cosine similarity scores plotted against varying input corpus sizes (ranging from <500 to >3000 terms).

GeneInsight consistently identified a larger number of enriched gene sets than the STRING database functional enrichment API across all filtering thresholds examined (Figs 2a, S1). While the two methods show strong positive correlation (r = 0.69-0.87), GeneInsight retained 2–3 times more terms than the STRING-DB API at every matched setting examined (S1 Fig), indicating that topic modelling and LLM summarisation capture biological themes not identified by standard term-by-term enrichment (Fig 2b).

To evaluate the conceptual breadth of identified terms, we measured semantic diversity using average pairwise distance between terms. This metric quantifies how conceptually distinct each term is from all others in the set, with higher values indicating coverage of a broader range of biological concepts rather than redundant or closely related processes. GeneInsight-derived terms demonstrated significantly greater semantic diversity compared to STRING-DB terms (Fig 2c), indicating their ability to capture a wider spectrum of biological information.

Next, we assessed the summarisation stage of GeneInsight, which converts prioritised themes into user-defined summaries. To evaluate theme preservation during summarisation at different levels (25, 50, 75, and 100 terms), we used a Top-k semantic similarity metric that focuses on each source term’s strongest matches to measure how well summaries capture essential biological concepts without being diluted by less relevant relationships. This approach identifies the k-nearest semantic neighbours for each term, better handling the imbalance between comprehensive source material and length-constrained summaries. GeneInsight demonstrated robust summarisation of key themes across different final summary counts (Fig 2d), with the stability of these metrics suggesting that our tool effectively identifies core biological concepts regardless of user-supplied summary length constraints.

To provide an orthogonal validation beyond the cosine similarity metric, which measures the directional similarity between text representations, we employed Earth Mover’s Distance [15] (MoverScore) (Fig 2e). Like cosine distance, this complementary approach assesses semantic similarity by measuring the minimum cost required to transform one text into another in the embedding space, capturing different aspects of semantic relationships. All Top-k values demonstrated MoverScores greater than 0.5, indicating that each extracted theme successfully captured substantial semantic content from the original enriched gene sets. The results showed consistently high correlation values (Fig 2f) between MoverScores and cosine similarity scores (0.93 - 0.94) across all theme configurations, confirming the robustness of our semantic similarity assessments.

We also confirmed that summarisation performance remained stable across documents of varying sizes (from <500 to >3000 terms), demonstrating that our tool’s summarisation capability is independent of input size (Fig 2g).

Next, we applied GeneInsight to an RNA-seq dataset from a murine model of mesothelioma treated with immunotherapy, specifically focusing on genes differentially expressed in treatment responders [13]. GeneInsight prioritised biological themes centred around Type I interferon signalling and monocyte-macrophage axis activation (S1 Data) which were not detected through standard Gene Ontology (GO) enrichment analysis. The importance of Type I interferon signalling was subsequently validated through mouse models using antibody-mediated interferon blockade experiments, which confirmed the functional relevance of these pathways in treatment response. The monocyte involvement themes identified by our tool were further validated through single-cell analysis, confirming GeneInsight’s capacity to identify biologically relevant signatures that effectively bridge bulk and single-cell approaches.

We then evaluated GeneInsight on a multi-omics dataset from the DREAM study [16,17], which included mesothelioma patients undergoing chemoimmunotherapy treatment. Using responder-specific genes identified through a NanoString panel and bulk RNA-seq time course data, GeneInsight successfully extracted stem cell-like signatures in T-cells (S2 Data) that were independently confirmed through orthogonal single-cell validation. In the original analysis, these stem-like signatures were only discovered after weeks of analysis involving differential abundance testing, manual inspection of CD8^+^ T cell subclusters, differential expression analysis and marker analysis comparing responders and non-responder populations [17]. GeneInsight streamlined this process by automatically identifying these key biological themes in a single analysis of 30 minutes, demonstrating how it can reduce analytical complexity and accelerate hypothesis generation from multi-omic datasets.

Finally, we evaluated GeneInsight using a published gene set [18] associated with the transcriptional response of neutrophils to Francisella tularensis infection. GeneInsight identified distinct biological themes from differentially expressed genes involved in glucose metabolism (S3 Data), suggesting a metabolic shift that aligns with neutrophil functional alterations during infection. This metabolic reprogramming pattern, particularly involving key glycolytic regulators such as PFKL, was validated in a follow-up study [19] using the same dataset 9 years later. This case demonstrates GeneInsight’s capacity to derive novel biological insights from existing datasets, potentially accelerating discovery timelines from functional genomics data.

## Availability and future directions

GeneInsight is freely available as an open-source Python package under the MIT License at https://github.com/wlchin/geneinsight. The complete source code, documentation, installation instructions, and analysis workflows are accessible through this repository. The S1 File describes the methods associated with these analysis workflows in detail.

Several limitations should be considered when interpreting GeneInsight results. GeneInsight uses empirical p-values, calibrated against a size-stratified random-query null, as ranking scores for prioritising themes. The procedure assumes that the query genes can be treated as a random sample from a user-specified background gene set [20–22]. In practice, many biological inputs depart from this assumption. Differential-expression lists, curated pathways, STRING-derived neighbourhoods, and STRING-DB functional enrichment outputs often carry experimental, annotation, or network-derived structure that the random-query null does not model [6,20]. Gene–gene correlation, uneven annotation across genes, and overlap among functional categories can also affect calibration [20–22]. These are known limitations of over-representation analysis more broadly [23], and the empirical procedure used here calibrates the random-sampling component but not these additional sources of structure. We do not claim calibration against alternative nulls such as expression-matched or network-degree-matched randomisation. The empirical p-values should therefore be interpreted as a calibrated ranking score for prioritising themes, not as significance tests for individual genes or pathways.

GeneInsight also relies on LLM-generated summaries. Although the LLM is constrained to summarise only retrieved STRING-DB terms and the final HTML report links each theme back to its source annotations, hallucination or over-interpretation cannot be eliminated. Users should therefore verify that generated summaries accurately reflect the displayed source annotations and should treat GeneInsight outputs as hypothesis-generating summaries rather than definitive biological conclusions.

Looking ahead, GeneInsight’s algorithm is designed to process text-based annotations regardless of their source. While our current implementation uses the STRING database, the framework can readily incorporate PubMed abstracts, Gene Ontology terms, pathway descriptions from Reactome [24], and unpublished private datasets. This source-agnostic design allows GeneInsight to incorporate any text-based annotation source, providing an automated aggregation layer that synthesises diverse annotations into interpretable biological themes.

## Supporting information

S1 Fig Correlation between GeneInsight and STRING database (STRING-DB) retained gene sets across a range of filtering cut-offs, shown as a sensitivity diagnostic. The grid shows pairwise comparisons of enriched gene sets identified by GeneInsight (x-axis) versus terms from the STRING database (y-axis) at varying BH-adjusted hypergeometric p-value cut-offs (filtering thresholds, not calibrated FDR estimates). Each subplot represents a different combination of p-value thresholds, with F indicating the BH-adjusted hypergeometric p-value cut-off applied to GeneInsight results and E indicating the STRING-reported FDR threshold. Thresholds range from stringent (0.001) to permissive (0.05). Blue dots represent individual gene sets with the number of enriched terms plotted for each tool. Dashed lines show the linear regression trend. Correlation coefficients (r) are displayed in the upper left of each panel.
(PDF)

S1 Data Murine mesothelioma immunotherapy response analysis. HTML reports and raw files from a GeneInsight analysis of the murine mesothelioma immunotherapy response dataset, highlighting biological themes related to Type I interferons and monocyte-macrophage activation.
(ZIP)

S2 Data DREAM study mesothelioma patient analysis. HTML reports and raw files from a GeneInsight analysis of the DREAM study mesothelioma patient dataset, highlighting stem cell-like (lymphocyte proliferation and differentiation) signatures in T-cells of responders to chemoimmunotherapy treatment.
(ZIP)

S3 Data Neutrophil transcriptional response analysis. HTML reports and raw files from a GeneInsight analysis of neutrophil transcriptional response to Francisella tularensis infection, featuring identified metabolic reprogramming signatures in glucose metabolism.
(ZIP)

S1 File Analysis workflow methods documentation. Detailed methods associated with analysis workflows.
(DOCX)

S2 File LLM prompts. Complete prompts used for theme generation and summarisation.
(PDF)

S4 Data Murine mesothelioma immunotherapy response analysis with overlap filter. Raw files from a GeneInsight analysis with overlap ratio filter (threshold 0.25) applied to the murine mesothelioma immunotherapy response dataset.
(ZIP)

S5 Data DREAM study mesothelioma patient analysis with overlap filter. Raw files from a GeneInsight analysis with overlap ratio filter (threshold 0.25) applied to the DREAM study mesothelioma patient dataset.
(ZIP)

S6 Data Neutrophil transcriptional response analysis with overlap filter. Raw files from a GeneInsight analysis with overlap ratio filter (threshold 0.25) applied to the neutrophil transcriptional response dataset.
(ZIP)

## References

[1] Ashburner M, Ball CA, Blake JA, Botstein D, Butler H, Cherry JM. Gene Ontology: tool for the unification of biology. Nat Genet. 2000;25:25–9. doi: 10.1038/75556 10802651 PMC3037419

[2] Kanehisa M, Goto S. KEGG: kyoto encyclopedia of genes and genomes. Nucleic Acids Res. 2000;28(1):27–30. doi: 10.1093/nar/28.1.27 10592173 PMC102409

[3] Wu T, Hu E, Xu S, Chen M, Guo P, Dai Z. clusterProfiler 4.0: A universal enrichment tool for interpreting omics data. Innovation (Camb). 2021;2:100141. doi: 10.1016/j.xinn.2021.100141 34557778 PMC8454663

[4] Kuleshov MV, Jones MR, Rouillard AD, Fernandez NF, Duan Q, Wang Z, et al. Enrichr: a comprehensive gene set enrichment analysis web server 2016 update. Nucleic Acids Res. 2016;44(W1):W90-7. doi: 10.1093/nar/gkw377 27141961 PMC4987924

[5] Stathias V, Turner J, Koleti A, Vidovic D, Cooper D, Fazel-Najafabadi M. LINCS Data Portal 2.0: next generation access point for perturbation-response signatures. Nucleic Acids Research. 2020;48:D431–9. doi: 10.1093/nar/gkz1023 PMC7145650 31701147

[6] Szklarczyk D, Kirsch R, Koutrouli M, Nastou K, Mehryary F, Hachilif R, et al. The STRING database in 2023: protein-protein association networks and functional enrichment analyses for any sequenced genome of interest. Nucleic Acids Res. 2023;51(D1):D638–46. doi: 10.1093/nar/gkac1000 36370105 PMC9825434

[7] Lewis CM, Grossetti F. A statistical approach for optimal topic model identification. J Mach Learn Res. 2022;23(58):1–20.

[8] Egger R, Yu J. A topic modeling comparison between LDA, NMF, Top2Vec, and BERTopic to demystify Twitter posts. Front Sociol. 2022;7:886498. doi: 10.3389/fsoc.2022.886498 35602001 PMC9120935

[9] Naveed H, Khan AU, Qiu S, Saqib M, Anwar S, Usman M, et al. A Comprehensive Overview of Large Language Models. 2024. doi: 10.48550/arXiv.2307.06435

[10] Hu M, Alkhairy S, Lee I, Pillich RT, Fong D, Smith K, et al. Evaluation of large language models for discovery of gene set function. Nat Methods. 2025;22(1):82–91. doi: 10.1038/s41592-024-02525-x 39609565 PMC11725441

[11] Wang Z, Jin Q, Wei C-H, Tian S, Lai P-T, Zhu Q, et al. GeneAgent: self-verification language agent for gene-set analysis using domain databases. Nat Methods. 2025;22(8):1677–85. doi: 10.1038/s41592-025-02748-6 40721871 PMC12328209

[12] Zhu J, Wang RY, Wang X, Azevedo R, Moreno A, Kuhn JA, et al. Enhancing gene set overrepresentation analysis with large language models. Bioinform Adv. 2025;5(1):vbaf054. doi: 10.1093/bioadv/vbaf054 40401046 PMC12093311

[13] Zemek RM, Chin WL, Fear VS, Wylie B, Casey TH, Forbes C, et al. Temporally restricted activation of IFNβ signaling underlies response to immune checkpoint therapy in mice. Nat Commun. 2022;13(1):4895. doi: 10.1038/s41467-022-32567-8 35986006 PMC9390963

[14] Liberzon A, Birger C, Thorvaldsdóttir H, Ghandi M, Mesirov JP, Tamayo P. The Molecular Signatures Database (MSigDB) hallmark gene set collection. Cell Syst. 2015;1(6):417–25. doi: 10.1016/j.cels.2015.12.004 26771021 PMC4707969

[15] Zhao W, Peyrard M, Liu F, Gao Y, Meyer CM, Eger S. MoverScore: Text Generation Evaluating with Contextualized Embeddings and Earth Mover Distance. 2019. doi: 10.48550/arXiv.1909.02622

[16] Nowak AK, Lesterhuis WJ, Kok P-S, Brown C, Hughes BG, Karikios DJ, et al. Durvalumab with first-line chemotherapy in previously untreated malignant pleural mesothelioma (DREAM): a multicentre, single-arm, phase 2 trial with a safety run-in. Lancet Oncol. 2020;21(9):1213–23. doi: 10.1016/S1470-2045(20)30462-9 32888453

[17] Chin WL, Cook AM, Chee J, Principe N, Hoang TS, Kidman J, et al. Coupling of response biomarkers between tumor and peripheral blood in patients undergoing chemoimmunotherapy. Cell Rep Med. 2025;6(1):101882. doi: 10.1016/j.xcrm.2024.101882 39731918 PMC11866441

[18] Schwartz JT, Bandyopadhyay S, Kobayashi SD, McCracken J, Whitney AR, Deleo FR, et al. Francisella tularensis alters human neutrophil gene expression: insights into the molecular basis of delayed neutrophil apoptosis. J Innate Immun. 2013;5(2):124–36. doi: 10.1159/000342430 22986450 PMC3703778

[19] Krysa SJ, Allen L-AH. Metabolic Reprogramming Mediates Delayed Apoptosis of Human Neutrophils Infected With Francisella tularensis. Front Immunol. 2022;13:836754. doi: 10.3389/fimmu.2022.836754 35693822 PMC9174434

[20] Geistlinger L, Csaba G, Santarelli M, Ramos M, Schiffer L, Turaga N, et al. Toward a gold standard for benchmarking gene set enrichment analysis. Brief Bioinform. 2021;22(1):545–56. doi: 10.1093/bib/bbz158 32026945 PMC7820859

[21] Cao J, Zhang S. A Bayesian extension of the hypergeometric test for functional enrichment analysis. Biometrics. 2014;70(1):84–94. doi: 10.1111/biom.12122 24320951 PMC3954234

[22] Goeman JJ, Bühlmann P. Analyzing gene expression data in terms of gene sets: methodological issues. Bioinformatics. 2007;23(8):980–7. doi: 10.1093/bioinformatics/btm051 17303618

[23] Ziemann M, Schroeter B, Bora A. Two subtle problems with overrepresentation analysis. Bioinform Adv. 2024;4(1):vbae159. doi: 10.1093/bioadv/vbae159 39539946 PMC11557902

[24] Milacic M, Beavers D, Conley P, Gong C, Gillespie M, Griss J, et al. The Reactome Pathway Knowledgebase 2024. Nucleic Acids Res. 2024;52:D672–8. doi: 10.1093/nar/gkad1025 PMC10767911 37941124

