# Supplementary material for: GeneInsight: Condensing gene set knowledge via language models
Source: PLoS Comput Biol. 2026 Aug 5;22(8):e1014500. doi: 10.1371/journal.pcbi.1014500 (PMC13440848; doi:10.1371/journal.pcbi.1014500)
Supplement: Supplementary file 1 [file pcbi.1014500.s001.pdf]

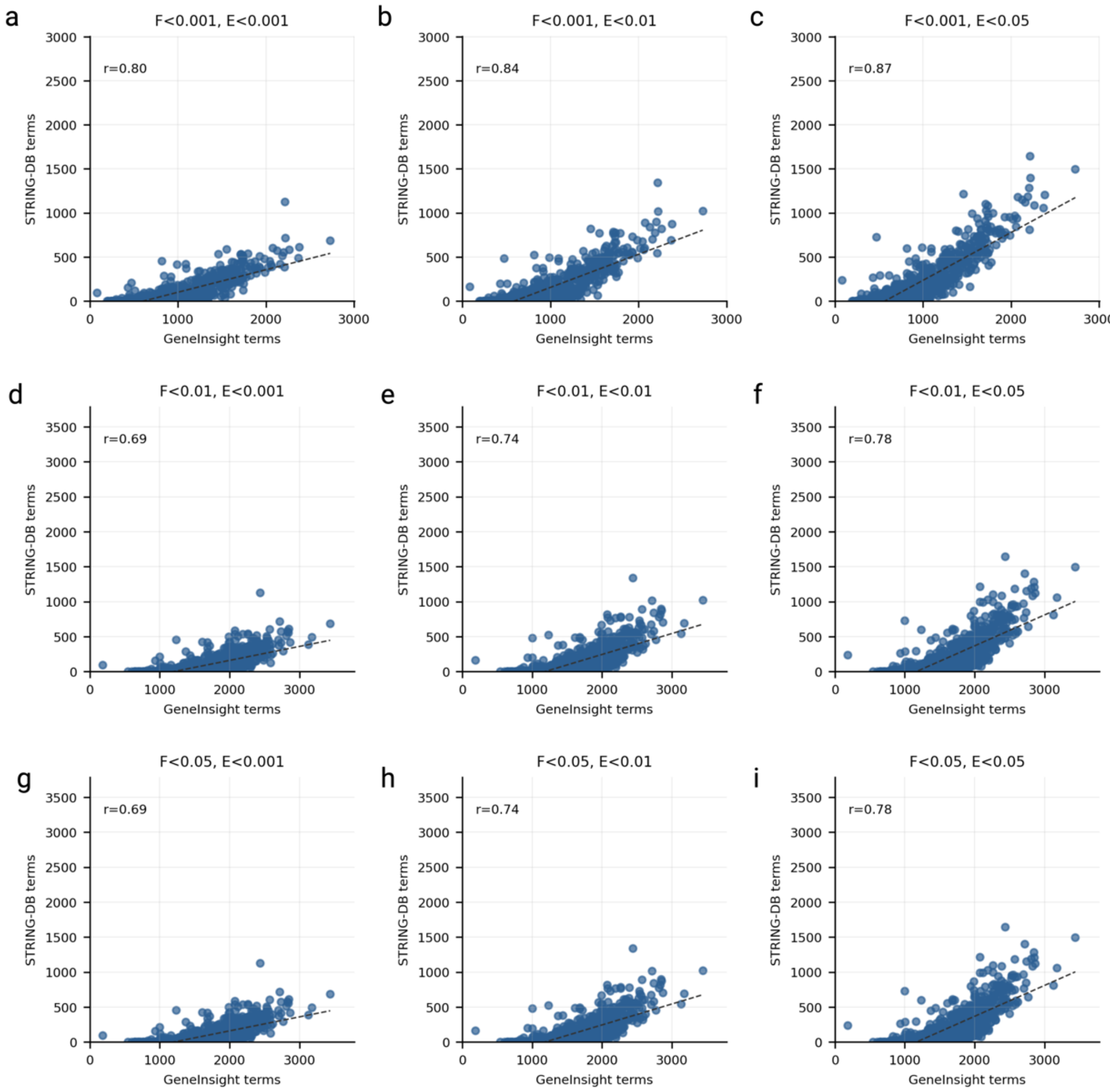

S1 Figure. Correlation between GeneInsight and the STRING database (STRING-DB) enriched gene sets at different significance thresholds. The grid shows pairwise comparisons of enriched gene sets identified by GeneInsight (x-axis) versus terms from the STRING database (y-axis) at varying statistical significance thresholds. Each subplot represents a different combination of p-value thresholds, with F indicating the adjusted p-value threshold (FDR) for GeneInsight results and E indicating the FDR threshold for STRING database results. Thresholds range from stringent (0.001) to permissive (0.05). Blue dots represent individual gene sets with the number of enriched terms plotted for each tool. Dashed lines show the linear regression trend. Correlation coefficients (r) are displayed in the upper-left of each panel.
