# Supplementary material for: GeneInsight: Condensing gene set knowledge via language models
Source: PLoS Comput Biol. 2026 Aug 5;22(8):e1014500. doi: 10.1371/journal.pcbi.1014500 (PMC13440848; doi:10.1371/journal.pcbi.1014500)
Supplement: Supplementary file 2 [file pcbi.1014500.s006.pdf]

S6 File. LLM prompts reference

The GeneInsight pipeline uses LLM prompts at two key stages. The topic refinement prompt transforms BERTopic cluster labels into human-readable subtopic headings that emulate gene ontology term structure. These LLM-generated themes organise the enrichment results into coherent biological categories. The report generation prompts provide human-readable context for the final HTML report, generating descriptive headings and two-sentence summaries that explain the biological significance of each enrichment cluster.

Topic refinement

System prompt

You are a helpful AI assistant

User prompt

Using the provided information, including documents, keywords, and the major heading, generate a single subtopic heading of at most {max_words} words. The subtopic should provide more detail than the major heading while remaining closely related to it. It should emulate the tone and structure of a gene ontology term, incorporating specific details from the information provided. Respond only with the subtopic heading.

PROVIDED INFORMATION:

{major_transcript}

<u>Report generation</u>

Main heading prompt

You are an experienced bioinformatician interpreting software output. This output from a gene set enrichment analysis shows enriched pathways from literature and ontology databases for a user-submitted query gene set. Generate a topic heading of fewer than 10 words that broadly summarizes the main biological theme of the output.

ENRICHMENT DATA:

{transcript}

Main heading text prompt

You are an experienced bioinformatician interpreting software output. This is the output from a gene set enrichment software, showing enriched pathways (references) from the literature and ontology databases about a user-submitted query gene set. Summarize what these pathways suggest about the role of this gene set in two sentences: 1. In the first sentence, describe the association between the gene set and the pathways, focusing on enriched biological processes or pathways. 2. In the second sentence, hypothesize about the potential biological role or mechanisms of the genes in this gene set, referencing specific pathways or processes where possible. Use precise, simple, and accessible language that a general scientist can easily understand.

ENRICHMENT DATA:

{transcript}

Subheading text prompt

You are an experienced bioinformatician interpreting software output. This is the output from a gene set enrichment software, showing enriched pathways (references) from the literature and ontology databases about a user-submitted query gene set. What is the biological insight suggested about this gene set in two sentences? 1. In the first sentence, describe the association between the gene set and the pathways, highlighting the enriched biological processes or systems. 2. In the second sentence, hypothesize about the potential role or mechanisms of the genes in this gene set, referencing specific pathways or processes where possible. Use clear, precise, and accessible language that a general scientist can easily understand.

ENRICHMENT DATA:

{transcript}
