# Supplementary material for: GeneInsight: Condensing gene set knowledge via language models
Source: PLoS Comput Biol. 2026 Aug 5;22(8):e1014500. doi: 10.1371/journal.pcbi.1014500 (PMC13440848; doi:10.1371/journal.pcbi.1014500)
Supplement: Supplementary file 3 [file pcbi.1014500.s005.docx]

**S1 File. Materials and methods**

**Implementation and benchmarking workflow**

GeneInsight is implemented in Python (3.10+). The workflow management system Snakemake (v7.32.4) coordinates the benchmarking pipeline, while documentation is generated using Sphinx (v7.2.6). Core computational components include BERTopic (v0.15.0) for topic modelling and Optuna (v3.3.0) for hyperparameter optimisation.

**Source data**

The STRING [1] database serves as a fundamental data source for GeneInsight, providing comprehensive protein-protein interaction (PPI) data across multiple organisms. We utilised STRING version 12.0. The database integrates both direct physical interactions and indirect functional associations, drawing from multiple evidence types including experimental data, pathway knowledge, co-expression patterns, and text mining of scientific literature.

For our analysis pipeline, we specifically leverage STRING's functional enrichment API, which provides statistically validated functional annotations for protein sets. The API accepts protein identifiers and returns enriched terms across multiple functional classification systems, including Gene Ontology (GO) terms, KEGG pathways, and UniProt keywords [2]. Each returned term is accompanied by a false discovery rate (FDR), p-value, and the number of proteins from the query set that map to that term.

**Algorithm description**

GeneInsight employs a two-stage approach for gene set analysis that processes biological information at the individual gene level before performing knowledge synthesis, offering an alternative to conventional set-level enrichment methods.

In the first stage (biological theme generation), we query the STRING functional enrichment API to retrieve associated terms for each gene in the input set based on their statistical significance. This gene-level querying, as opposed to direct set-level enrichment, allows us to capture the full spectrum of functional annotations per gene, forming a rich document corpus that preserves gene-specific context.

BERTopic (v0.15.0) then processes this corpus through cluster-based topic modelling to identify coherent biological themes by grouping similar annotations into clusters. Each topic represents a collection of functionally related terms supported by gene-specific literature evidence. A large language model (GPT-4o mini by default) converts these detailed topic representations into interpretable biological themes while maintaining the granular insights gained from per-gene analysis. The complete prompts used for theme generation and summarisation are provided in S2 File.

Each LLM-generated topic is used as a search query against the corpus. Because each document retains its link to specific genes, we build a gene set for each topic by combining genes from the top matching documents (default N=5). For each topic-associated gene set we compute a hypergeometric overlap statistic against the user-provided query genes and background set. Theme prioritisation uses the overlap-ratio filter and the size-matched empirical p-value (Empirical p-value calibration, below). The background universe used in the hypergeometric overlap calculation is user-configurable and can be matched to the biological context of the analysis, for example the genes assayed, detected, or otherwise eligible for inclusion in the input list [3].

The second stage (summarisation phase) begins with another round of cluster-based topic modelling to identify key themes. Our approach ensures robust theme identification by performing multiple independent rounds (default N=5) of topic modelling with different random seeds. The terminal condensation phase identifies stable, biologically meaningful topics that persist across initialisations by measuring how consistently they appear as cluster representatives. We identify these stable topics through cosine similarity measurements between iterations and rank them by frequency.

The final topic selection uses similarity thresholds that align with user-specified enrichment criteria and length preferences, facilitated by automatic optimisation against clustering metrics. Specifically, we employ Davies-Bouldin [4] and Calinski-Harabasz [5] scores to evaluate cluster quality, which quantify both the cohesion within clusters and the separation between them. The LLM then creates a hierarchical summary where major biological themes appear as main headings with related subheadings grouped beneath them, presented in an interactive HTML report that connects theme descriptions directly to the original gene annotations, allowing researchers to easily navigate between broad biological processes and their specific components while maintaining accuracy to the underlying gene functions.

**Pipeline Architecture**

Fig A presents a detailed view of the GeneInsight pipeline architecture, illustrating how the system processes query genes through six sequential phases. The pipeline begins with data acquisition from STRING-DB (Phase 1), followed by topic discovery and LLM refinement to generate interpretable themes (Phase 2). Theme prioritisation through overlap-ratio filtering and size-matched empirical p-value calibration identifies retained themes (Phase 3). Theme consolidation reduces redundancy through similarity filtering and hierarchical clustering (Phase 4). Ontology annotation grounds themes against Gene Ontology and Human Phenotype Ontology (Phase 5). Finally, report generation produces an interactive HTML report with heatmaps and LLM narrative summaries (Phase 6). Red boxes highlight statistical testing steps (Phases 3, 5). Teal boxes indicate topic modelling steps unique to GeneInsight.

**Fig A. GeneInsight pipeline architecture.** The pipeline processes query genes (n) against a background set (N) defined by STRING-DB for the selected organism. Phase 1 retrieves functional annotations from STRING-DB. Phase 2 applies topic modelling (BERTopic) to group annotations, with LLM refinement to generate interpretable themes; semantic search links themes back to their contributing genes. Phase 3 prioritises candidate themes using an overlap-ratio threshold combined with a size-matched empirical p-value. Phase 4 consolidates themes through a second topic modelling pass, similarity filtering, and hierarchical clustering. Phase 5 applies a second round of ORA to ground themes against Gene Ontology (GO) and Human Phenotype Ontology (HPO). Phase 6 generates an interactive HTML report where each theme displays its associated genes as expandable drop-downs alongside hypergeometric test statistics. Badges indicate steps using external APIs (API), large language models (LLM), over-representation analysis (ORA), and FDR correction (FDR). Red boxes highlight statistical testing steps (Phases 3, 5) comparable to standard ORA; teal boxes indicate topic modelling steps unique to GeneInsight.

**Overlap Ratio Filter**

GeneInsight includes an optional overlap ratio filter to improve the specificity of enrichment results. For each enriched term, the overlap ratio is calculated as the number of query genes annotated to the term divided by the total number of genes in that term’s annotation set. This metric distinguishes terms where a substantial proportion of annotated genes are present in the query (high overlap ratio) from terms that achieve statistical significance through sparse, potentially coincidental overlap (low overlap ratio). By default, terms with an overlap ratio below 0.25 are excluded, retaining only those where at least 25% of the term’s annotated genes appear in the input set. The filter operates on the hypergeometric overlap statistics and is applied alongside the size-matched empirical p-value calibration (Empirical p-value calibration, below), providing an additional specificity criterion that the p-value alone does not capture.

To validate this threshold, we compared overlap ratio distributions between curated biological gene sets from GO Biological Process and randomly generated gene sets. Random gene sets returned terms passing the BH-adjusted hypergeometric p-value cut-off, but examination revealed that significance was consistently achieved through minimal gene coverage. Random gene sets produced narrow overlap ratio distributions clustered between 0.15–0.30, whereas biological gene sets produced wider distributions (0.15–0.60) with a distinct right tail representing terms with high gene coverage (Fig B). Based on this empirical distinction, the default threshold of 0.25 effectively separates biological signal from statistical noise arising from sparse, coincidental gene overlap. The overlap ratio filter was additionally applied to the three case-study datasets; the resulting filtered outputs are provided in S4 Data (murine mesothelioma immunotherapy response), S5 Data (DREAM study mesothelioma patient analysis), and S6 Data (neutrophil transcriptional response).

**Fig B.** Overlap ratio filter validation, shown as a threshold-selection diagnostic. (A) Number of retained terms returned by random gene sets across BH-adjusted hypergeometric p-value cut-offs of 0.0001, 0.01, and 0.05. These values are used as filtering thresholds, not calibrated FDR estimates. (B) Distribution of overlap counts (number of input genes per retained term) for retained terms at a BH-adjusted hypergeometric p-value cut-off of 0.05. (C) Distribution of overlap ratios (input genes in term divided by total genes annotated to term) for retained terms at a BH-adjusted hypergeometric p-value cut-off of 0.05, comparing GOBP (GO Biological Process curated gene sets) and Random (randomly generated gene sets). The dashed line indicates the 0.25 filter threshold. (D) Kernel density estimation of mean overlap ratios per gene set for GOBP and Random groups. The dashed line indicates the 0.25 filter threshold.

**Empirical p-value calibration**

We calibrate a ranking score for prioritising candidate themes against an explicit, well-defined null. For each candidate biological term, the empirical p-value is computed against a null pool of GeneInsight outputs on random gene sets stratified by query-set size (50-gene bins from 1–50 to 451–500, evaluated against the STRING-DB universe for human and mouse). The empirical p-value is the fraction of null-set terms with a hypergeometric overlap statistic at or below the observed value, conditional on the matched size bin.

Across the full null pool of 97,920 topic-level statistics from 100 random gene-set pipeline runs, the empirical p-value is uniformly distributed under Uniform(0,1), whereas the raw hypergeometric p-value departs substantially from uniformity (Fig C). Calibration also holds within every query-size bin, confirmed by leave-one-out QQ plots stratified by 50-gene bin from 1–50 to 451–500 (Fig D). When applied to the three case studies reported in the main manuscript (murine mesothelioma immunotherapy, DREAM chemoimmunotherapy, and the neutrophil Francisella response), the empirical-p QQ curves lie below the diagonal (Fig E), indicating that the procedure behaves conservatively in practice and does not inflate apparent enrichment. Theme recovery on these case studies is unchanged by the recalibration.

The empirical p-value is the primary prioritisation statistic (p < 0.05) and is applied after the overlap-ratio filter described in the preceding section.

GeneInsight provides this procedure in two forms. A precalculated size-matched null distribution ships with the package for the default reference universes (human and mouse, against a genome-wide background), giving calibrated empirical p-values without extra computation. A command-line option generates a study-specific null matched to a user-supplied background universe, which is appropriate whenever the analysis is restricted to a non-default universe such as the genes assayed, detected, or otherwise eligible for inclusion in the input list [3]. The precalculated null is valid only against its associated background, and applying it to a mismatched background is not calibrated. This procedure calibrates against a gene-sampling null only and does not address intergenic correlation, annotation-density bias, or selection structure in the query set.

**Fig C.** **Empirical recalibration restores Uniform(0,1) calibration of GeneInsight p-values.** Quantile–quantile plots of the raw hypergeometric p-values and the empirically recalibrated p-values, computed against a null pool of 97,920 topic-level statistics from 100 random gene-set pipeline runs (ten 50-gene-wide query-size bins). (A) Linear axes. (B) Log–log axes. The dashed grey line is Uniform(0,1) (y = x).

**Fig D**. **The empirical-p recalibration is calibrated within every query-size bin.** Leave-one-out QQ plots against Uniform(0,1) for raw hypergeometric P-values (red) and empirically recalibrated p-values (navy) stratified by query gene-set size into ten 50-gene-wide bins from 1–50 to 451–500 (panels A–J). The dashed grey line indicates Uniform(0,1), y = x. Each panel's centred header reports the bin range and the number of leave-one-out null topics N; the inset reports the bin-specific Kolmogorov–Smirnov distance against Uniform(0,1) for both traces.

**Fig E.** **Empirical p preserves recovered biology on three case-study queries (S1 Data, S2 Data, S3 Data).** Per-topic empirical p-value histograms (A–C) and QQ plots against Uniform(0,1) (D–F) for S1 (Murine Mesothelioma Immunotherapy), S2 (DREAM Chemoimmunotherapy) and S3 (Neutrophil Francisella Response). Empirical p was computed by ranking each topic's raw hypergeometric p against a topic null pool from 100 random pipeline runs across ten size-stratified bins (Methods). Insets report the topic count N, fraction passing p ≤ 0.05, Storey π̂₀, and KS distance against Uniform(0,1).

**Semantic Overlap Metric for Biological Term Sets**

We developed a semantic overlap metric to quantify relationships between biological term sets, defined as SC(A,B) = Σ max(cos(e_a_,e_b_)) for all a ∈ A, b ∈ B, where e_a_ and e_b_ represent embedding vectors of terms from sets A and B respectively, and cos denotes cosine similarity. This metric allows semantically similar phrases to be counted as overlapping, rather than requiring exact matches. To enable comparison across datasets of varying sizes, we implemented a normalised form NSC(A,B) = SC(A,B) / |A|, where |A| represents the cardinality of set A. Dense vector representations were generated using the SentenceTransformer framework with the paraphrase-MiniLM-L6-v2, producing 384-dimensional embeddings that capture the contextual relationships between terms.

We applied this metric in two contexts: first, to optimize BERTopic hyperparameters by comparing different numbers of sampling rounds during the first phase (n = 1 to n = 10) and assessing the stability of the resulting term sets; and second, to evaluate the overlap between GeneInsight-derived themes and conventional STRING-DB functional enrichment results, providing quantitative measures of concordance between these different approaches (Fig. 2b, manuscript).

**Top-k Semantic Similarity Analysis**

Assessing theme preservation in biological term sets requires robust quantification of meaningful relationships while accounting for the inherent variability in gene set annotations. To evaluate how well summaries preserve core themes from source terms, we developed a recall-based similarity metric that captures nuanced relationships beyond exact-match approaches. This metric specifically measures how well the summary captures essential ideas from the source material, rather than selective details. Low recall would indicate missing themes, while high recall ensures comprehensive preservation of key biological concepts.

For any two sets of terms A = {a₁, a₂, ..., aₙ} and B = {b₁, b₂, ..., bₘ}, we compute dense vector representations using the all-MiniLM-L6-v2 model, generating embedding matrices E_A_ ∈ ℝⁿˣᵈ and E_B_ ∈ ℝᵐˣᵈ, where d = 384 represents the embedding dimension. For each term i in set A, we compute its k highest similarity scores with terms in set B using cosine similarity: TopK(i,B) = max_k_{cos(e_A_ᵢ, e_B_ⱼ) | j ∈ [1,m]}. The overall similarity score is calculated as S(A,B) = [1/n]∑ᵢ₌₁ⁿ mean(TopK(i,B)), where the mean averages the k highest similarities for each term.

We evaluate different k values (k = 2, 5, 10, 25, 50) to provide complementary views of how well meaning is retained between term sets. Lower k values focus on the strongest conceptual matches, while higher values assess broader thematic coverage. This multi-k assessment strategy reveals whether high similarity scores are maintained across different matching thresholds.

The Top-k approach offers several advantages over simple average similarity. By focusing on each source term's strongest matches rather than averaging over all pairwise comparisons, the method prevents dilution of the most meaningful relationships and ensures that high similarity scores from key themes drive the overall metric. This approach improves robustness to noise and outliers by considering only the TopK similarities for each term, making the metric less sensitive to low-scoring, less relevant matches. The method is particularly effective for handling imbalanced sets, where many terms in a larger set may not have close equivalents in the summary. By focusing solely on the strongest conceptual relationships, the approach provides a clearer assessment of how well the summary captures essential content.

The multi-threshold analysis using different k values provides insights into both the strongest matches and how comprehensively the final HTML summary retains information across user-defined length constraints. We specifically evaluated summaries containing between 25 and 100 terms, assessing how effectively these concise summaries maintained the core biological concepts from the more extensive source material. This approach is particularly advantageous when evaluating how well a summary preserves core themes from a larger set, as it emphasises conceptually significant matches, reduces noise from irrelevant pairings, and effectively handles set size imbalances inherent in summarisation tasks with strict length limitations.

**Hyperparameter Optimisation**

We utilised the Molecular Signatures Database (MSigDB) C2 collection as our primary data source for both validation and testing. This collection provides a comprehensive resource of curated gene sets derived from pathway databases, published experiments, and expert knowledge. Each gene set includes detailed text descriptions of its experimental derivation, offering both transcriptomic and semantic information. We randomly selected 100 gene sets for validation and 1000 distinct gene sets for testing, ensuring comprehensive coverage across diverse biological processes and experimental conditions.

Hyperparameter optimisation on the validation set focused on two critical aspects of GeneInsight's implementation: the biological theme identification (theme generation phase) parameters (Fig F, panels a–d) and the rank-based clustering iterations (summarisation phase, Fig F, panels e–g). For the biological theme identification phase, we employed an elbow method using soft cardinality intersection against a term universe generated through exhaustive sampling across 10 initialisation seeds across 1–10 sampling runs. For the summarisation phase, we evaluated convergence using rank-based metrics with an upper bound of 25 biological theme identification runs. Through systematic evaluation of cosine similarity thresholds and soft overlap intersection measures, we determined optimal cut-off points based on clustering stability analysis. This process resulted in default values of 5 sampling rounds and 10 rank sampling rounds, which were selected based on their empirical performance on the validation set.

**Fig F. Convergence analysis of topic modelling stability with increasing number of runs.** Top panels (a-d): Normalised soft cardinality measures the semantic overlap between terms identified in each individual topic modelling run and the complete term set across all runs. Higher values indicate greater coverage of the overall vocabulary space. Bottom panels (e-g): Box plots show three complementary ranking metrics comparing each run (1-20) against the reference (25 runs): Panel e (Rank-Biased Overlap, RBO), Panel f (Kendall Distance), and Panel g (Kendall's τ correlation). Higher RBO and Kendall's τ values indicate greater similarity to reference rankings, while lower Kendall Distance values represent better alignment. For all panels, trend lines connect median values, showing the stabilization pattern as the number of topic modelling runs increases. Box plots show distribution (centre line = median, box limits = first and third quartiles, whiskers = 1.5× interquartile range, points = outliers).

**Comparative evaluation**

For comparative evaluation against conventional approaches, we assessed GeneInsight's performance against STRING-DB functional enrichment using an independent test set of 1000 MSigDB gene sets. Enrichment analysis was performed using STRING-DB (version 12.0). For each gene set, we computed three complementary metrics to evaluate term diversity and semantic preservation.

Term diversity was quantified using Average Pairwise Distance (APD) between semantic embeddings. For each term set, we generated dense vector representations using the all-MiniLM-L6-v2 model (384-dimensional embeddings) and computed pairwise cosine distances. The APD was calculated as the mean of these distances, with higher values indicating greater semantic diversity. Statistical significance of the differences between GeneInsight and STRING-DB results was determined using a two-sided t-test.

Semantic preservation was assessed using the Top-k similarity metric (described above), where for each source term, we identified the k most semantically similar terms in the summarised set (k = 2, 5, 10, 25, 50) using cosine similarity between embedding vectors. As an orthogonal validation approach complementary to cosine similarity but not incorporated into the GeneInsight software itself, we also evaluated performance using the MoverScore, which measures optimal transport distance between term embeddings and accounts for both semantic and positional alignment.

Performance was evaluated across different user-specified biological theme counts in the final HTML report (25, 50, 75, and 100 themes) to assess how well the key biological concepts were maintained at various summary lengths.

**Computational Performance**

We evaluated computational time as a function of gene set size using 1,000 MSigDB gene sets. Processing time increases with the number of input genes, reaching approximately 40 minutes for gene sets of 400–500 genes (Fig G). These results demonstrate that the pipeline remains computationally tractable for gene set sizes routinely encountered in functional genomics workflows.

**Fig G. Computational time analysis.** Mean analysis duration (seconds) per gene set size bin (1–10 to 401–500 genes). Error bars represent standard error.

**API Usage and Cost**

We evaluated token usage and associated costs across gene set sizes using the same 1,000 MSigDB gene sets. Based on current OpenAI API pricing for GPT-4o-mini ($0.15 per million input tokens, $0.60 per million output tokens), analysis costs range from approximately $0.01–0.22 USD for gene sets of 10–500 genes depending on the number of annotations retrieved (Fig H).

**Fig H. Token usage and cost analysis.** (A) Mean token usage (prompt and completion tokens combined) per analysis by gene set size. (B) Mean cost (USD) per analysis by gene set size. Error bars represent standard error. Gene sets ranged from 1–10 to 401–500 genes. Costs calculated using GPT-4o-mini pricing.

**Dataset Analyses**

The runs were performed using default parameters for topic modelling with a specified summary length of 100 terms and a temperature parameter of 0.0, followed by manual inspection of enriched terms and the HTML summary.

For the first dataset, we analysed a collection of "fast-off" genes as defined in a time-course analysis [6] of the AB1 mesothelioma mouse model across four time points following immune checkpoint blockade administration.

In our second dataset, we analysed RNA-seq data from mesothelioma patients which included time-course expression profiles, as described in the Phase II DREAM [7] chemo-immunotherapy study. We specifically focused on the first 100 genes identified as important responders based on their factor loading scores. Analysis was performed using DIABLO [8] , which enabled the identification of key predictors from multi-omic data.

For the third dataset, we analysed a published gene set [9] associated with the transcriptional response of neutrophils to Francisella tularensis infection (GSE37416). This gene set specifically comprised downregulated genes identified from human polymorphonuclear leukocytes (PMN) treated with F. tularensis subsp. holarctica live vaccine strain (LVS) for 48 hours compared to untreated controls at 0 hours.

**References**

1.	Szklarczyk D, Kirsch R, Koutrouli M, Nastou K, Mehryary F, Hachilif R, et al. The STRING database in 2023: protein-protein association networks and functional enrichment analyses for any sequenced genome of interest. Nucleic Acids Res. 2023;51: D638–D646. doi:10.1093/nar/gkac1000

2.	UniProt Consortium. UniProt: the Universal Protein Knowledgebase in 2025. Nucleic Acids Res. 2025;53: D609–D617. doi:10.1093/nar/gkae1010

3.	Ziemann M, Schroeter B, Bora A. Two subtle problems with overrepresentation analysis. Bioinformatics Advances. 2024;4(1):vbae159. doi:10.1093/bioadv/vbae159

4.	Davies DL, Bouldin DW. A Cluster Separation Measure. IEEE Transactions on Pattern Analysis and Machine Intelligence. 1979;PAMI-1: 224–227. doi:10.1109/TPAMI.1979.4766909

5.	Caliński T, and Harabasz J. A dendrite method for cluster analysis. Communications in Statistics. 1974;3: 1–27. doi:10.1080/03610927408827101

6.	Zemek RM, Chin WL, Fear VS, Wylie B, Casey TH, Forbes C, et al. Temporally restricted activation of IFNβ signaling underlies response to immune checkpoint therapy in mice. Nat Commun. 2022;13: 4895. doi:10.1038/s41467-022-32567-8

7.	Nowak AK, Lesterhuis WJ, Kok P-S, Brown C, Hughes BG, Karikios DJ, et al. Durvalumab with first-line chemotherapy in previously untreated malignant pleural mesothelioma (DREAM): a multicentre, single-arm, phase 2 trial with a safety run-in. The Lancet Oncology. 2020;21: 1213–1223. doi:10.1016/S1470-2045(20)30462-9

8.	Singh A, Shannon CP, Gautier B, Rohart F, Vacher M, Tebbutt SJ, et al. DIABLO: an integrative approach for identifying key molecular drivers from multi-omics assays. Bioinformatics. 2019;35: 3055–3062. doi:10.1093/bioinformatics/bty1054

9.	Schwartz JT, Bandyopadhyay S, Kobayashi SD, McCracken J, Whitney AR, DeLeo FR, et al. Francisella tularensis Alters Human Neutrophil Gene Expression: Insights into the Molecular Basis of Delayed Neutrophil Apoptosis. J Innate Immun. 2013;5: 124–136. doi:10.1159/000342430
