# Supplementary material for: GeneInsight: Condensing gene set knowledge via language models
Source: PLoS Comput Biol. 2026 Aug 5;22(8):e1014500. doi: 10.1371/journal.pcbi.1014500 (PMC13440848; doi:10.1371/journal.pcbi.1014500)
Supplement: Supplementary file 4 [file pcbi.1014500.s002.zip › S2 Data/sphinx_builds.zip!/sphinx_builds/html_build_geneset2/build/html/cluster_7.html]

Theme 8 - Organic Nitrogen Compound Metabolism — GeneInsight documentation


Skip to content

GeneInsight


menu

Major themes:

- Theme 1 - Inflammation and Cell Proliferation Pathways
- Theme 2 - Innate Immune Responses to Viral Infections
- Theme 3 - Insulin and Cardiomyopathy Pathways in Disease
- Theme 4 - Transcription Regulation and Metal Complex Assembly
- Theme 5 - Immune Response and Macrophage Activation
- Theme 6 - Antigen Presenting Cell Morphology and Function
- Theme 7 - Regulation of TLR and Interferon Signaling
- Theme 8 - Organic Nitrogen Compound Metabolism
- Theme 9 - Innate Immunity in Viral Infections
- Theme 10 - CD15 Neutrophils in Colorectal Cancer

Metadata:

- Embedding map
- Summary statistics
- Data Files
- Downloads


Toggle menu contents

1. Docs
2. Theme 8 - Organic Nitrogen Compound Metabolism

JavaScript is required to toggle light/dark mode..


 Light mode
 Dark mode


Edit on GitHub


View source

---

# Theme 8 - Organic Nitrogen Compound Metabolism¶

The gene set is significantly associated with organic nitrogen compound biosynthetic metabolic processes and cyclic organic compound metabolism, indicating its involvement in the synthesis and regulation of essential nitrogen-containing and cyclic organic compounds. This suggests that the genes within this set may play critical roles in nitrogen metabolism and cellular responses to environmental changes, influencing growth, development, and stress management.

## Organic nitrogen compound biosynthetic metabolic processes¶

Key information

Key genes: Adar, Dhx9, Cul4a, Rnaseh2b, Ddb1

Odds Ratio: 72.11

FDR: 0.000

Combined Score: 1468.76

The gene set is significantly associated with organic nitrogen compound biosynthetic metabolic processes, indicating its involvement in the synthesis of nitrogen-containing compounds essential for cellular functions. This suggests that the genes within this set may play critical roles in nitrogen metabolism, potentially influencing cellular growth and development through their participation in various nitrogen compound biosynthetic pathways.

StringDB references

- Nitrogen compound metabolic process (GO:0006807)
- Cellular nitrogen compound biosynthetic process (GO:0044271)

Ontology genesets

- Macromolecule Biosynthetic Process (GO:0009059)
- Tryptophan Metabolic Process (GO:0006568)
- Peptide Biosynthetic Process (GO:0043043)
- Aromatic Amino Acid Family Catabolic Process (GO:0009074)
- RNA Metabolic Process (GO:0016070)

Gene overlap matrix

Gene overlap matrix

## Cyclic Organic Compound Metabolism in Cellular Processes¶

Key information

Key genes: Maoa, Cyp1a1, Tph2, Cyp1b1, Kynu

Odds Ratio: 82.86

FDR: 0.000

Combined Score: 910.61

The gene set is significantly associated with pathways involved in the metabolism of cyclic organic compounds, indicating its role in various cellular processes such as the catabolism and response to these compounds. This suggests that the genes in this set may be crucial for regulating metabolic pathways that manage organic cyclic compounds, potentially influencing cellular responses to environmental changes or stressors.

StringDB references

- Cellular response to organic cyclic compound (GO:0071407)
- Organic cyclic compound metabolic process (GO:1901360)
- Organic cyclic compound catabolic process (GO:1901361)
- Organic hydroxy compound metabolic process (GO:1901615)

Ontology genesets

- Kynurenine Metabolic Process (GO:0070189)
- Tryptophan Metabolic Process (GO:0006568)
- Aromatic Amino Acid Family Catabolic Process (GO:0009074)
- Tryptophan Catabolic Process (GO:0006569)
- Cellular Response To Growth Hormone Stimulus (GO:0071378)

Gene overlap matrix

Gene overlap matrix


← Theme 7 - Regulation of TLR and Interferon Signaling

Theme 9 - Innate Immunity in Viral Infections  →

Page contents

- Theme 8 - Organic Nitrogen Compound Metabolism
  - Organic nitrogen compound biosynthetic metabolic processes
  - Cyclic Organic Compound Metabolism in Cellular Processes

- Contact
- Developers
