# Supplementary material for: GeneInsight: Condensing gene set knowledge via language models
Source: PLoS Comput Biol. 2026 Aug 5;22(8):e1014500. doi: 10.1371/journal.pcbi.1014500 (PMC13440848; doi:10.1371/journal.pcbi.1014500)
Supplement: Supplementary file 4 [file pcbi.1014500.s002.zip › S2 Data/sphinx_builds.zip!/sphinx_builds/html_build_geneset2/build/html/summary.html]

Embedding map — GeneInsight documentation


Skip to content

GeneInsight


menu

Major themes:

- Theme 1 - Inflammation and Cell Proliferation Pathways
- Theme 2 - Innate Immune Responses to Viral Infections
- Theme 3 - Insulin and Cardiomyopathy Pathways in Disease
- Theme 4 - Transcription Regulation and Metal Complex Assembly
- Theme 5 - Immune Response and Macrophage Activation
- Theme 6 - Antigen Presenting Cell Morphology and Function
- Theme 7 - Regulation of TLR and Interferon Signaling
- Theme 8 - Organic Nitrogen Compound Metabolism
- Theme 9 - Innate Immunity in Viral Infections
- Theme 10 - CD15 Neutrophils in Colorectal Cancer

Metadata:

- Embedding map
- Summary statistics
- Data Files
- Downloads


Toggle menu contents

1. Docs
2. Embedding map

JavaScript is required to toggle light/dark mode..


 Light mode
 Dark mode


Edit on GitHub


View source

---

# Embedding map¶

This figure is a two-dimensional map where each theme heading is positioned based on the combined semantic information from the main topics and all enriched gene set themes.

# Summary statistics¶

The following table provides a summary of the key statistics derived from the analysis:

| Statistic | Value |
| --- | --- |
| Number of genes considered | 100 |
| Documents considered | 17258 |
| Average topics | 9 |
| Range of max topics | 1 to 13 |
| Api calls made | 3183 |
| Number of themes after filtering | 25 |
| Number of clusters | 10 |
| Compression ratio | 690.32 |
| Time of analysis | 2025-04-17T15:00:16.742108 |

# Data Files¶

The enriched gene sets represent all AI-generated themes, without condensing and filtering. The subheading data represents a detailed breakdown and information of all API calls for subheading generation.

- `enriched_genesets.csv`
- `subheading_data.csv`


← Theme 10 - CD15 Neutrophils in Colorectal Cancer

Downloads  →

Page contents

- Embedding map
- Summary statistics
- Data Files

- Contact
- Developers
