# Supplementary material for: GeneInsight: Condensing gene set knowledge via language models
Source: PLoS Comput Biol. 2026 Aug 5;22(8):e1014500. doi: 10.1371/journal.pcbi.1014500 (PMC13440848; doi:10.1371/journal.pcbi.1014500)
Supplement: Supplementary file 4 [file pcbi.1014500.s002.zip › S2 Data/sphinx_builds.zip!/sphinx_builds/html_build_geneset2/build/html/cluster_9.html]

Theme 10 - CD15 Neutrophils in Colorectal Cancer — GeneInsight documentation


Skip to content

GeneInsight


menu

Major themes:

- Theme 1 - Inflammation and Cell Proliferation Pathways
- Theme 2 - Innate Immune Responses to Viral Infections
- Theme 3 - Insulin and Cardiomyopathy Pathways in Disease
- Theme 4 - Transcription Regulation and Metal Complex Assembly
- Theme 5 - Immune Response and Macrophage Activation
- Theme 6 - Antigen Presenting Cell Morphology and Function
- Theme 7 - Regulation of TLR and Interferon Signaling
- Theme 8 - Organic Nitrogen Compound Metabolism
- Theme 9 - Innate Immunity in Viral Infections
- Theme 10 - CD15 Neutrophils in Colorectal Cancer

Metadata:

- Embedding map
- Summary statistics
- Data Files
- Downloads


Toggle menu contents

1. Docs
2. Theme 10 - CD15 Neutrophils in Colorectal Cancer

JavaScript is required to toggle light/dark mode..


 Light mode
 Dark mode


Edit on GitHub


View source

---

# Theme 10 - CD15 Neutrophils in Colorectal Cancer¶

The gene set is significantly associated with pathways related to neutrophil biology and colorectal cancer progression, highlighting the role of CD15 neutrophils in influencing tumor microenvironments and clinical outcomes. These genes may facilitate immune responses and inflammation through mechanisms involving cytokines and chemokines, potentially impacting tumor growth and metastasis.

## Role of CD15 Neutrophils in Colorectal Cancer Progression¶

Key information

Key genes: Ccr3, Cxcr3, Cxcr4, Ccr5, Ccr2

Odds Ratio: 54.88

FDR: 0.000

Combined Score: 707.84

The gene set is significantly associated with pathways related to neutrophil biology and colorectal cancer progression, highlighting the role of CD15 neutrophils in influencing tumor microenvironments and clinical outcomes. These genes may facilitate immune responses and inflammation through mechanisms involving cytokines and chemokines, potentially impacting tumor growth and metastasis.

StringDB references

- Receptor-like tyrosine phosphatases CD45 and CD148 have distinct functions in chemoattractant-mediated neutrophil migration and response to S. aureus. (PMID:22078799)
- Interleukin-15 in the treatment of cancer. (PMID:25359408)
- Multiple Roles for Chemokines in Neutrophil Biology. (PMID:32733442)
- GZMK``(high) <Unknown identifier format: high>`\_ <Unknown identifier format: high>`\_ CD8`(+) <Unknown identifier format: +>`\_ T effector memory cells are associated with CD15``(high) <Unknown identifier format: high>`\_ <Unknown identifier format: high>`\_ neutrophil abundance in non-metastatic colorectal tumors and predict poor clinical outcome. (PMID:36347862)
- The Cytokine Network in Colorectal Cancer: Implications for New Treatment Strategies. (PMID:36611932)

Ontology genesets

- Neutrophil Chemotaxis (GO:0030593)
- Neutrophil Activation (GO:0042119)
- Neutrophil Migration (GO:1990266)
- Cellular Response To Tumor Necrosis Factor (GO:0071356)
- Positive Regulation Of Leukocyte Chemotaxis (GO:0002690)

Gene overlap matrix

Gene overlap matrix


← Theme 9 - Innate Immunity in Viral Infections

Embedding map  →

Page contents

- Theme 10 - CD15 Neutrophils in Colorectal Cancer
  - Role of CD15 Neutrophils in Colorectal Cancer Progression

- Contact
- Developers
