# Supplementary material for: GeneInsight: Condensing gene set knowledge via language models
Source: PLoS Comput Biol. 2026 Aug 5;22(8):e1014500. doi: 10.1371/journal.pcbi.1014500 (PMC13440848; doi:10.1371/journal.pcbi.1014500)
Supplement: Supplementary file 4 [file pcbi.1014500.s002.zip › S2 Data/sphinx_builds.zip!/sphinx_builds/html_build_geneset2/build/html/download.html]

Downloads — GeneInsight documentation


Skip to content

GeneInsight


menu

Major themes:

- Theme 1 - Inflammation and Cell Proliferation Pathways
- Theme 2 - Innate Immune Responses to Viral Infections
- Theme 3 - Insulin and Cardiomyopathy Pathways in Disease
- Theme 4 - Transcription Regulation and Metal Complex Assembly
- Theme 5 - Immune Response and Macrophage Activation
- Theme 6 - Antigen Presenting Cell Morphology and Function
- Theme 7 - Regulation of TLR and Interferon Signaling
- Theme 8 - Organic Nitrogen Compound Metabolism
- Theme 9 - Innate Immunity in Viral Infections
- Theme 10 - CD15 Neutrophils in Colorectal Cancer

Metadata:

- Embedding map
- Summary statistics
- Data Files
- Downloads


Toggle menu contents

1. Docs
2. Downloads

JavaScript is required to toggle light/dark mode..


 Light mode
 Dark mode


Edit on GitHub


View source

---

# Downloads¶

Select the themes you want to download by checking the boxes. Click “Download Selected” to get a ZIP file of the selected themes.

0

Themes selected

Theme 1 - Inflammation and Cell Proliferation Pathways

Theme 2 - Innate Immune Responses to Viral Infections

Theme 3 - Insulin and Cardiomyopathy Pathways in Disease

Theme 4 - Transcription Regulation and Metal Complex Assembly

Theme 5 - Immune Response and Macrophage Activation

Theme 6 - Antigen Presenting Cell Morphology and Function

Theme 7 - Regulation of TLR and Interferon Signaling

Theme 8 - Organic Nitrogen Compound Metabolism

Theme 9 - Innate Immunity in Viral Infections

Theme 10 - CD15 Neutrophils in Colorectal Cancer

Select All Themes
Download Selected


← Embedding map

- Contact
- Developers
