# Supplementary material for: GeneInsight: Condensing gene set knowledge via language models
Source: PLoS Comput Biol. 2026 Aug 5;22(8):e1014500. doi: 10.1371/journal.pcbi.1014500 (PMC13440848; doi:10.1371/journal.pcbi.1014500)
Supplement: Supplementary file 4 [file pcbi.1014500.s002.zip › S2 Data/sphinx_builds.zip!/sphinx_builds/html_build_geneset2/build/html/cluster_2.html]

Theme 3 - Insulin and Cardiomyopathy Pathways in Disease — GeneInsight documentation


Skip to content

GeneInsight


menu

Major themes:

- Theme 1 - Inflammation and Cell Proliferation Pathways
- Theme 2 - Innate Immune Responses to Viral Infections
- Theme 3 - Insulin and Cardiomyopathy Pathways in Disease
- Theme 4 - Transcription Regulation and Metal Complex Assembly
- Theme 5 - Immune Response and Macrophage Activation
- Theme 6 - Antigen Presenting Cell Morphology and Function
- Theme 7 - Regulation of TLR and Interferon Signaling
- Theme 8 - Organic Nitrogen Compound Metabolism
- Theme 9 - Innate Immunity in Viral Infections
- Theme 10 - CD15 Neutrophils in Colorectal Cancer

Metadata:

- Embedding map
- Summary statistics
- Data Files
- Downloads


Toggle menu contents

1. Docs
2. Theme 3 - Insulin and Cardiomyopathy Pathways in Disease

JavaScript is required to toggle light/dark mode..


 Light mode
 Dark mode


Edit on GitHub


View source

---

# Theme 3 - Insulin and Cardiomyopathy Pathways in Disease¶

The gene set is significantly associated with the insulin signaling pathway and leptin sensitivity, indicating its involvement in metabolic processes related to diabetes. These genes may play a crucial role in modulating insulin and leptin signaling, potentially influencing energy homeostasis and the development of leptin resistance.

## Insulin Signaling Pathway in Diabetes and Leptin Sensitivity¶

Key information

Key genes: Jak2, Irs2, Socs1, Stat3, Pten

Odds Ratio: 164.56

FDR: 0.000

Combined Score: 1436.07

The gene set is significantly associated with the insulin signaling pathway and leptin sensitivity, indicating its involvement in metabolic processes related to diabetes. These genes may play a crucial role in modulating insulin and leptin signaling, potentially influencing energy homeostasis and the development of leptin resistance.

StringDB references

- Leptin signaling and leptin resistance. (PMID:23580174)
- Leptin-insulin signaling overlap (WP578)
- Insulin signaling pathway (mmu04910)

Ontology genesets

- Regulation Of Insulin Receptor Signaling Pathway (GO:0046626)
- Regulation Of Glucose Import (GO:0046324)
- Positive Regulation Of Glucose Import (GO:0046326)
- Growth Hormone Receptor Signaling Pathway (GO:0060396)
- Receptor Signaling Pathway Via STAT (GO:0097696)

Gene overlap matrix

Gene overlap matrix

## Molecular Mechanisms and Pathways in Cardiomyopathy Development¶

Key information

Key genes: Sod2, Sod1, Ppargc1a, Gbp9, Iigp1

Odds Ratio: 71.24

FDR: 0.000

Combined Score: 987.10

The gene set is significantly associated with pathways involved in cardiomyopathy development, particularly highlighting the roles of inflammation, oxidative stress, and protein aggregation in various forms of the disease. These genes may contribute to the pathogenesis of cardiomyopathy through mechanisms such as promoting inflammatory responses and mitochondrial dysfunction, which are critical in the progression of conditions like arrhythmogenic and metabolic cardiomyopathy.

StringDB references

- Etiology of alcoholic cardiomyopathy: Mitochondria, oxidative stress and apoptosis. (PMID:28606389)
- Molecular dissection of Chagas induced cardiomyopathy reveals central disease associated and druggable signaling pathways. (PMID:32433643)
- Inflammation shapes pathogenesis of murine arrhythmogenic cardiomyopathy. (PMID:32529556)
- Protein Aggregation Is an Early Manifestation of Phospholamban p.`(Arg14del) <Unknown identifier format: Arg14del>`\_-Related Cardiomyopathy: Development of PLN-R14del-Related Cardiomyopathy. (PMID:34587756)
- Inflammation in Metabolic Cardiomyopathy. (PMID:34671656)

Ontology genesets

- Positive Regulation Of MAPK Cascade (GO:0043410)
- Regulation Of NMDA Receptor Activity (GO:2000310)
- Positive Regulation Of Cell Migration (GO:0030335)
- Positive Regulation Of Multicellular Organismal Process (GO:0051240)
- Regulation Of Oxidative Stress-Induced Intrinsic Apoptotic Signaling Pathway (GO:1902175)

Gene overlap matrix

Gene overlap matrix


← Theme 2 - Innate Immune Responses to Viral Infections

Theme 4 - Transcription Regulation and Metal Complex Assembly  →

Page contents

- Theme 3 - Insulin and Cardiomyopathy Pathways in Disease
  - Insulin Signaling Pathway in Diabetes and Leptin Sensitivity
  - Molecular Mechanisms and Pathways in Cardiomyopathy Development

- Contact
- Developers
