# Supplementary material for: GeneInsight: Condensing gene set knowledge via language models
Source: PLoS Comput Biol. 2026 Aug 5;22(8):e1014500. doi: 10.1371/journal.pcbi.1014500 (PMC13440848; doi:10.1371/journal.pcbi.1014500)
Supplement: Supplementary file 4 [file pcbi.1014500.s002.zip › S2 Data/sphinx_builds.zip!/sphinx_builds/html_build_geneset2/build/html/cluster_1.html]

Theme 2 - Innate Immune Responses to Viral Infections — GeneInsight documentation


Skip to content

GeneInsight


menu

Major themes:

- Theme 1 - Inflammation and Cell Proliferation Pathways
- Theme 2 - Innate Immune Responses to Viral Infections
- Theme 3 - Insulin and Cardiomyopathy Pathways in Disease
- Theme 4 - Transcription Regulation and Metal Complex Assembly
- Theme 5 - Immune Response and Macrophage Activation
- Theme 6 - Antigen Presenting Cell Morphology and Function
- Theme 7 - Regulation of TLR and Interferon Signaling
- Theme 8 - Organic Nitrogen Compound Metabolism
- Theme 9 - Innate Immunity in Viral Infections
- Theme 10 - CD15 Neutrophils in Colorectal Cancer

Metadata:

- Embedding map
- Summary statistics
- Data Files
- Downloads


Toggle menu contents

1. Docs
2. Theme 2 - Innate Immune Responses to Viral Infections

JavaScript is required to toggle light/dark mode..


 Light mode
 Dark mode


Edit on GitHub


View source

---

# Theme 2 - Innate Immune Responses to Viral Infections¶

The gene set is significantly associated with pathways related to innate immune activation, particularly through nucleic acid sensors that recognize foreign RNA and DNA, triggering immune responses and regulating inflammation. This suggests that the genes in this set may enhance antiviral defenses by modulating signaling pathways, such as ubiquitin-mediated regulation and RNA-dependent RNA polymerase activation, to effectively combat viral infections.

## Nucleic Acid Sensors in Innate Immune Activation Mechanisms¶

Key information

Key genes: Irf3, Dhx58, Tbk1, Irf7, Ikbke

Odds Ratio: 142.26

FDR: 0.000

Combined Score: 3409.64

The gene set is significantly associated with pathways involved in the activation of innate immunity, particularly through the role of nucleic acid sensors. These genes may play crucial roles in recognizing foreign RNA and DNA, thereby triggering immune responses and contributing to the regulation of inflammation.

StringDB references

- Distinct and Orchestrated Functions of RNA Sensors in Innate Immunity. (PMID:32668226)
- Signaling Through Nucleic Acid Sensors and Their Roles in Inflammatory Diseases. (PMID:33633744)
- Activation of Innate Immunity by Therapeutic Nucleic Acids. (PMID:34948156)

Ontology genesets

- Regulation Of Innate Immune Response (GO:0045088)
- Positive Regulation Of Innate Immune Response (GO:0045089)
- Negative Regulation Of Innate Immune Response (GO:0045824)
- Antiviral Innate Immune Response (GO:0140374)
- Regulation Of Adaptive Immune Response (GO:0002819)

Gene overlap matrix

Gene overlap matrix

## Ubiquitin-mediated regulation of antiviral signaling pathways¶

Key information

Key genes: Trim25, Dhx58, Ifih1, Rnf125, Isg15

Odds Ratio: 171.96

FDR: 0.000

Combined Score: 5642.60

The gene set is significantly associated with pathways involved in ubiquitin-mediated regulation of antiviral signaling, highlighting its role in modulating innate immune responses against viral infections. These genes may function by influencing the activity of ubiquitin ligases that regulate RIG-I-like receptor pathways, thereby enhancing or attenuating antiviral signaling mechanisms.

StringDB references

- TRIMmunity: the roles of the TRIM E3-ubiquitin ligase family in innate antiviral immunity. (PMID:24333484)
- Ubiquitination in the antiviral immune response. (PMID:25753787)
- Ubiquitin in the activation and attenuation of innate antiviral immunity. (PMID:26712804)
- Regulation of Cellular Antiviral Signaling by Modifications of Ubiquitin and Ubiquitin-like Molecules. (PMID:29503737)
- Recent Advances and Contradictions in the Study of the Individual Roles of Ubiquitin Ligases That Regulate RIG-I-Like Receptor-Mediated Antiviral Innate Immune Responses. (PMID:32670286)

Ontology genesets

- Ubiquitin Protein Ligase Activity (GO:0061630)
- Ubiquitin-Like Protein Ligase Activity (GO:0061659)
- Ubiquitin-Like Protein Transferase Activity (GO:0019787)
- Ubiquitin-Protein Transferase Activity (GO:0004842)
- Protein Ubiquitination (GO:0016567)

Gene overlap matrix

Gene overlap matrix

## RdRP Activation in Innate Antiviral Immune Responses¶

Key information

Key genes: Ifih1, Ifit1, Dhx58, Pycard, Nlrc4

Odds Ratio: 143.20

FDR: 0.000

Combined Score: 2889.57

The gene set is significantly associated with pathways involved in the activation of innate antiviral immune responses, highlighting its role in enhancing the body’s defense mechanisms against viral infections. These genes may function through RNA-dependent RNA polymerase (RdRP) pathways, contributing to the stable activation of innate immunity and potentially regulating antiviral responses.

StringDB references

- Activation of innate immune response (GO:0002218)
- Antiviral innate immune response (GO:0140374)
- Antiviral Protection via RdRP-Mediated Stable Activation of Innate Immunity. (PMID:26633895)
- RNA regulatory mechanisms that control antiviral innate immunity. (PMID:34405416)

Ontology genesets

- Antiviral Innate Immune Response (GO:0140374)
- Negative Regulation Of Innate Immune Response (GO:0045824)
- Innate Immune Response-Activating Signaling Pathway (GO:0002758)
- Negative Regulation Of Viral Process (GO:0048525)
- Negative Regulation Of Viral Genome Replication (GO:0045071)

Gene overlap matrix

Gene overlap matrix

## Regulation of bacterial defense responses to viral infections¶

Key information

Key genes: Ddx58, Dtx3l, Parp9, Stat1, Dhx58

Odds Ratio: 197.66

FDR: 0.000

Combined Score: 2618.01

The gene set is significantly associated with the regulation of bacterial defense responses to viral infections, highlighting its involvement in critical biological processes that enhance the host’s ability to combat viral threats. These genes may play a crucial role in modulating the immune response, particularly through pathways that positively regulate the defense mechanisms against viral infections in bacterial systems.

StringDB references

- Positive regulation of defense response to virus by host (GO:0002230)
- Regulation of defense response to virus (GO:0050688)

Ontology genesets

- Positive Regulation Of Defense Response To Virus By Host (GO:0002230)
- Regulation Of Defense Response To Virus By Host (GO:0050691)
- Antiviral Innate Immune Response (GO:0140374)
- Regulation Of Innate Immune Response (GO:0045088)
- Defense Response To Virus (GO:0051607)

Gene overlap matrix

Gene overlap matrix


← Theme 1 - Inflammation and Cell Proliferation Pathways

Theme 3 - Insulin and Cardiomyopathy Pathways in Disease  →

Page contents

- Theme 2 - Innate Immune Responses to Viral Infections
  - Nucleic Acid Sensors in Innate Immune Activation Mechanisms
  - Ubiquitin-mediated regulation of antiviral signaling pathways
  - RdRP Activation in Innate Antiviral Immune Responses
  - Regulation of bacterial defense responses to viral infections

- Contact
- Developers
