# Supplementary material for: GeneInsight: Condensing gene set knowledge via language models
Source: PLoS Comput Biol. 2026 Aug 5;22(8):e1014500. doi: 10.1371/journal.pcbi.1014500 (PMC13440848; doi:10.1371/journal.pcbi.1014500)
Supplement: Supplementary file 4 [file pcbi.1014500.s002.zip › S2 Data/sphinx_builds.zip!/sphinx_builds/html_build_geneset2/build/html/cluster_5.html]

Theme 6 - Antigen Presenting Cell Morphology and Function — GeneInsight documentation


Skip to content

GeneInsight


menu

Major themes:

- Theme 1 - Inflammation and Cell Proliferation Pathways
- Theme 2 - Innate Immune Responses to Viral Infections
- Theme 3 - Insulin and Cardiomyopathy Pathways in Disease
- Theme 4 - Transcription Regulation and Metal Complex Assembly
- Theme 5 - Immune Response and Macrophage Activation
- Theme 6 - Antigen Presenting Cell Morphology and Function
- Theme 7 - Regulation of TLR and Interferon Signaling
- Theme 8 - Organic Nitrogen Compound Metabolism
- Theme 9 - Innate Immunity in Viral Infections
- Theme 10 - CD15 Neutrophils in Colorectal Cancer

Metadata:

- Embedding map
- Summary statistics
- Data Files
- Downloads


Toggle menu contents

1. Docs
2. Theme 6 - Antigen Presenting Cell Morphology and Function

JavaScript is required to toggle light/dark mode..


 Light mode
 Dark mode


Edit on GitHub


View source

---

# Theme 6 - Antigen Presenting Cell Morphology and Function¶

The gene set is significantly associated with pathways related to the abnormal morphology and physiology of antigen presenting cells, indicating a disruption in immune system function. This suggests that the genes within this set may play a critical role in modulating the structure and function of antigen presenting cells, potentially impacting their ability to initiate and regulate immune responses. Additionally, the gene set is linked to pathways concerning dendritic cell morphology and differentiation, highlighting its involvement in the regulation of immune responses and the potential influence on how these cells present antigens and activate T cells.

## Abnormal Morphology and Physiology of Antigen Presenting Cells¶

Key information

Key genes: Stat3, Ifngr1, Ifnar1, Stat2, Irf1

Odds Ratio: 93.91

FDR: 0.000

Combined Score: 2046.65

The gene set is significantly associated with pathways related to the abnormal morphology and physiology of antigen presenting cells, indicating a disruption in immune system function. This suggests that the genes within this set may play a critical role in modulating the structure and function of antigen presenting cells, potentially impacting their ability to initiate and regulate immune responses.

StringDB references

- Abnormal professional antigen presenting cell morphology (MP:0008195)

Ontology genesets

- Cytoplasmic Side Of Plasma Membrane (GO:0009898)
- Extrinsic Component Of Cytoplasmic Side Of Plasma Membrane (GO:0031234)
- Cellular Response To Virus (GO:0098586)
- Dendritic Cell Migration (GO:0036336)
- Regulation Of Inflammatory Response To Antigenic Stimulus (GO:0002861)

Gene overlap matrix

Gene overlap matrix

## Dendritic Cell Morphology and Differentiation Mechanisms¶

Key information

Key genes: Lyn, Irf7, Tlr2, Tlr9, Traf6

Odds Ratio: 111.70

FDR: 0.000

Combined Score: 1316.80

The gene set is significantly associated with pathways related to dendritic cell morphology and differentiation, indicating its involvement in the regulation of immune responses. These genes may play critical roles in shaping dendritic cell function and structure, potentially influencing how these cells present antigens and activate T cells.

StringDB references

- Dendritic cell (BTO:0002042)
- Dendritic cell differentiation (GO:0097028)
- Abnormal dendritic cell morphology (MP:0005461)

Ontology genesets

- Dendritic Cell Migration (GO:0036336)
- Dendritic Cell Chemotaxis (GO:0002407)
- Regulation Of Lymphocyte Differentiation (GO:0045619)
- Regulation Of Myeloid Cell Differentiation (GO:0045637)
- Regulation Of B Cell Differentiation (GO:0045577)

Gene overlap matrix

Gene overlap matrix


← Theme 5 - Immune Response and Macrophage Activation

Theme 7 - Regulation of TLR and Interferon Signaling  →

Page contents

- Theme 6 - Antigen Presenting Cell Morphology and Function
  - Abnormal Morphology and Physiology of Antigen Presenting Cells
  - Dendritic Cell Morphology and Differentiation Mechanisms

- Contact
- Developers
