# Supplementary material for: GeneInsight: Condensing gene set knowledge via language models
Source: PLoS Comput Biol. 2026 Aug 5;22(8):e1014500. doi: 10.1371/journal.pcbi.1014500 (PMC13440848; doi:10.1371/journal.pcbi.1014500)
Supplement: Supplementary file 4 [file pcbi.1014500.s002.zip › S2 Data/sphinx_builds.zip!/sphinx_builds/html_build_geneset2/build/html/cluster_3.html]

Theme 4 - Transcription Regulation and Metal Complex Assembly — GeneInsight documentation


Skip to content

GeneInsight


menu

Major themes:

- Theme 1 - Inflammation and Cell Proliferation Pathways
- Theme 2 - Innate Immune Responses to Viral Infections
- Theme 3 - Insulin and Cardiomyopathy Pathways in Disease
- Theme 4 - Transcription Regulation and Metal Complex Assembly
- Theme 5 - Immune Response and Macrophage Activation
- Theme 6 - Antigen Presenting Cell Morphology and Function
- Theme 7 - Regulation of TLR and Interferon Signaling
- Theme 8 - Organic Nitrogen Compound Metabolism
- Theme 9 - Innate Immunity in Viral Infections
- Theme 10 - CD15 Neutrophils in Colorectal Cancer

Metadata:

- Embedding map
- Summary statistics
- Data Files
- Downloads


Toggle menu contents

1. Docs
2. Theme 4 - Transcription Regulation and Metal Complex Assembly

JavaScript is required to toggle light/dark mode..


 Light mode
 Dark mode


Edit on GitHub


View source

---

# Theme 4 - Transcription Regulation and Metal Complex Assembly¶

The gene set is significantly associated with pathways related to sequence-specific DNA-binding by RNA polymerase II transcription factors, indicating its involvement in the regulation of gene expression. These genes may play crucial roles in modulating transcriptional activity by binding to specific DNA sequences in the regulatory regions of target genes, thereby influencing various biological processes.

## Sequence-specific DNA-binding by RNA polymerase II transcription factors¶

Key information

Key genes: Stat2, Stat3, Irf9, Stat1, Irf1

Odds Ratio: 108.05

FDR: 0.000

Combined Score: 1658.37

The gene set is significantly associated with pathways related to sequence-specific DNA-binding by RNA polymerase II transcription factors, indicating its involvement in the regulation of gene expression. These genes may play crucial roles in modulating transcriptional activity by binding to specific DNA sequences in the regulatory regions of target genes, thereby influencing various biological processes.

StringDB references

- RNA polymerase II transcription regulatory region sequence-specific DNA binding (GO:0000977)
- RNA polymerase II cis-regulatory region sequence-specific DNA binding (GO:0000978)
- DNA-binding transcription factor activity, RNA polymerase II-specific (GO:0000981)
- RNA polymerase II-specific DNA-binding transcription factor binding (GO:0061629)

Ontology genesets

- RNA Polymerase II-specific DNA-binding Transcription Factor Binding (GO:0061629)
- RNA Polymerase II Transcription Regulatory Region Sequence-Specific DNA Binding (GO:0000977)
- RNA Polymerase II Cis-Regulatory Region Sequence-Specific DNA Binding (GO:0000978)
- Regulation Of Transcription By RNA Polymerase II (GO:0006357)
- DNA-binding Transcription Activator Activity, RNA Polymerase II-specific (GO:0001228)

Gene overlap matrix

Gene overlap matrix

## Regulation of Protein Binding in Metal-Containing Complex Assembly¶

Key information

Key genes: Calr, Tapbp, Tap2, Pdia3, B2m

Odds Ratio: 62.64

FDR: 0.000

Combined Score: 640.48

The gene set is significantly associated with the regulation of protein binding in metal-containing complex assembly, indicating its involvement in crucial biological processes related to protein interactions and complex formation. These genes may play a vital role in the assembly and regulation of metal-containing complexes, which are essential for various cellular functions, including enzymatic activity and structural integrity.

StringDB references

- Regulation of protein-containing complex assembly (GO:0043254)
- Protein-containing complex assembly (GO:0065003)

Ontology genesets

- Protein-Containing Complex Assembly (GO:0065003)
- Protein Complex Oligomerization (GO:0051259)
- protein-RNA Complex Assembly (GO:0022618)
- Peptide Antigen Assembly With MHC Protein Complex (GO:0002501)
- mRNA Binding (GO:0003729)

Gene overlap matrix

Gene overlap matrix


← Theme 3 - Insulin and Cardiomyopathy Pathways in Disease

Theme 5 - Immune Response and Macrophage Activation  →

Page contents

- Theme 4 - Transcription Regulation and Metal Complex Assembly
  - Sequence-specific DNA-binding by RNA polymerase II transcription factors
  - Regulation of Protein Binding in Metal-Containing Complex Assembly

- Contact
- Developers
