# Supplementary material for: GeneInsight: Condensing gene set knowledge via language models
Source: PLoS Comput Biol. 2026 Aug 5;22(8):e1014500. doi: 10.1371/journal.pcbi.1014500 (PMC13440848; doi:10.1371/journal.pcbi.1014500)
Supplement: Supplementary file 4 [file pcbi.1014500.s002.zip › S2 Data/sphinx_builds.zip!/sphinx_builds/html_build_geneset2/build/html/cluster_6.html]

Theme 7 - Regulation of TLR and Interferon Signaling — GeneInsight documentation


Skip to content

GeneInsight


menu

Major themes:

- Theme 1 - Inflammation and Cell Proliferation Pathways
- Theme 2 - Innate Immune Responses to Viral Infections
- Theme 3 - Insulin and Cardiomyopathy Pathways in Disease
- Theme 4 - Transcription Regulation and Metal Complex Assembly
- Theme 5 - Immune Response and Macrophage Activation
- Theme 6 - Antigen Presenting Cell Morphology and Function
- Theme 7 - Regulation of TLR and Interferon Signaling
- Theme 8 - Organic Nitrogen Compound Metabolism
- Theme 9 - Innate Immunity in Viral Infections
- Theme 10 - CD15 Neutrophils in Colorectal Cancer

Metadata:

- Embedding map
- Summary statistics
- Data Files
- Downloads


Toggle menu contents

1. Docs
2. Theme 7 - Regulation of TLR and Interferon Signaling

JavaScript is required to toggle light/dark mode..


 Light mode
 Dark mode


Edit on GitHub


View source

---

# Theme 7 - Regulation of TLR and Interferon Signaling¶

The gene set is significantly associated with various immune signaling pathways, including the regulation of type I interferon signaling and MyD88-dependent TLR signaling pathways, indicating its involvement in immune response and inflammation. This suggests that the genes in this set may modulate immune responses and inflammatory processes, potentially impacting conditions like viral infections and autoimmune diseases through their roles in TLR signaling and crosstalk with other pathways.

## Regulation of type I interferon signaling pathway dynamics¶

Key information

Key genes: Isg15, Irf7, Usp18, Stat2, Mavs

Odds Ratio: 478.71

FDR: 0.000

Combined Score: 9588.74

The gene set is significantly associated with the regulation of type I interferon signaling pathways, which are crucial for the immune response and antiviral defense. This suggests that the genes in this set may play a vital role in modulating immune responses, potentially influencing how cells respond to viral infections and inflammatory signals.

StringDB references

- Regulation of type I interferon-mediated signaling pathway (GO:0060338)

Ontology genesets

- Regulation Of Type I Interferon-Mediated Signaling Pathway (GO:0060338)
- Positive Regulation Of Type I Interferon-Mediated Signaling Pathway (GO:0060340)
- Type I Interferon-Mediated Signaling Pathway (GO:0060337)
- Negative Regulation Of Type I Interferon-Mediated Signaling Pathway (GO:0060339)
- Interferon-Mediated Signaling Pathway (GO:0140888)

Gene overlap matrix

Gene overlap matrix

## MyD88-Dependent TLR Signaling Pathway Regulation Mechanisms¶

Key information

Key genes: Irf7, Traf6, Tlr2, Irak1, Myd88

Odds Ratio: 95.26

FDR: 0.001

Combined Score: 740.26

The gene set is significantly associated with the MyD88-dependent TLR signaling pathway, which plays a crucial role in mediating immune responses and inflammation. These genes may contribute to the regulation of adaptive immune responses and inflammatory processes, potentially influencing conditions such as multiple sclerosis through their involvement in TLR-MyD88 signaling.

StringDB references

- Regulation of MyD88-dependent toll-like receptor signaling pathway (GO:0034124)
- Protein kinase D1 is essential for MyD88-dependent TLR signaling pathway. (PMID:19414785)
- Targeting the TLR9-MyD88 pathway in the regulation of adaptive immune responses. (PMID:20560798)
- Inflammatory Role of TLR-MyD88 Signaling in Multiple Sclerosis. (PMID:31998072)

Ontology genesets

- MyD88-dependent Toll-Like Receptor Signaling Pathway (GO:0002755)
- Positive Regulation Of Interleukin-8 Production (GO:0032757)
- Regulation Of Interleukin-8 Production (GO:0032677)
- Toll-Like Receptor 9 Signaling Pathway (GO:0034162)
- Regulation Of Adaptive Immune Response (GO:0002819)

Gene overlap matrix

Gene overlap matrix

## Integration of TLR Signaling Pathways and Crosstalk Mechanisms¶

Key information

Key genes: Irf1, Gbp2, Tlr2, Tlr4, Iigp1

Odds Ratio: 133.96

FDR: 0.000

Combined Score: 2664.79

The gene set is significantly associated with the integration of Toll-like receptor (TLR) signaling pathways, highlighting its involvement in key biological processes such as immune response and inflammation. These genes may play critical roles in modulating crosstalk between TLR and G protein-coupled receptor (GPCR) signaling, potentially influencing the strength and nature of immune responses.

StringDB references

- Regulation of TLR by endogenous ligand (MMU-5686938)
- TLR-signaling networks: an integration of adaptor molecules, kinases, and cross-talk. (PMID:20940366)
- Systems Biological Approaches Reveal Non-additive Responses and Multiple Crosstalk Mechanisms between TLR and GPCR Signaling. (PMID:23166526)

Ontology genesets

- Interferon-Mediated Signaling Pathway (GO:0140888)
- Type I Interferon-Mediated Signaling Pathway (GO:0060337)
- Positive Regulation Of Intracellular Signal Transduction (GO:1902533)
- Toll-Like Receptor Signaling Pathway (GO:0002224)
- Regulation Of Toll-Like Receptor Signaling Pathway (GO:0034121)

Gene overlap matrix

Gene overlap matrix


← Theme 6 - Antigen Presenting Cell Morphology and Function

Theme 8 - Organic Nitrogen Compound Metabolism  →

Page contents

- Theme 7 - Regulation of TLR and Interferon Signaling
  - Regulation of type I interferon signaling pathway dynamics
  - MyD88-Dependent TLR Signaling Pathway Regulation Mechanisms
  - Integration of TLR Signaling Pathways and Crosstalk Mechanisms

- Contact
- Developers
