# Supplementary material for: GeneInsight: Condensing gene set knowledge via language models
Source: PLoS Comput Biol. 2026 Aug 5;22(8):e1014500. doi: 10.1371/journal.pcbi.1014500 (PMC13440848; doi:10.1371/journal.pcbi.1014500)
Supplement: Supplementary file 4 [file pcbi.1014500.s002.zip › S2 Data/sphinx_builds.zip!/sphinx_builds/html_build_geneset2/build/html/cluster_0.html]

Theme 1 - Inflammation and Cell Proliferation Pathways — GeneInsight documentation


Skip to content

GeneInsight


menu

Major themes:

- Theme 1 - Inflammation and Cell Proliferation Pathways
- Theme 2 - Innate Immune Responses to Viral Infections
- Theme 3 - Insulin and Cardiomyopathy Pathways in Disease
- Theme 4 - Transcription Regulation and Metal Complex Assembly
- Theme 5 - Immune Response and Macrophage Activation
- Theme 6 - Antigen Presenting Cell Morphology and Function
- Theme 7 - Regulation of TLR and Interferon Signaling
- Theme 8 - Organic Nitrogen Compound Metabolism
- Theme 9 - Innate Immunity in Viral Infections
- Theme 10 - CD15 Neutrophils in Colorectal Cancer

Metadata:

- Embedding map
- Summary statistics
- Data Files
- Downloads


Toggle menu contents

1. Docs
2. Theme 1 - Inflammation and Cell Proliferation Pathways

JavaScript is required to toggle light/dark mode..


 Light mode
 Dark mode


Edit on GitHub


View source

---

# Theme 1 - Inflammation and Cell Proliferation Pathways¶

The gene set is significantly associated with pathways related to interleukin-6 mediated pro-inflammatory signaling, glial and epithelial cell proliferation, interleukin-27 signaling, and oxidative stress response. This suggests that the genes in this set may play crucial roles in regulating inflammation, promoting cell growth and migration, modulating immune responses, and enhancing cellular resilience against oxidative stress, particularly in the context of retinal health and tissue repair.

## Interleukin-6 Mediated Pro-Inflammatory Signaling in Neural Retina¶

Key information

Key genes: Il6, Il6ra, Stat3, Jak2, Il6st

Odds Ratio: 86.18

FDR: 0.001

Combined Score: 654.18

The gene set is significantly associated with interleukin-6 mediated pro-inflammatory signaling pathways, indicating a role in the regulation of inflammatory responses in the neural retina. This suggests that the genes within this set may contribute to the modulation of inflammation and immune responses, potentially influencing conditions related to retinal health and disease.

StringDB references

- interleukin-6-mediated signaling pathway (GO:0070102)
- Interleukin-6 signaling (MMU-1059683)
- Primate neural retina upregulates IL-6 and IL-10 in response to a herpes simplex vector suggesting the presence of a pro-anti-inflammatory axis. (PMID:27170050)

Ontology genesets

- Interleukin-6-Mediated Signaling Pathway (GO:0070102)
- Cellular Response To Interleukin-6 (GO:0071354)
- Regulation Of Cytokine Production Involved In Inflammatory Response (GO:1900015)
- Cellular Response To Cytokine Stimulus (GO:0071345)
- Cellular Response To Interleukin-9 (GO:0071355)

Gene overlap matrix

Gene overlap matrix

## Positive regulation of glial and epithelial cell proliferation¶

Key information

Key genes: Ccl5, Cxcl12, Il6, Ccl2, Ccr3

Odds Ratio: 106.47

FDR: 0.001

Combined Score: 848.55

The gene set is significantly associated with the positive regulation of glial and epithelial cell proliferation, indicating its involvement in promoting the growth and migration of these cell types. This suggests that the genes in this set may play crucial roles in tissue repair and regeneration processes, particularly in the nervous system and epithelial tissues, by enhancing cell proliferation and migration.

StringDB references

- Positive regulation of epithelial cell proliferation (GO:0050679)
- Positive regulation of glial cell proliferation (GO:0060252)
- Positive regulation of glial cell migration (GO:1903977)

Ontology genesets

- Positive Regulation Of Epithelial Cell Apoptotic Process (GO:1904037)
- Positive Regulation Of T Cell Proliferation (GO:0042102)
- Positive Regulation Of Smooth Muscle Cell Proliferation (GO:0048661)
- Positive Regulation Of Lymphocyte Proliferation (GO:0050671)
- Positive Regulation Of Signaling Receptor Activity (GO:2000273)

Gene overlap matrix

Gene overlap matrix

## Interleukin-27 Signaling Pathway in Immune Response Regulation¶

Key information

Key genes: Oasl2, Oas2, Stat1, Oasl1, Jak2

Odds Ratio: 285.53

FDR: 0.000

Combined Score: 4030.67

The gene set is significantly associated with the interleukin-27 signaling pathway, which plays a crucial role in regulating immune responses. This suggests that the genes within this set may be involved in modulating inflammation and immune cell activity, potentially influencing the body’s defense mechanisms against pathogens.

StringDB references

- interleukin-27-mediated signaling pathway (GO:0070106)
- Interleukin-27 signaling (MMU-9020956)

Ontology genesets

- Interleukin-27-Mediated Signaling Pathway (GO:0070106)
- Interleukin-9-Mediated Signaling Pathway (GO:0038113)
- Cellular Response To Interleukin-9 (GO:0071355)
- Cellular Response To Interleukin-6 (GO:0071354)
- Interleukin-6-Mediated Signaling Pathway (GO:0070102)

Gene overlap matrix

Gene overlap matrix

## Molecular mechanisms of oxidative stress response pathways¶

Key information

Key genes: Jun, Il6, Jak2, Nfkb1, Stat3

Odds Ratio: 120.67

FDR: 0.001

Combined Score: 988.44

The gene set is significantly associated with oxidative stress response pathways, indicating its involvement in critical biological processes that help cells manage and mitigate damage caused by reactive oxygen species. These genes may play essential roles in activating protective mechanisms, such as antioxidant production and cellular repair processes, thereby enhancing cellular resilience against oxidative stress.

StringDB references

- Response to oxidative stress (GO:0006979)
- Cellular response to oxidative stress (GO:0034599)
- Oxidative stress (MP:0003674)
- Oxidative stress response (WP412)

Ontology genesets

- Regulation Of Oxidative Stress-Induced Intrinsic Apoptotic Signaling Pathway (GO:1902175)
- Cellular Response To Superoxide (GO:0071451)
- Response To Reactive Oxygen Species (GO:0000302)
- Superoxide Metabolic Process (GO:0006801)
- Response To Hydrogen Peroxide (GO:0042542)

Gene overlap matrix

Gene overlap matrix


← GeneInsight Topic Descriptions

Theme 2 - Innate Immune Responses to Viral Infections  →

Page contents

- Theme 1 - Inflammation and Cell Proliferation Pathways
  - Interleukin-6 Mediated Pro-Inflammatory Signaling in Neural Retina
  - Positive regulation of glial and epithelial cell proliferation
  - Interleukin-27 Signaling Pathway in Immune Response Regulation
  - Molecular mechanisms of oxidative stress response pathways

- Contact
- Developers
