# Supplementary material for: GeneInsight: Condensing gene set knowledge via language models
Source: PLoS Comput Biol. 2026 Aug 5;22(8):e1014500. doi: 10.1371/journal.pcbi.1014500 (PMC13440848; doi:10.1371/journal.pcbi.1014500)
Supplement: Supplementary file 4 [file pcbi.1014500.s002.zip › S2 Data/sphinx_builds.zip!/sphinx_builds/html_build_geneset2/build/html/cluster_8.html]

Theme 9 - Innate Immunity in Viral Infections — GeneInsight documentation


Skip to content

GeneInsight


menu

Major themes:

- Theme 1 - Inflammation and Cell Proliferation Pathways
- Theme 2 - Innate Immune Responses to Viral Infections
- Theme 3 - Insulin and Cardiomyopathy Pathways in Disease
- Theme 4 - Transcription Regulation and Metal Complex Assembly
- Theme 5 - Immune Response and Macrophage Activation
- Theme 6 - Antigen Presenting Cell Morphology and Function
- Theme 7 - Regulation of TLR and Interferon Signaling
- Theme 8 - Organic Nitrogen Compound Metabolism
- Theme 9 - Innate Immunity in Viral Infections
- Theme 10 - CD15 Neutrophils in Colorectal Cancer

Metadata:

- Embedding map
- Summary statistics
- Data Files
- Downloads


Toggle menu contents

1. Docs
2. Theme 9 - Innate Immunity in Viral Infections

JavaScript is required to toggle light/dark mode..


 Light mode
 Dark mode


Edit on GitHub


View source

---

# Theme 9 - Innate Immunity in Viral Infections¶

The gene set is significantly associated with pathways involved in innate immune mechanisms, particularly in the context of West Nile virus infection, as highlighted by multiple studies. These genes may play crucial roles in the cell-intrinsic control of viral replication and spread, suggesting that they are key players in the host’s defense against viral pathogens.

## Innate Immune Mechanisms in West Nile Virus Infection¶

Key information

Key genes: Eif2ak2, Ifit1, Rsad2, Irf7, Rnasel

Odds Ratio: 343.07

FDR: 0.000

Combined Score: 11204.43

The gene set is significantly associated with pathways involved in innate immune mechanisms, particularly in the context of West Nile virus infection, as highlighted by multiple studies. These genes may play crucial roles in the cell-intrinsic control of viral replication and spread, suggesting that they are key players in the host’s defense against viral pathogens.

StringDB references

- Cell-intrinsic innate immune control of West Nile virus infection. (PMID:22726607)
- The innate immune playbook for restricting West Nile virus infection. (PMID:24178712)
- New insights into innate immune restriction of West Nile virus infection. (PMID:25554924)

Ontology genesets

- Antiviral Innate Immune Response (GO:0140374)
- Regulation Of Immune Response (GO:0050776)
- Positive Regulation Of Defense Response To Virus By Host (GO:0002230)
- Regulation Of Defense Response To Virus By Host (GO:0050691)
- Negative Regulation Of Viral Process (GO:0048525)

Gene overlap matrix

Gene overlap matrix


← Theme 8 - Organic Nitrogen Compound Metabolism

Theme 10 - CD15 Neutrophils in Colorectal Cancer  →

Page contents

- Theme 9 - Innate Immunity in Viral Infections
  - Innate Immune Mechanisms in West Nile Virus Infection

- Contact
- Developers
