# Supplementary material for: GeneInsight: Condensing gene set knowledge via language models
Source: PLoS Comput Biol. 2026 Aug 5;22(8):e1014500. doi: 10.1371/journal.pcbi.1014500 (PMC13440848; doi:10.1371/journal.pcbi.1014500)
Supplement: Supplementary file 4 [file pcbi.1014500.s002.zip › S2 Data/sphinx_builds.zip!/sphinx_builds/html_build_geneset2/build/html/cluster_4.html]

Theme 5 - Immune Response and Macrophage Activation — GeneInsight documentation


Skip to content

GeneInsight


menu

Major themes:

- Theme 1 - Inflammation and Cell Proliferation Pathways
- Theme 2 - Innate Immune Responses to Viral Infections
- Theme 3 - Insulin and Cardiomyopathy Pathways in Disease
- Theme 4 - Transcription Regulation and Metal Complex Assembly
- Theme 5 - Immune Response and Macrophage Activation
- Theme 6 - Antigen Presenting Cell Morphology and Function
- Theme 7 - Regulation of TLR and Interferon Signaling
- Theme 8 - Organic Nitrogen Compound Metabolism
- Theme 9 - Innate Immunity in Viral Infections
- Theme 10 - CD15 Neutrophils in Colorectal Cancer

Metadata:

- Embedding map
- Summary statistics
- Data Files
- Downloads


Toggle menu contents

1. Docs
2. Theme 5 - Immune Response and Macrophage Activation

JavaScript is required to toggle light/dark mode..


 Light mode
 Dark mode


Edit on GitHub


View source

---

# Theme 5 - Immune Response and Macrophage Activation¶

The gene set is significantly associated with pathways involved in macrophage differentiation, myeloid leukocyte regulation, immune cell interactions in the tumor microenvironment, and interleukin-1 signaling, indicating its critical role in modulating immune responses and inflammation. These genes may influence the activation states of macrophages, the differentiation and activity of myeloid cells, and the regulation of immune interactions in cancer, suggesting their potential as targets for enhancing immunotherapy and managing inflammatory diseases.

## Differential Gene Expression in Classically vs. Alternatively Activated Macrophages¶

Key information

Key genes: Irf1, Ripk1, Jun, Oasl1, Gbp2

Odds Ratio: 197.08

FDR: 0.000

Combined Score: 3427.54

The gene set is significantly associated with the positive regulation of macrophage differentiation, indicating its involvement in the modulation of immune responses and inflammation. These genes may play critical roles in the transcriptional regulation of macrophage activation states, particularly in how classically and alternatively activated macrophages respond to stimuli such as LPS.

StringDB references

- Positive regulation of macrophage differentiation (GO:0045651)
- Jmjd3 contributes to the control of gene expression in LPS-activated macrophages. (PMID:19779457)
- Redefining the transcriptional regulatory dynamics of classically and alternatively activated macrophages by deepCAGE transcriptomics. (PMID:26117544)
- High-Resolution Mapping and Dynamics of the Transcriptome, Transcription Factors, and Transcription Co-Factor Networks in Classically and Alternatively Activated Macrophages. (PMID:29403501)

Ontology genesets

- Regulation Of Macrophage Differentiation (GO:0045649)
- Positive Regulation Of Macrophage Differentiation (GO:0045651)
- Dendritic Cell Differentiation (GO:0097028)
- Myeloid Dendritic Cell Differentiation (GO:0043011)
- Regulation Of Innate Immune Response (GO:0045088)

Gene overlap matrix

Gene overlap matrix

## Regulation of Myeloid Leukocyte Differentiation and Migration in Immunity¶

Key information

Key genes: Fcgr1, Lyn, Fcer1g, Syk, Fcgr3

Odds Ratio: 78.69

FDR: 0.001

Combined Score: 584.27

The gene set is significantly associated with the regulation of myeloid leukocyte differentiation and migration, highlighting its involvement in immune responses. These genes may play crucial roles in modulating the differentiation and activity of myeloid cells, which are essential for effective immune defense and inflammation.

StringDB references

- Myeloid leukocyte mediated immunity (GO:0002444)
- Positive regulation of myeloid leukocyte differentiation (GO:0002763)
- Regulation of myeloid leukocyte mediated immunity (GO:0002886)
- Negative regulation of myeloid leukocyte mediated immunity (GO:0002887)

Ontology genesets

- Regulation Of Myeloid Leukocyte Mediated Immunity (GO:0002886)
- Positive Regulation Of Myeloid Leukocyte Differentiation (GO:0002763)
- Regulation Of Myeloid Cell Differentiation (GO:0045637)
- Regulation Of Leukocyte Degranulation (GO:0043300)
- Positive Regulation Of Leukocyte Cell-Cell Adhesion (GO:1903039)

Gene overlap matrix

Gene overlap matrix

## Characterization of immune cell interactions in tumor microenvironment¶

Key information

Key genes: Cd274, Havcr2, Ccl5, Cxcl10, Cxcr3

Odds Ratio: 52.40

FDR: 0.000

Combined Score: 509.96

The gene set is significantly associated with pathways related to immune cell interactions in the tumor microenvironment, highlighting its involvement in immune responses to tumor cells and the regulation of these interactions. This suggests that the genes in this set may play crucial roles in modulating immune responses and could be targeted for enhancing the efficacy of precision immunotherapy in cancer treatment.

StringDB references

- Immune response to tumor cell (GO:0002418)
- Regulation of immune response to tumor cell (GO:0002837)
- Chemokine-Directed Tumor Microenvironment Modulation in Cancer Immunotherapy. (PMID:34575965)
- Exploring Tumor Immune Microenvironment and Its Associations With Molecular Characteristics in Melanoma. (PMID:35530341)
- Modelling the tumor immune microenvironment for precision immunotherapy. (PMID:35782339)

Ontology genesets

- Cellular Response To Chemokine (GO:1990869)
- Cellular Response To Tumor Necrosis Factor (GO:0071356)
- Cellular Response To Interleukin-1 (GO:0071347)
- Regulation Of Immune Response (GO:0050776)
- Lymphocyte Chemotaxis (GO:0048247)

Gene overlap matrix

Gene overlap matrix

## Role of IPS-1 in Interleukin-1 Mediated Cytokine Production¶

Key information

Key genes: Psmb8, Psmb9, Psmb10, Psma4, Psmb1

Odds Ratio: 133.96

FDR: 0.000

Combined Score: 2664.79

The gene set is significantly associated with interleukin-1 signaling pathways, which are crucial for mediating inflammatory responses and cytokine production. This suggests that the genes in this set may play a vital role in regulating immune responses, potentially influencing the severity and outcome of inflammatory diseases.

StringDB references

- Interleukin-1 signaling (MMU-9020702)

Ontology genesets

- Interleukin-1-Mediated Signaling Pathway (GO:0070498)
- Cellular Response To Interleukin-1 (GO:0071347)
- Cytokine-Mediated Signaling Pathway (GO:0019221)
- Positive Regulation Of I-kappaB kinase/NF-kappaB Signaling (GO:0043123)
- Regulation Of I-kappaB kinase/NF-kappaB Signaling (GO:0043122)

Gene overlap matrix

Gene overlap matrix


← Theme 4 - Transcription Regulation and Metal Complex Assembly

Theme 6 - Antigen Presenting Cell Morphology and Function  →

Page contents

- Theme 5 - Immune Response and Macrophage Activation
  - Differential Gene Expression in Classically vs. Alternatively Activated Macrophages
  - Regulation of Myeloid Leukocyte Differentiation and Migration in Immunity
  - Characterization of immune cell interactions in tumor microenvironment
  - Role of IPS-1 in Interleukin-1 Mediated Cytokine Production

- Contact
- Developers
