# Supplementary material for: GeneInsight: Condensing gene set knowledge via language models
Source: PLoS Comput Biol. 2026 Aug 5;22(8):e1014500. doi: 10.1371/journal.pcbi.1014500 (PMC13440848; doi:10.1371/journal.pcbi.1014500)
Supplement: Supplementary file 5 [file pcbi.1014500.s003.zip › S3 Data/sphinx_builds.zip!/sphinx_builds/html_build_geneset1/build/html/cluster_7.html]

Theme 8 - Biomarkers in Triple-Negative Breast Cancer — GeneInsight documentation


Skip to content

GeneInsight


menu

Major themes:

- Theme 1 - Lipid Transport and Protein Interactions
- Theme 2 - Leukocyte Signaling and Immune Regulation
- Theme 3 - Lymphocyte Activation and Proliferation Regulation
- Theme 4 - Autoimmune Disease Pathogenesis and Genetics
- Theme 5 - Cancer Pathways and Immune Response
- Theme 6 - Nitrogen Metabolism Regulation in Biological Processes
- Theme 7 - Aminopterin in Canine Atopic Dermatitis Treatment
- Theme 8 - Biomarkers in Triple-Negative Breast Cancer
- Theme 9 - Immune Response Regulation in Coral Bleaching
- Theme 10 - Hepatitis Viruses in Pregnancy and Cancer

Metadata:

- Embedding map
- Summary statistics
- Data Files
- Downloads


Toggle menu contents

1. Docs
2. Theme 8 - Biomarkers in Triple-Negative Breast Cancer

JavaScript is required to toggle light/dark mode..


 Light mode
 Dark mode


Edit on GitHub


View source

---

# Theme 8 - Biomarkers in Triple-Negative Breast Cancer¶

The gene set associated with oncogenic biomarkers in triple-negative breast cancer is significantly enriched in pathways related to tumor suppression, immune response, and therapeutic resistance. These genes may play crucial roles in modulating cancer cell behavior and treatment responses, particularly through interactions with pathways involving TP53, PIK3CA, and PP2A.

## Oncogenic Biomarkers in Triple-Negative Breast Cancer¶

Key information

Key genes: PPP2R5A, PPP2R1A, PPP2R2A, LIN37, LIN9

Odds Ratio: 30.20

FDR: 0.001

Combined Score: 315.13

The gene set associated with oncogenic biomarkers in triple-negative breast cancer is significantly enriched in pathways related to tumor suppression, immune response, and therapeutic resistance. These genes may play crucial roles in modulating cancer cell behavior and treatment responses, particularly through interactions with pathways involving TP53, PIK3CA, and PP2A.

StringDB references

- Mitotic Vulnerability in Triple-Negative Breast Cancer Associated with LIN9 Is Targetable with BET Inhibitors. (PMID:28807940)
- PP2A as the Main Node of Therapeutic Strategies and Resistance Reversal in Triple-Negative Breast Cancer. (PMID:29261144)
- Gene expression profiling identified TP53`(Mut) <Unknown identifier format: Mut>`\_PIK3CA`(Wild) <Unknown identifier format: Wild>`\_ as a potential biomarker for patients with triple-negative breast cancer treated with immune checkpoint inhibitors. (PMID:32218835)
- CDC25B partners with PP2A to induce AMPK activation and tumor suppression in triple negative breast cancer. (PMID:33385163)
- Evaluation of Oncogene NUP37 as a Potential Novel Biomarker in Breast Cancer. (PMID:34386417)

Ontology genesets

- Regulation Of T Cell Activation (GO:0050863)
- Dephosphorylation (GO:0016311)
- T Cell Activation (GO:0042110)
- Protein Dephosphorylation (GO:0006470)
- Protein Phosphatase Regulator Activity (GO:0019888)

Gene overlap matrix

Gene overlap matrix

## Prognostic Biomarkers in Triple-Negative Breast Cancer Models¶

Key information

Key genes: TTC19, UQCRFS1, UQCC1, BCS1L, UQCC2

Odds Ratio: 39.58

FDR: 0.000

Combined Score: 553.73

The gene set associated with triple-negative breast cancer (TNBC) shows significant enrichment in pathways related to immune response and cell cycle regulation, indicating its involvement in critical biological processes that influence tumor progression and treatment response. These genes, particularly TP53 and PIK3CA, may play pivotal roles in modulating therapeutic resistance and vulnerability, suggesting that targeting these pathways could enhance treatment efficacy for patients with TNBC.

StringDB references

- CYC1 Predicts Poor Prognosis in Patients with Breast Cancer. (PMID:27239088)
- Mitotic Vulnerability in Triple-Negative Breast Cancer Associated with LIN9 Is Targetable with BET Inhibitors. (PMID:28807940)
- PP2A as the Main Node of Therapeutic Strategies and Resistance Reversal in Triple-Negative Breast Cancer. (PMID:29261144)
- Gene expression profiling identified TP53`(Mut) <Unknown identifier format: Mut>`\_PIK3CA`(Wild) <Unknown identifier format: Wild>`\_ as a potential biomarker for patients with triple-negative breast cancer treated with immune checkpoint inhibitors. (PMID:32218835)
- Computational assessment of MCM2 transcriptional expression and identification of the prognostic biomarker for human breast cancer. (PMID:33024849)

Ontology genesets

- Mitochondrial Respiratory Chain Complex III Assembly (GO:0034551)
- Protein Dephosphorylation (GO:0006470)
- Double-Strand Break Repair Via Break-Induced Replication (GO:0000727)
- Dephosphorylation (GO:0016311)
- T Cell Activation (GO:0042110)

Gene overlap matrix

Gene overlap matrix


← Theme 7 - Aminopterin in Canine Atopic Dermatitis Treatment

Theme 9 - Immune Response Regulation in Coral Bleaching  →

Page contents

- Theme 8 - Biomarkers in Triple-Negative Breast Cancer
  - Oncogenic Biomarkers in Triple-Negative Breast Cancer
  - Prognostic Biomarkers in Triple-Negative Breast Cancer Models

- Contact
- Developers
