# Supplementary material for: GeneInsight: Condensing gene set knowledge via language models
Source: PLoS Comput Biol. 2026 Aug 5;22(8):e1014500. doi: 10.1371/journal.pcbi.1014500 (PMC13440848; doi:10.1371/journal.pcbi.1014500)
Supplement: Supplementary file 5 [file pcbi.1014500.s003.zip › S3 Data/sphinx_builds.zip!/sphinx_builds/html_build_geneset1/build/html/cluster_9.html]

Theme 10 - Hepatitis Viruses in Pregnancy and Cancer — GeneInsight documentation


Skip to content

GeneInsight


menu

Major themes:

- Theme 1 - Lipid Transport and Protein Interactions
- Theme 2 - Leukocyte Signaling and Immune Regulation
- Theme 3 - Lymphocyte Activation and Proliferation Regulation
- Theme 4 - Autoimmune Disease Pathogenesis and Genetics
- Theme 5 - Cancer Pathways and Immune Response
- Theme 6 - Nitrogen Metabolism Regulation in Biological Processes
- Theme 7 - Aminopterin in Canine Atopic Dermatitis Treatment
- Theme 8 - Biomarkers in Triple-Negative Breast Cancer
- Theme 9 - Immune Response Regulation in Coral Bleaching
- Theme 10 - Hepatitis Viruses in Pregnancy and Cancer

Metadata:

- Embedding map
- Summary statistics
- Data Files
- Downloads


Toggle menu contents

1. Docs
2. Theme 10 - Hepatitis Viruses in Pregnancy and Cancer

JavaScript is required to toggle light/dark mode..


 Light mode
 Dark mode


Edit on GitHub


View source

---

# Theme 10 - Hepatitis Viruses in Pregnancy and Cancer¶

The gene set is significantly associated with pathways related to hepatitis virus infections and their impact on pregnant women, highlighting enriched biological processes such as immune response alterations and liver disease progression. These genes may play crucial roles in modulating the immune microenvironment and influencing the development of hepatocellular carcinoma (HCC) in the context of high viral loads, suggesting potential mechanisms of disease susceptibility and progression.

## Impact of Hepatitis Viruses on Pregnant Women and HCC¶

Key information

Key genes: GZMH, NKG7, GZMK, FGFBP2, KLRD1

Odds Ratio: 28.56

FDR: 0.001

Combined Score: 292.53

The gene set is significantly associated with pathways related to hepatitis virus infections and their impact on pregnant women, highlighting enriched biological processes such as immune response alterations and liver disease progression. These genes may play crucial roles in modulating the immune microenvironment and influencing the development of hepatocellular carcinoma (HCC) in the context of high viral loads, suggesting potential mechanisms of disease susceptibility and progression.

StringDB references

- Alteration of the Immune Microenvironment in HBsAg and HBeAg Dual-Positive Pregnant Women Presenting a High HBV Viral Load. (PMID:34764667)
- Molecular Signature and Immune Landscape of HCV-Associated Hepatocellular Carcinoma (HCC): Differences and Similarities with HBV-HCC. (PMID:34849372)
- Hepatitis C (hsa05160)

Ontology genesets

- Positive Regulation Of Myeloid Leukocyte Differentiation (GO:0002763)
- Positive Regulation Of Macrophage Migration (GO:1905523)
- Regulation Of Macrophage Proliferation (GO:0120040)
- Positive Regulation Of Defense Response (GO:0031349)
- Regulation Of Macrophage Differentiation (GO:0045649)

Gene overlap matrix

Gene overlap matrix


← Theme 9 - Immune Response Regulation in Coral Bleaching

Embedding map  →

Page contents

- Theme 10 - Hepatitis Viruses in Pregnancy and Cancer
  - Impact of Hepatitis Viruses on Pregnant Women and HCC

- Contact
- Developers
