# Supplementary material for: GeneInsight: Condensing gene set knowledge via language models
Source: PLoS Comput Biol. 2026 Aug 5;22(8):e1014500. doi: 10.1371/journal.pcbi.1014500 (PMC13440848; doi:10.1371/journal.pcbi.1014500)
Supplement: Supplementary file 5 [file pcbi.1014500.s003.zip › S3 Data/sphinx_builds.zip!/sphinx_builds/html_build_geneset1/build/html/download.html]

Downloads — GeneInsight documentation


Skip to content

GeneInsight


menu

Major themes:

- Theme 1 - Lipid Transport and Protein Interactions
- Theme 2 - Leukocyte Signaling and Immune Regulation
- Theme 3 - Lymphocyte Activation and Proliferation Regulation
- Theme 4 - Autoimmune Disease Pathogenesis and Genetics
- Theme 5 - Cancer Pathways and Immune Response
- Theme 6 - Nitrogen Metabolism Regulation in Biological Processes
- Theme 7 - Aminopterin in Canine Atopic Dermatitis Treatment
- Theme 8 - Biomarkers in Triple-Negative Breast Cancer
- Theme 9 - Immune Response Regulation in Coral Bleaching
- Theme 10 - Hepatitis Viruses in Pregnancy and Cancer

Metadata:

- Embedding map
- Summary statistics
- Data Files
- Downloads


Toggle menu contents

1. Docs
2. Downloads

JavaScript is required to toggle light/dark mode..


 Light mode
 Dark mode


Edit on GitHub


View source

---

# Downloads¶

Select the themes you want to download by checking the boxes. Click “Download Selected” to get a ZIP file of the selected themes.

0

Themes selected

Theme 1 - Lipid Transport and Protein Interactions

Theme 2 - Leukocyte Signaling and Immune Regulation

Theme 3 - Lymphocyte Activation and Proliferation Regulation

Theme 4 - Autoimmune Disease Pathogenesis and Genetics

Theme 5 - Cancer Pathways and Immune Response

Theme 6 - Nitrogen Metabolism Regulation in Biological Processes

Theme 7 - Aminopterin in Canine Atopic Dermatitis Treatment

Theme 8 - Biomarkers in Triple-Negative Breast Cancer

Theme 9 - Immune Response Regulation in Coral Bleaching

Theme 10 - Hepatitis Viruses in Pregnancy and Cancer

Select All Themes
Download Selected


← Embedding map

- Contact
- Developers
