# Supplementary material for: GeneInsight: Condensing gene set knowledge via language models
Source: PLoS Comput Biol. 2026 Aug 5;22(8):e1014500. doi: 10.1371/journal.pcbi.1014500 (PMC13440848; doi:10.1371/journal.pcbi.1014500)
Supplement: Supplementary file 5 [file pcbi.1014500.s003.zip › S3 Data/sphinx_builds.zip!/sphinx_builds/html_build_geneset1/build/html/cluster_2.html]

Theme 3 - Lymphocyte Activation and Proliferation Regulation — GeneInsight documentation


Skip to content

GeneInsight


menu

Major themes:

- Theme 1 - Lipid Transport and Protein Interactions
- Theme 2 - Leukocyte Signaling and Immune Regulation
- Theme 3 - Lymphocyte Activation and Proliferation Regulation
- Theme 4 - Autoimmune Disease Pathogenesis and Genetics
- Theme 5 - Cancer Pathways and Immune Response
- Theme 6 - Nitrogen Metabolism Regulation in Biological Processes
- Theme 7 - Aminopterin in Canine Atopic Dermatitis Treatment
- Theme 8 - Biomarkers in Triple-Negative Breast Cancer
- Theme 9 - Immune Response Regulation in Coral Bleaching
- Theme 10 - Hepatitis Viruses in Pregnancy and Cancer

Metadata:

- Embedding map
- Summary statistics
- Data Files
- Downloads


Toggle menu contents

1. Docs
2. Theme 3 - Lymphocyte Activation and Proliferation Regulation

JavaScript is required to toggle light/dark mode..


 Light mode
 Dark mode


Edit on GitHub


View source

---

# Theme 3 - Lymphocyte Activation and Proliferation Regulation¶

The gene set is significantly associated with the regulation of B-lymphocyte and T-lymphocyte activation and proliferation, highlighting its critical role in immune responses and lymphocyte development. This suggests that the genes may modulate immune functions, influencing antibody production and immune tolerance, potentially affected by factors like gut microbiota.

## Regulation of B-lymphocyte Activation and Proliferation¶

Key information

Key genes: ZAP70, PTPN22, FCRL3, SYK, CTLA4

Odds Ratio: 56.82

FDR: 0.000

Combined Score: 880.32

The gene set is significantly associated with the regulation of B-lymphocyte activation and proliferation, indicating its involvement in critical immune responses and lymphocyte development. These genes may play essential roles in modulating B-cell functions, potentially influencing antibody production and immune system regulation.

StringDB references

- B-lymphocyte (BTO:0000776)
- Regulation of lymphocyte proliferation (GO:0050670)
- Regulation of lymphocyte activation (GO:0051249)
- Molecular Regulation of Differentiation in Early B-Lymphocyte Development. (PMID:29966360)

Ontology genesets

- Regulation Of Lymphocyte Activation (GO:0051249)
- Regulation Of Lymphocyte Proliferation (GO:0050670)
- Regulation Of B Cell Differentiation (GO:0045577)
- Regulation Of Lymphocyte Differentiation (GO:0045619)
- Lymphocyte Differentiation (GO:0030098)

Gene overlap matrix

Gene overlap matrix

## Decreased Proliferation of Lymphocytes in Broilers¶

Key information

Key genes: ZAP70, PTPN22, SYK, LY9, SLAMF8

Odds Ratio: 52.27

FDR: 0.000

Combined Score: 791.97

The gene set is associated with decreased proliferation of lymphocytes, highlighting its involvement in critical biological processes such as lymphocyte development and activation, particularly in the context of broiler chickens. This suggests that the genes in this set may play a role in regulating immune responses, potentially influenced by factors like gut microbiota, which could affect lymphocyte function and overall immune health.

StringDB references

- Lymphocyte activation (GO:0046649)
- Regulation of lymphocyte proliferation (GO:0050670)
- Decreased lymphocyte proliferation in response to mitogen (HP:0031381)
- B Lymphocyte Development in the Bursa of Fabricius of Young Broilers is Influenced by the Gut Microbiota. (PMID:36917000)

Ontology genesets

- Regulation Of Lymphocyte Differentiation (GO:0045619)
- Lymphocyte Differentiation (GO:0030098)
- Regulation Of Lymphocyte Proliferation (GO:0050670)
- Regulation Of Lymphocyte Activation (GO:0051249)
- B Cell Activation (GO:0042113)

Gene overlap matrix

Gene overlap matrix

## Regulation of T-lymphocyte Activation and Proliferation¶

Key information

Key genes: CD4, CD8A, ZAP70, PTPN22, FCRL3

Odds Ratio: 62.22

FDR: 0.000

Combined Score: 800.67

The gene set is significantly associated with the regulation of T-lymphocyte activation and proliferation, indicating its involvement in critical immune responses. These genes may play a vital role in modulating lymphocyte activity, potentially influencing immune tolerance and response through pathways related to regulatory T-lymphocytes.

StringDB references

- Regulatory T-lymphocyte (BTO:0004520)
- Regulation of lymphocyte proliferation (GO:0050670)
- Regulation of lymphocyte activation (GO:0051249)

Ontology genesets

- Regulation Of Lymphocyte Activation (GO:0051249)
- Regulation Of Lymphocyte Differentiation (GO:0045619)
- Regulation Of T Cell Activation (GO:0050863)
- Negative Regulation Of Lymphocyte Activation (GO:0051250)
- Lymphocyte Differentiation (GO:0030098)

Gene overlap matrix

Gene overlap matrix

## Regulation of B-lymphocyte Activation and Proliferation in Bursa¶

Key information

Key genes: CD4, CD8A, FCRL3, SYK, CD5

Odds Ratio: 62.62

FDR: 0.001

Combined Score: 617.08

The gene set is significantly associated with the regulation of B-lymphocyte activation and proliferation, indicating its involvement in critical immune processes. These genes may play a vital role in modulating B cell differentiation and activation, potentially influencing immune responses and the development of B cell-related disorders.

StringDB references

- B-lymphocyte (BTO:0000776)
- Regulation of B cell differentiation (GO:0045577)
- Regulation of B cell activation (GO:0050864)
- Molecular Regulation of Differentiation in Early B-Lymphocyte Development. (PMID:29966360)

Ontology genesets

- Regulation Of B Cell Differentiation (GO:0045577)
- Regulation Of Lymphocyte Activation (GO:0051249)
- Lymphocyte Differentiation (GO:0030098)
- T Cell Receptor Complex (GO:0042101)
- Enzyme-Linked Receptor Protein Signaling Pathway (GO:0007167)

Gene overlap matrix

Gene overlap matrix


← Theme 2 - Leukocyte Signaling and Immune Regulation

Theme 4 - Autoimmune Disease Pathogenesis and Genetics  →

Page contents

- Theme 3 - Lymphocyte Activation and Proliferation Regulation
  - Regulation of B-lymphocyte Activation and Proliferation
  - Decreased Proliferation of Lymphocytes in Broilers
  - Regulation of T-lymphocyte Activation and Proliferation
  - Regulation of B-lymphocyte Activation and Proliferation in Bursa

- Contact
- Developers
