# Supplementary material for: GeneInsight: Condensing gene set knowledge via language models
Source: PLoS Comput Biol. 2026 Aug 5;22(8):e1014500. doi: 10.1371/journal.pcbi.1014500 (PMC13440848; doi:10.1371/journal.pcbi.1014500)
Supplement: Supplementary file 5 [file pcbi.1014500.s003.zip › S3 Data/sphinx_builds.zip!/sphinx_builds/html_build_geneset1/build/html/cluster_1.html]

Theme 2 - Leukocyte Signaling and Immune Regulation — GeneInsight documentation


Skip to content

GeneInsight


menu

Major themes:

- Theme 1 - Lipid Transport and Protein Interactions
- Theme 2 - Leukocyte Signaling and Immune Regulation
- Theme 3 - Lymphocyte Activation and Proliferation Regulation
- Theme 4 - Autoimmune Disease Pathogenesis and Genetics
- Theme 5 - Cancer Pathways and Immune Response
- Theme 6 - Nitrogen Metabolism Regulation in Biological Processes
- Theme 7 - Aminopterin in Canine Atopic Dermatitis Treatment
- Theme 8 - Biomarkers in Triple-Negative Breast Cancer
- Theme 9 - Immune Response Regulation in Coral Bleaching
- Theme 10 - Hepatitis Viruses in Pregnancy and Cancer

Metadata:

- Embedding map
- Summary statistics
- Data Files
- Downloads


Toggle menu contents

1. Docs
2. Theme 2 - Leukocyte Signaling and Immune Regulation

JavaScript is required to toggle light/dark mode..


 Light mode
 Dark mode


Edit on GitHub


View source

---

# Theme 2 - Leukocyte Signaling and Immune Regulation¶

The gene set is significantly associated with various pathways related to immune response, including non-receptor tyrosine kinase interactions, NK cell function regulation, T cell receptor signaling, and hemopoiesis. This suggests that the genes may play crucial roles in modulating immune cell activities and blood cell development, potentially influencing therapeutic strategies for autoimmune diseases and enhancing anti-tumor responses.

## Non-receptor tyrosine kinase interactions in leukocyte signaling¶

Key information

Key genes: CD4, CD8A, LCK, PTPN22, CD8B

Odds Ratio: 82.21

FDR: 0.000

Combined Score: 1655.44

The gene set is significantly associated with non-receptor tyrosine kinase interactions in leukocyte signaling, highlighting its involvement in critical biological processes related to immune response and autoimmunity. These genes may play a pivotal role in modulating leukocyte signaling pathways, potentially influencing therapeutic strategies for autoimmune diseases through their interactions with receptor tyrosine kinases.

StringDB references

- Transmembrane receptor protein tyrosine kinase signaling pathway (GO:0007169)
- Protein tyrosine kinase binding (GO:1990782)
- Signaling by Receptor Tyrosine Kinases (HSA-9006934)
- Non-receptor tyrosine kinase signaling in autoimmunity and therapeutic implications. (PMID:31082431)

Ontology genesets

- Non-Membrane Spanning Protein Tyrosine Kinase Activity (GO:0004715)
- Protein Tyrosine Kinase Activity (GO:0004713)
- Transmembrane Receptor Protein Tyrosine Kinase Signaling Pathway (GO:0007169)
- Protein Tyrosine Kinase Binding (GO:1990782)
- Negative Regulation Of Transmembrane Receptor Protein Serine/Threonine Kinase Signaling Pathway (GO:0090101)

Gene overlap matrix

Gene overlap matrix

## Regulation of NK Cell Function in Tumor Microenvironment¶

Key information

Key genes: LCK, CD48, CD4, CD2, CD8A

Odds Ratio: 28.56

FDR: 0.001

Combined Score: 292.53

The gene set is significantly associated with the regulation of NK cell function within the tumor microenvironment, highlighting its involvement in critical biological processes that affect immune responses to tumors. These genes may play a role in modulating NK cell activity and enhancing their ability to target and eliminate cancer cells by influencing key signaling pathways and receptor interactions.

StringDB references

- Exploiting NK Cell Surveillance Pathways for Cancer Therapy. (PMID:30626155)
- Research Progress on NK Cell Receptors and Their Signaling Pathways. (PMID:32774149)
- The Tumor Microenvironment-A Metabolic Obstacle to NK Cells Activity. (PMID:33260925)
- Charting a killer course to the solid tumor: strategies to recruit and activate NK cells in the tumor microenvironment. (PMID:38022679)

Ontology genesets

- Leukocyte Cell-Cell Adhesion (GO:0007159)
- Regulation Of Interleukin-12 Production (GO:0032655)
- Regulation Of Tumor Necrosis Factor Production (GO:0032680)
- Positive Regulation Of T Cell Proliferation (GO:0042102)
- Regulation Of Interleukin-10 Production (GO:0032653)

Gene overlap matrix

Gene overlap matrix

## Endothelial Cell Function in Blood Vessel Homeostasis¶

Key information

Key genes: TNF, RIPK1, FADD, CFLAR, TNFRSF25

Odds Ratio: 39.16

FDR: 0.000

Combined Score: 443.71

The gene set is significantly associated with pathways related to endothelial cell function and blood vessel homeostasis, highlighting its involvement in critical biological processes such as regulation of apoptosis and maintenance of endothelial integrity. These genes may play a crucial role in modulating endothelial responses to stimuli, potentially influencing blood vessel stability and immune interactions, as suggested by their connections to CDC42 signaling and antigen-presenting functions.

StringDB references

- Blood vessel (BTO:0001102)
- Regulation of endothelial cell apoptotic process (GO:2000351)
- A CDC42-centered signaling unit is a dominant positive regulator of endothelial integrity. (PMID:28860633)
- Weibel Palade Bodies: Unique Secretory Organelles of Endothelial Cells that Control Blood Vessel Homeostasis. (PMID:34977047)
- Antigen-presenting function of human endothelial cells. Direct activation of resting CD8 T cells. (PMID:7989746)

Ontology genesets

- Positive Regulation Of Tumor Necrosis Factor Production (GO:0032760)
- Positive Regulation Of Tumor Necrosis Factor Superfamily Cytokine Production (GO:1903557)
- Positive Regulation Of Myeloid Leukocyte Differentiation (GO:0002763)
- Positive Regulation Of Programmed Cell Death (GO:0043068)
- Positive Regulation Of Cytokine Production (GO:0001819)

Gene overlap matrix

Gene overlap matrix

## Positive regulation of T cell receptor signaling pathways¶

Key information

Key genes: CD8A, LCK, CD3D, CD247, CD28

Odds Ratio: 62.62

FDR: 0.001

Combined Score: 617.08

The gene set is significantly associated with the positive regulation of T cell receptor signaling pathways, indicating its involvement in enhancing T cell activation and immune responses. This suggests that the genes within this set may play crucial roles in modulating T cell functions, potentially influencing adaptive immunity and responses to infections or tumors.

StringDB references

- T cell receptor signaling pathway (GO:0050852)
- T cell receptor signaling pathway (hsa04660)

Ontology genesets

- T Cell Receptor Signaling Pathway (GO:0050852)
- Positive Regulation Of T Cell Activation (GO:0050870)
- Regulation Of T Cell Activation (GO:0050863)
- T Cell Receptor Complex (GO:0042101)
- T Cell Activation (GO:0042110)

Gene overlap matrix

Gene overlap matrix

## Multipotent Hematopoietic Stem Cells in Hemopoiesis¶

Key information

Key genes: CD4, CD5, CD8A, LCK, PTPN11

Odds Ratio: 39.16

FDR: 0.000

Combined Score: 443.71

The gene set is significantly associated with pathways involved in hemopoiesis, indicating its role in the regulation and development of blood cells. These genes may influence the differentiation and proliferation of multipotent hematopoietic stem cells, thereby impacting the overall hematopoietic cell lineage and contributing to blood cell formation.

StringDB references

- Hematopoietic cell (BTO:0000574)
- Hemopoiesis (GO:0030097)
- Regulation of hemopoiesis (GO:1903706)
- Hematopoietic cell lineage (hsa04640)

Ontology genesets

- Regulation Of Interleukin-6 Production (GO:0032675)
- T Cell Activation (GO:0042110)
- T-helper Cell Lineage Commitment (GO:0002295)
- Lymphocyte Differentiation (GO:0030098)
- Regulation Of Interleukin-8 Production (GO:0032677)

Gene overlap matrix

Gene overlap matrix


← Theme 1 - Lipid Transport and Protein Interactions

Theme 3 - Lymphocyte Activation and Proliferation Regulation  →

Page contents

- Theme 2 - Leukocyte Signaling and Immune Regulation
  - Non-receptor tyrosine kinase interactions in leukocyte signaling
  - Regulation of NK Cell Function in Tumor Microenvironment
  - Endothelial Cell Function in Blood Vessel Homeostasis
  - Positive regulation of T cell receptor signaling pathways
  - Multipotent Hematopoietic Stem Cells in Hemopoiesis

- Contact
- Developers
