# Supplementary material for: GeneInsight: Condensing gene set knowledge via language models
Source: PLoS Comput Biol. 2026 Aug 5;22(8):e1014500. doi: 10.1371/journal.pcbi.1014500 (PMC13440848; doi:10.1371/journal.pcbi.1014500)
Supplement: Supplementary file 5 [file pcbi.1014500.s003.zip › S3 Data/sphinx_builds.zip!/sphinx_builds/html_build_geneset1/build/html/cluster_5.html]

Theme 6 - Nitrogen Metabolism Regulation in Biological Processes — GeneInsight documentation


Skip to content

GeneInsight


menu

Major themes:

- Theme 1 - Lipid Transport and Protein Interactions
- Theme 2 - Leukocyte Signaling and Immune Regulation
- Theme 3 - Lymphocyte Activation and Proliferation Regulation
- Theme 4 - Autoimmune Disease Pathogenesis and Genetics
- Theme 5 - Cancer Pathways and Immune Response
- Theme 6 - Nitrogen Metabolism Regulation in Biological Processes
- Theme 7 - Aminopterin in Canine Atopic Dermatitis Treatment
- Theme 8 - Biomarkers in Triple-Negative Breast Cancer
- Theme 9 - Immune Response Regulation in Coral Bleaching
- Theme 10 - Hepatitis Viruses in Pregnancy and Cancer

Metadata:

- Embedding map
- Summary statistics
- Data Files
- Downloads


Toggle menu contents

1. Docs
2. Theme 6 - Nitrogen Metabolism Regulation in Biological Processes

JavaScript is required to toggle light/dark mode..


 Light mode
 Dark mode


Edit on GitHub


View source

---

# Theme 6 - Nitrogen Metabolism Regulation in Biological Processes¶

The gene set is significantly associated with the regulation of nitrogen-containing compound metabolic processes, indicating its involvement in critical biological pathways related to nitrogen metabolism. These genes may play essential roles in modulating the synthesis and breakdown of nitrogenous compounds, potentially influencing metabolic balance and cellular function.

## Regulation of nitrogen-containing compound metabolic processes¶

Key information

Key genes: RCOR1, CTDSP1, REST, BTRC, HDAC1

Odds Ratio: 23.72

FDR: 0.000

Combined Score: 279.32

The gene set is significantly associated with the regulation of nitrogen-containing compound metabolic processes, indicating its involvement in critical biological pathways related to nitrogen metabolism. These genes may play essential roles in modulating the synthesis and breakdown of nitrogenous compounds, potentially influencing metabolic balance and cellular function.

StringDB references

- Nitrogen compound metabolic process (GO:0006807)
- Regulation of nitrogen compound metabolic process (GO:0051171)
- Negative regulation of nitrogen compound metabolic process (GO:0051172)

Ontology genesets

- Negative Regulation Of Multicellular Organismal Process (GO:0051241)
- Regulation Of Necroptotic Process (GO:0060544)
- Regulation Of Proteolysis (GO:0030162)
- Positive Regulation Of Multicellular Organismal Process (GO:0051240)
- Protein-Lysine N-methyltransferase Activity (GO:0016279)

Gene overlap matrix

Gene overlap matrix

## Regulation of cellular nitrogen metabolic processes for macromolecule biosynthesis¶

Key information

Key genes: ATIC, PPAT, PFAS, SHMT1, SHMT2

Odds Ratio: 19.46

FDR: 0.001

Combined Score: 212.20

The gene set is significantly associated with the regulation of cellular nitrogen metabolic processes, which are crucial for macromolecule biosynthesis. This suggests that the genes may play a vital role in modulating nitrogen metabolism, potentially influencing the synthesis of essential biomolecules such as proteins and nucleic acids.

StringDB references

- Cellular nitrogen compound metabolic process (GO:0034641)
- Regulation of nitrogen compound metabolic process (GO:0051171)

Ontology genesets

- Positive Regulation Of Protein Metabolic Process (GO:0051247)
- rRNA Metabolic Process (GO:0016072)
- Regulation Of Proteolysis (GO:0030162)
- Regulation Of Transcription By RNA Polymerase II (GO:0006357)
- GMP Biosynthetic Process (GO:0006177)

Gene overlap matrix

Gene overlap matrix


← Theme 5 - Cancer Pathways and Immune Response

Theme 7 - Aminopterin in Canine Atopic Dermatitis Treatment  →

Page contents

- Theme 6 - Nitrogen Metabolism Regulation in Biological Processes
  - Regulation of nitrogen-containing compound metabolic processes
  - Regulation of cellular nitrogen metabolic processes for macromolecule biosynthesis

- Contact
- Developers
