# Supplementary material for: GeneInsight: Condensing gene set knowledge via language models
Source: PLoS Comput Biol. 2026 Aug 5;22(8):e1014500. doi: 10.1371/journal.pcbi.1014500 (PMC13440848; doi:10.1371/journal.pcbi.1014500)
Supplement: Supplementary file 5 [file pcbi.1014500.s003.zip › S3 Data/sphinx_builds.zip!/sphinx_builds/html_build_geneset1/build/html/search.html]

Search — GeneInsight documentation


Skip to content

GeneInsight


menu

Major themes:

- Theme 1 - Lipid Transport and Protein Interactions
- Theme 2 - Leukocyte Signaling and Immune Regulation
- Theme 3 - Lymphocyte Activation and Proliferation Regulation
- Theme 4 - Autoimmune Disease Pathogenesis and Genetics
- Theme 5 - Cancer Pathways and Immune Response
- Theme 6 - Nitrogen Metabolism Regulation in Biological Processes
- Theme 7 - Aminopterin in Canine Atopic Dermatitis Treatment
- Theme 8 - Biomarkers in Triple-Negative Breast Cancer
- Theme 9 - Immune Response Regulation in Coral Bleaching
- Theme 10 - Hepatitis Viruses in Pregnancy and Cancer

Metadata:

- Embedding map
- Summary statistics
- Data Files
- Downloads


Toggle menu contents

1. Docs
2. Search

JavaScript is required to toggle light/dark mode..


 Light mode
 Dark mode

---

Please activate JavaScript to enable the search
functionality.

- Contact
- Developers
