# Supplementary material for: GeneInsight: Condensing gene set knowledge via language models
Source: PLoS Comput Biol. 2026 Aug 5;22(8):e1014500. doi: 10.1371/journal.pcbi.1014500 (PMC13440848; doi:10.1371/journal.pcbi.1014500)
Supplement: Supplementary file 5 [file pcbi.1014500.s003.zip › S3 Data/sphinx_builds.zip!/sphinx_builds/html_build_geneset1/build/html/cluster_3.html]

Theme 4 - Autoimmune Disease Pathogenesis and Genetics — GeneInsight documentation


Skip to content

GeneInsight


menu

Major themes:

- Theme 1 - Lipid Transport and Protein Interactions
- Theme 2 - Leukocyte Signaling and Immune Regulation
- Theme 3 - Lymphocyte Activation and Proliferation Regulation
- Theme 4 - Autoimmune Disease Pathogenesis and Genetics
- Theme 5 - Cancer Pathways and Immune Response
- Theme 6 - Nitrogen Metabolism Regulation in Biological Processes
- Theme 7 - Aminopterin in Canine Atopic Dermatitis Treatment
- Theme 8 - Biomarkers in Triple-Negative Breast Cancer
- Theme 9 - Immune Response Regulation in Coral Bleaching
- Theme 10 - Hepatitis Viruses in Pregnancy and Cancer

Metadata:

- Embedding map
- Summary statistics
- Data Files
- Downloads


Toggle menu contents

1. Docs
2. Theme 4 - Autoimmune Disease Pathogenesis and Genetics

JavaScript is required to toggle light/dark mode..


 Light mode
 Dark mode


Edit on GitHub


View source

---

# Theme 4 - Autoimmune Disease Pathogenesis and Genetics¶

The gene set is significantly associated with pathways involved in autoimmune diseases, particularly rheumatoid arthritis and alopecia areata, emphasizing key biological processes such as inflammation and immune regulation. These genes may modulate immune responses and contribute to the pathogenesis of autoimmune conditions through mechanisms like genetic variation affecting immune signaling pathways.

## Gene interactions in rheumatoid arthritis pathogenesis and development¶

Key information

Key genes: PTPN22, CTLA4, HLA-DRB1, PADI4, FCRL3

Odds Ratio: 74.01

FDR: 0.001

Combined Score: 758.88

The gene set is significantly associated with pathways involved in the pathogenesis and development of rheumatoid arthritis, highlighting key biological processes such as inflammation and immune response. These genes may play crucial roles in modulating the inflammatory pathways and gene interactions that contribute to the onset and progression of rheumatoid arthritis.

StringDB references

- Rheumatoid arthritis-associated gene-gene interaction network for rheumatoid arthritis candidate genes. (PMID:20018070)
- A pathway analysis applied to Genetic Analysis Workshop 16 genome-wide rheumatoid arthritis data. (PMID:20018088)
- Recent Advances in Defining the Genetic Basis of Rheumatoid Arthritis. (PMID:27216775)
- Genes associated with the development of rheumatoid arthritis (WP5033)

Ontology genesets

- Antigen Receptor-Mediated Signaling Pathway (GO:0050851)
- Regulation Of Lymphocyte Activation (GO:0051249)
- Lymphocyte Differentiation (GO:0030098)
- Enzyme-Linked Receptor Protein Signaling Pathway (GO:0007167)
- T Cell Receptor Complex (GO:0042101)

Gene overlap matrix

Gene overlap matrix

## Genetic Variants Influencing Autoimmunity in Alopecia Areata¶

Key information

Key genes: CTLA4, PTPN22, HLA-DRB1, ZAP70, LCK

Odds Ratio: 74.01

FDR: 0.001

Combined Score: 758.88

The gene set is significantly associated with pathways related to autoimmunity, particularly in the context of alopecia areata, highlighting the role of genetic variants such as PTPN22, IL2RA, and CTLA4 in influencing immune responses. These genes may contribute to the pathogenesis of alopecia areata by modulating immune signaling pathways, potentially leading to dysregulated hair follicle immune interactions and hair loss.

StringDB references

- Alopecia areata (DOID:986)
- Autoimmunity (HP:0002960)
- Mutations in the cholesterol transporter gene ABCA5 are associated with excessive hair overgrowth. (PMID:24831815)
- Effect of PTPN22, FASFASL, IL2RA and CTLA4 genetic polymorphisms on the risk of developing alopecia areata: A systematic review of the literature and meta-analysis. (PMID:34735462)
- Functional Genetics to Understand the Etiology of Autoimmunity. (PMID:36980846)

Ontology genesets

- Lymphocyte Differentiation (GO:0030098)
- T Cell Receptor Complex (GO:0042101)
- Antigen Receptor-Mediated Signaling Pathway (GO:0050851)
- T Cell Activation (GO:0042110)
- T Cell Differentiation (GO:0030217)

Gene overlap matrix

Gene overlap matrix

## Genetic Polymorphisms in Autoimmune Disease Susceptibility¶

Key information

Key genes: CD8A, CD4, CD2, CTLA4, CD247

Odds Ratio: 42.30

FDR: 0.000

Combined Score: 490.32

The gene set is significantly associated with pathways related to autoimmune disease susceptibility, highlighting enriched biological processes involved in immune regulation and genetic predisposition. These genes may play critical roles in modulating immune responses and contributing to the development of autoimmune conditions, potentially through mechanisms such as genetic variation affecting immune cell function and signaling pathways.

StringDB references

- Autoimmune disease (DOID:417)
- Autoimmunity (HP:0002960)
- Genetic and epigenetic fine mapping of causal autoimmune disease variants. (PMID:25363779)
- Functional Genetics to Understand the Etiology of Autoimmunity. (PMID:36980846)

Ontology genesets

- Antigen Receptor-Mediated Signaling Pathway (GO:0050851)
- T Cell Receptor Complex (GO:0042101)
- Lymphocyte Differentiation (GO:0030098)
- Regulation Of Lymphocyte Activation (GO:0051249)
- Positive Regulation Of Interleukin-2 Production (GO:0032743)

Gene overlap matrix

Gene overlap matrix

## Genetic Polymorphisms in Autoimmune Disorders Related to PTPN22¶

Key information

Key genes: PTPN22, CSK, ZAP70, LCK, CD8A

Odds Ratio: 90.46

FDR: 0.001

Combined Score: 969.55

The gene set is significantly associated with pathways related to autoimmune disorders, particularly highlighting the role of the PTPN22 gene in various autoimmune conditions such as type 1 diabetes and Graves disease. This suggests that the genes in this set may influence immune regulation and susceptibility to autoimmunity, potentially through mechanisms involving innate and adaptive immune responses.

StringDB references

- Association of PTPN22 1858TT genotype with type 1 diabetes, Graves disease but not with rheumatoid arthritis in Russian population. (PMID:21467606)
- PTPN22: the archetypal non-HLA autoimmunity gene. (PMID:25003765)
- The role of PTPN22 risk variant in the development of autoimmunity: finding common ground between mouse and human. (PMID:25795788)
- In children with autoimmune thyroiditis CTLA4 and FCRL3 genes–but not PTPN22–are overexpressed when compared to adults. (PMID:26994388)
- Influence of PTPN22 Allotypes on Innate and Adaptive Immune Function in Health and Disease. (PMID:33717184)

Ontology genesets

- T Cell Receptor Complex (GO:0042101)
- T Cell Receptor Signaling Pathway (GO:0050852)
- Antigen Receptor-Mediated Signaling Pathway (GO:0050851)
- T Cell Activation (GO:0042110)
- Transmembrane Receptor Protein Tyrosine Kinase Signaling Pathway (GO:0007169)

Gene overlap matrix

Gene overlap matrix


← Theme 3 - Lymphocyte Activation and Proliferation Regulation

Theme 5 - Cancer Pathways and Immune Response  →

Page contents

- Theme 4 - Autoimmune Disease Pathogenesis and Genetics
  - Gene interactions in rheumatoid arthritis pathogenesis and development
  - Genetic Variants Influencing Autoimmunity in Alopecia Areata
  - Genetic Polymorphisms in Autoimmune Disease Susceptibility
  - Genetic Polymorphisms in Autoimmune Disorders Related to PTPN22

- Contact
- Developers
