# Supplementary material for: GeneInsight: Condensing gene set knowledge via language models
Source: PLoS Comput Biol. 2026 Aug 5;22(8):e1014500. doi: 10.1371/journal.pcbi.1014500 (PMC13440848; doi:10.1371/journal.pcbi.1014500)
Supplement: Supplementary file 5 [file pcbi.1014500.s003.zip › S3 Data/sphinx_builds.zip!/sphinx_builds/html_build_geneset1/build/html/cluster_6.html]

Theme 7 - Aminopterin in Canine Atopic Dermatitis Treatment — GeneInsight documentation


Skip to content

GeneInsight


menu

Major themes:

- Theme 1 - Lipid Transport and Protein Interactions
- Theme 2 - Leukocyte Signaling and Immune Regulation
- Theme 3 - Lymphocyte Activation and Proliferation Regulation
- Theme 4 - Autoimmune Disease Pathogenesis and Genetics
- Theme 5 - Cancer Pathways and Immune Response
- Theme 6 - Nitrogen Metabolism Regulation in Biological Processes
- Theme 7 - Aminopterin in Canine Atopic Dermatitis Treatment
- Theme 8 - Biomarkers in Triple-Negative Breast Cancer
- Theme 9 - Immune Response Regulation in Coral Bleaching
- Theme 10 - Hepatitis Viruses in Pregnancy and Cancer

Metadata:

- Embedding map
- Summary statistics
- Data Files
- Downloads


Toggle menu contents

1. Docs
2. Theme 7 - Aminopterin in Canine Atopic Dermatitis Treatment

JavaScript is required to toggle light/dark mode..


 Light mode
 Dark mode


Edit on GitHub


View source

---

# Theme 7 - Aminopterin in Canine Atopic Dermatitis Treatment¶

The gene set is significantly associated with pathways related to atopic dermatitis and allergy, indicating its involvement in immune response and inflammation processes. These genes may play a crucial role in modulating T cell activity and co-stimulatory pathways, potentially influencing the efficacy of treatments like aminopterin in managing atopic conditions.

## Aminopterin Efficacy in Canine Atopic Dermatitis Management¶

Key information

Key genes: PSTPIP1, HLA-DQA1, HLA-DRB1, RUNX1, IKZF3

Odds Ratio: 55.67

FDR: 0.000

Combined Score: 696.23

The gene set is significantly associated with pathways related to atopic dermatitis and allergy, indicating its involvement in immune response and inflammation processes. These genes may play a crucial role in modulating T cell activity and co-stimulatory pathways, potentially influencing the efficacy of treatments like aminopterin in managing atopic conditions.

StringDB references

- Dermatitis (DOID:2723)
- Atopic asthma (EFO:0010638)
- Allergy (HP:0012393)
- LD-aminopterin in the canine homologue of human atopic dermatitis: a randomized, controlled trial reveals dosing factors affecting optimal therapy. (PMID:25255447)
- T cell co-stimulatory and co-inhibitory pathways in atopic dermatitis. (PMID:36993982)

Ontology genesets

- Positive Regulation Of Cytokine Production (GO:0001819)
- Purine-Containing Compound Biosynthetic Process (GO:0072522)
- T Cell Activation (GO:0042110)
- T-helper 17 Cell Differentiation (GO:0072539)
- Purine Nucleotide Biosynthetic Process (GO:0006164)

Gene overlap matrix

Gene overlap matrix


← Theme 6 - Nitrogen Metabolism Regulation in Biological Processes

Theme 8 - Biomarkers in Triple-Negative Breast Cancer  →

Page contents

- Theme 7 - Aminopterin in Canine Atopic Dermatitis Treatment
  - Aminopterin Efficacy in Canine Atopic Dermatitis Management

- Contact
- Developers
