# Supplementary material for: GeneInsight: Condensing gene set knowledge via language models
Source: PLoS Comput Biol. 2026 Aug 5;22(8):e1014500. doi: 10.1371/journal.pcbi.1014500 (PMC13440848; doi:10.1371/journal.pcbi.1014500)
Supplement: Supplementary file 5 [file pcbi.1014500.s003.zip › S3 Data/sphinx_builds.zip!/sphinx_builds/html_build_geneset1/build/html/cluster_0.html]

Theme 1 - Lipid Transport and Protein Interactions — GeneInsight documentation


Skip to content

GeneInsight


menu

Major themes:

- Theme 1 - Lipid Transport and Protein Interactions
- Theme 2 - Leukocyte Signaling and Immune Regulation
- Theme 3 - Lymphocyte Activation and Proliferation Regulation
- Theme 4 - Autoimmune Disease Pathogenesis and Genetics
- Theme 5 - Cancer Pathways and Immune Response
- Theme 6 - Nitrogen Metabolism Regulation in Biological Processes
- Theme 7 - Aminopterin in Canine Atopic Dermatitis Treatment
- Theme 8 - Biomarkers in Triple-Negative Breast Cancer
- Theme 9 - Immune Response Regulation in Coral Bleaching
- Theme 10 - Hepatitis Viruses in Pregnancy and Cancer

Metadata:

- Embedding map
- Summary statistics
- Data Files
- Downloads


Toggle menu contents

1. Docs
2. Theme 1 - Lipid Transport and Protein Interactions

JavaScript is required to toggle light/dark mode..


 Light mode
 Dark mode


Edit on GitHub


View source

---

# Theme 1 - Lipid Transport and Protein Interactions¶

The gene set is significantly associated with pathways involved in intracellular lipid transport and protein transport, indicating its role in the regulation of lipid metabolism and cellular trafficking processes. These genes may facilitate the movement of lipids across cellular membranes, potentially influencing lipid homeostasis and signaling pathways related to energy metabolism.

## Intracellular Lipid Transport via Transmembrane Proteins¶

Key information

Key genes: RAB3A, ARF3, RAB27B, MLPH, MYRIP

Odds Ratio: 28.56

FDR: 0.001

Combined Score: 292.53

The gene set is significantly associated with pathways involved in intracellular lipid transport and protein transport, indicating its role in the regulation of lipid metabolism and cellular trafficking processes. These genes may facilitate the movement of lipids across cellular membranes, potentially influencing lipid homeostasis and signaling pathways related to energy metabolism.

StringDB references

- Lipid transporter activity (GO:0005319)
- Lipid transport (GO:0006869)
- Intracellular protein transport (GO:0006886)
- Intracellular transport (GO:0046907)

Ontology genesets

- Intracellular Protein Transport (GO:0006886)
- Vesicle-Mediated Transport (GO:0016192)
- RNA Transport (GO:0050658)
- mRNA Transport (GO:0051028)
- Establishment Of Protein Localization To Organelle (GO:0072594)

Gene overlap matrix

Gene overlap matrix

## Pinin-containing PSAP complex in protein-binding interactions¶

Key information

Key genes: CD8A, LCK, CD4, HLA-E, HLA-A

Odds Ratio: 39.16

FDR: 0.000

Combined Score: 443.71

The gene set is associated with the Pinin-containing PSAP complex, which plays a crucial role in protein-binding interactions, highlighting its involvement in essential biological processes related to protein complexes. This suggests that the genes in this set may be involved in mediating protein interactions and stability, potentially influencing cellular functions and signaling pathways that rely on these protein complexes.

StringDB references

- Protein binding (GO:0005515)
- Protein-containing complex binding (GO:0044877)
- The structure of the ASAP core complex reveals the existence of a Pinin-containing PSAP complex. (PMID:22388736)

Ontology genesets

- Enzyme-Linked Receptor Protein Signaling Pathway (GO:0007167)
- T Cell Receptor Complex (GO:0042101)
- MHC Protein Binding (GO:0042287)
- Antigen Receptor-Mediated Signaling Pathway (GO:0050851)
- Negative Regulation Of mRNA Processing (GO:0050686)

Gene overlap matrix

Gene overlap matrix


← GeneInsight Topic Descriptions

Theme 2 - Leukocyte Signaling and Immune Regulation  →

Page contents

- Theme 1 - Lipid Transport and Protein Interactions
  - Intracellular Lipid Transport via Transmembrane Proteins
  - Pinin-containing PSAP complex in protein-binding interactions

- Contact
- Developers
