# Supplementary material for: GeneInsight: Condensing gene set knowledge via language models
Source: PLoS Comput Biol. 2026 Aug 5;22(8):e1014500. doi: 10.1371/journal.pcbi.1014500 (PMC13440848; doi:10.1371/journal.pcbi.1014500)
Supplement: Supplementary file 5 [file pcbi.1014500.s003.zip › S3 Data/sphinx_builds.zip!/sphinx_builds/html_build_geneset1/build/html/cluster_8.html]

Theme 9 - Immune Response Regulation in Coral Bleaching — GeneInsight documentation


Skip to content

GeneInsight


menu

Major themes:

- Theme 1 - Lipid Transport and Protein Interactions
- Theme 2 - Leukocyte Signaling and Immune Regulation
- Theme 3 - Lymphocyte Activation and Proliferation Regulation
- Theme 4 - Autoimmune Disease Pathogenesis and Genetics
- Theme 5 - Cancer Pathways and Immune Response
- Theme 6 - Nitrogen Metabolism Regulation in Biological Processes
- Theme 7 - Aminopterin in Canine Atopic Dermatitis Treatment
- Theme 8 - Biomarkers in Triple-Negative Breast Cancer
- Theme 9 - Immune Response Regulation in Coral Bleaching
- Theme 10 - Hepatitis Viruses in Pregnancy and Cancer

Metadata:

- Embedding map
- Summary statistics
- Data Files
- Downloads


Toggle menu contents

1. Docs
2. Theme 9 - Immune Response Regulation in Coral Bleaching

JavaScript is required to toggle light/dark mode..


 Light mode
 Dark mode


Edit on GitHub


View source

---

# Theme 9 - Immune Response Regulation in Coral Bleaching¶

The gene set is significantly associated with the regulation of immune responses, particularly in the context of coral bleaching, as highlighted by studies on the Caribbean reef-building coral Orbicella faveolata. These genes may play crucial roles in enhancing coral innate immunity and adaptive humoral immunity, potentially influencing the resilience of corals to environmental stressors such as bleaching.

## Regulation of immune response in coral bleaching¶

Key information

Key genes: LCK, CD48, PTPN11, CD4, CD28

Odds Ratio: 27.10

FDR: 0.001

Combined Score: 272.50

The gene set is significantly associated with the regulation of immune responses, particularly in the context of coral bleaching, as highlighted by studies on the Caribbean reef-building coral Orbicella faveolata. These genes may play crucial roles in enhancing coral innate immunity and adaptive humoral immunity, potentially influencing the resilience of corals to environmental stressors such as bleaching.

StringDB references

- Regulation of immune response (GO:0050776)
- RNA-Seq of the Caribbean reef-building coral Orbicella faveolata (Scleractinia-Merulinidae) under bleaching and disease stress expands models of coral innate immunity. (PMID:26925311)
- Costimulatory Function of Cd58Cd2 Interaction in Adaptive Humoral Immunity in a Zebrafish Model. (PMID:29904386)

Ontology genesets

- Regulation Of Lymphocyte Activation (GO:0051249)
- Negative Regulation Of Antigen Receptor-Mediated Signaling Pathway (GO:0050858)
- Positive Regulation Of MAPK Cascade (GO:0043410)
- Positive Regulation Of Cell Killing (GO:0031343)
- Regulation Of MAPK Cascade (GO:0043408)

Gene overlap matrix

Gene overlap matrix


← Theme 8 - Biomarkers in Triple-Negative Breast Cancer

Theme 10 - Hepatitis Viruses in Pregnancy and Cancer  →

Page contents

- Theme 9 - Immune Response Regulation in Coral Bleaching
  - Regulation of immune response in coral bleaching

- Contact
- Developers
