# Supplementary material for: GeneInsight: Condensing gene set knowledge via language models
Source: PLoS Comput Biol. 2026 Aug 5;22(8):e1014500. doi: 10.1371/journal.pcbi.1014500 (PMC13440848; doi:10.1371/journal.pcbi.1014500)
Supplement: Supplementary file 5 [file pcbi.1014500.s003.zip › S3 Data/sphinx_builds.zip!/sphinx_builds/html_build_geneset1/build/html/cluster_4.html]

Theme 5 - Cancer Pathways and Immune Response — GeneInsight documentation


Skip to content

GeneInsight


menu

Major themes:

- Theme 1 - Lipid Transport and Protein Interactions
- Theme 2 - Leukocyte Signaling and Immune Regulation
- Theme 3 - Lymphocyte Activation and Proliferation Regulation
- Theme 4 - Autoimmune Disease Pathogenesis and Genetics
- Theme 5 - Cancer Pathways and Immune Response
- Theme 6 - Nitrogen Metabolism Regulation in Biological Processes
- Theme 7 - Aminopterin in Canine Atopic Dermatitis Treatment
- Theme 8 - Biomarkers in Triple-Negative Breast Cancer
- Theme 9 - Immune Response Regulation in Coral Bleaching
- Theme 10 - Hepatitis Viruses in Pregnancy and Cancer

Metadata:

- Embedding map
- Summary statistics
- Data Files
- Downloads


Toggle menu contents

1. Docs
2. Theme 5 - Cancer Pathways and Immune Response

JavaScript is required to toggle light/dark mode..


 Light mode
 Dark mode


Edit on GitHub


View source

---

# Theme 5 - Cancer Pathways and Immune Response¶

The gene set is significantly associated with pathways related to glioma, demyelinating lesions, and immune modulation in multiple myeloma, indicating its involvement in tumor progression and immune response mechanisms. These genes may regulate DNA methylation and immune checkpoint molecules, influencing treatment efficacy and patient prognosis in cancer.

## Transcriptomic Variations in Glioma and Demyelinating Lesions¶

Key information

Key genes: MBD4, UHRF2, ZBTB4, ZBTB38, UHRF1

Odds Ratio: 50.36

FDR: 0.000

Combined Score: 613.25

The gene set is significantly associated with pathways related to glioma and demyelinating lesions, indicating a strong involvement in tumor progression and immune response mechanisms. These genes may play critical roles in regulating DNA methylation patterns and cellular interactions within the tumor microenvironment, potentially influencing clinical outcomes in glioma patients.

StringDB references

- Modification Patterns of DNA Methylation-Related lncRNAs Regulating Genomic Instability for Improving the Clinical Outcomes and Tumour Microenvironment Characterisation of Lower-Grade Gliomas. (PMID:35359593)
- TRP Family Genes Are Differently Expressed and Correlated with Immune Response in Glioma. (PMID:35625048)
- Glioma-neuronal interactions in tumor progression: Mechanism, therapeutic strategies and perspectives (Review). (PMID:35856439)
- Integrative and comparative single-cell analysis reveals transcriptomic difference between human tumefactive demyelinating lesion and glioma. (PMID:36085357)
- DNA methylation regulator-mediated modification patterns and tumor microenvironment characterization in glioma. (PMID:36152044)

Ontology genesets

- methyl-CpG Binding (GO:0008327)
- Cytolytic Granule (GO:0044194)
- Calcium Ion Transmembrane Transporter Activity (GO:0015085)
- Calcium Ion Transmembrane Transport (GO:0070588)
- Calcium Ion Transport (GO:0006816)

Gene overlap matrix

Gene overlap matrix

## Targeting CRL4(CRBN) in Relapsed Multiple Myeloma Therapy¶

Key information

Key genes: RBX1, CRBN, DDB1, CUL4A, CUL4B

Odds Ratio: 30.20

FDR: 0.001

Combined Score: 315.13

The gene set is significantly associated with pathways involved in the therapeutic targeting of the CRL4(CRBN) E3 ligase complex in relapsed multiple myeloma, highlighting its role in critical biological processes such as immune modulation and drug resistance. These genes may contribute to the mechanisms of action of immunomodulatory drugs and CAR-T therapies, suggesting that they play a vital role in overcoming therapeutic resistance and enhancing treatment efficacy in multiple myeloma.

StringDB references

- Immunomodulatory Drugs in Multiple Myeloma: Mechanisms of Action and Clinical Experience. (PMID:28205024)
- Elotuzumab for the Treatment of Relapsed or Refractory Multiple Myeloma, with Special Reference to its Modes of Action and SLAMF7 Signaling. (PMID:29531651)
- [Molecular Mechanism of CRBN in the Activity of Lenalidomid eagainst Myeloma–Review]. (PMID:30111438)
- CRL4`(CRBN) <Unknown identifier format: CRBN>`\_ E3 Ligase Complex as a Therapeutic Target in Multiple Myeloma. (PMID:36139651)
- Identification of potential resistance mechanisms and therapeutic targets for the relapse of BCMA CAR-T therapy in relapsedrefractory multiple myeloma through single-cell sequencing. (PMID:37158921)

Ontology genesets

- Cul4A-RING E3 Ubiquitin Ligase Complex (GO:0031464)
- Cul4-RING E3 Ubiquitin Ligase Complex (GO:0080008)
- cullin-RING Ubiquitin Ligase Complex (GO:0031461)
- Natural Killer Cell Mediated Immunity (GO:0002228)
- Proteasomal Protein Catabolic Process (GO:0010498)

Gene overlap matrix

Gene overlap matrix

## HLA Class Typing and Immune Cell Infiltration in Cancer¶

Key information

Key genes: CD4, HLA-E, HLA-A, CD8A, B2M

Odds Ratio: 90.46

FDR: 0.001

Combined Score: 969.55

The gene set is significantly associated with pathways related to HLA class typing and immune cell infiltration in cancer, suggesting a crucial role in modulating the immune response within the tumor microenvironment. These genes may influence tumor progression and patient prognosis by regulating immune checkpoint molecules, such as HLA-G, which are implicated in immune evasion mechanisms in various cancers.

StringDB references

- (….) Non-classical HLA-class I expression in serous ovarian carcinoma: Correlation with the HLA-genotype, tumor infiltrating immune cells and prognosis. (PMID:26942060)
- Comprehensive Transcriptomic Analysis Reveals the Role of the Immune Checkpoint HLA-G Molecule in Cancers. (PMID:34276642)
- A Critical Assessment of the Association between HLA-G Expression by Carcinomas and Clinical Outcome. (PMID:34361031)
- HLA Expression in Relation to HLA Type in Classic Hodgkin Lymphoma Patients. (PMID:34830986)
- Integrative HLA typing of tumor and adjacent normal tissue can reveal insights into the tumor immune response. (PMID:38281021)

Ontology genesets

- MHC Class I Protein Binding (GO:0042288)
- Regulation Of Lymphocyte Activation (GO:0051249)
- MHC Class II Protein Complex Binding (GO:0023026)
- T Cell Activation (GO:0042110)
- Antigen Receptor-Mediated Signaling Pathway (GO:0050851)

Gene overlap matrix

Gene overlap matrix


← Theme 4 - Autoimmune Disease Pathogenesis and Genetics

Theme 6 - Nitrogen Metabolism Regulation in Biological Processes  →

Page contents

- Theme 5 - Cancer Pathways and Immune Response
  - Transcriptomic Variations in Glioma and Demyelinating Lesions
  - Targeting CRL4(CRBN) in Relapsed Multiple Myeloma Therapy
  - HLA Class Typing and Immune Cell Infiltration in Cancer

- Contact
- Developers
