# Supplementary material for: GeneInsight: Condensing gene set knowledge via language models
Source: PLoS Comput Biol. 2026 Aug 5;22(8):e1014500. doi: 10.1371/journal.pcbi.1014500 (PMC13440848; doi:10.1371/journal.pcbi.1014500)
Supplement: Supplementary file 6 [file pcbi.1014500.s004.zip › S4 Data/sphinx_builds.zip!/sphinx_builds/html_build_geneset3/build/html/cluster_7.html]

Theme 8 - CD4+ T Cell Activation Mechanisms — GeneInsight documentation


Skip to content

GeneInsight


menu

Major themes:

- Theme 1 - Regulation of Biosynthetic Processes in Cancer
- Theme 2 - ER Stress and Apoptosis Mechanisms
- Theme 3 - Ethanol Effects on Ceramide Metabolism
- Theme 4 - Membrane-Bounded Organelle Functions and Assembly
- Theme 5 - Unfolded Protein Response in Disease
- Theme 6 - Transcription Factor Activity Regulation in Elongation
- Theme 7 - FGFR2 Mutation and Osteocyte Function in Osteoporosis
- Theme 8 - CD4+ T Cell Activation Mechanisms
- Theme 9 - Regulation of Transcription Factor Activity
- Theme 10 - Epithelial Cell Differentiation and Proliferation

Metadata:

- Embedding map
- Summary statistics
- Data Files
- Downloads


Toggle menu contents

1. Docs
2. Theme 8 - CD4+ T Cell Activation Mechanisms

JavaScript is required to toggle light/dark mode..


 Light mode
 Dark mode


Edit on GitHub


View source

---

# Theme 8 - CD4+ T Cell Activation Mechanisms¶

The gene set is significantly associated with pathways involved in the co-stimulation and activation of CD4+ T cells, highlighting its role in immune response regulation. These genes may contribute to the modulation of T cell activation mechanisms, potentially influencing co-stimulatory pathways and enhancing immune responses against pathogens.

## Mechanisms of CD4+ T Cell Co-Stimulation and Activation¶

Key information

Key genes: TRAF5, TNFRSF8, CD27, TNFSF4, CD40

Odds Ratio: 23.24

FDR: 0.001

Combined Score: 221.65

The gene set is significantly associated with pathways involved in the co-stimulation and activation of CD4+ T cells, highlighting its role in immune response regulation. These genes may contribute to the modulation of T cell activation mechanisms, potentially influencing co-stimulatory pathways and enhancing immune responses against pathogens.

StringDB references

- Regulation of CD4-positive, alpha-beta T cell activation (GO:2000514)
- Molecular mechanisms of T cell co-stimulation and co-inhibition. (PMID:23470321)
- Engagement of distinct epitopes on CD43 induces different co-stimulatory pathways in human T cells. (PMID:27392084)
- Autologous blood extracellular vesicles and specific CD4`(+) <Unknown identifier format: +>`\_ T-cell co-activation. (PMID:36172364)

Ontology genesets

- T Cell Activation (GO:0042110)
- CD40 Receptor Complex (GO:0035631)
- Regulation Of T-helper 1 Cell Differentiation (GO:0045625)
- Regulation Of Alpha-Beta T Cell Proliferation (GO:0046640)
- Positive Regulation Of Lymphocyte Proliferation (GO:0050671)

Gene overlap matrix

Gene overlap matrix


← Theme 7 - FGFR2 Mutation and Osteocyte Function in Osteoporosis

Theme 9 - Regulation of Transcription Factor Activity  →

Page contents

- Theme 8 - CD4+ T Cell Activation Mechanisms
  - Mechanisms of CD4+ T Cell Co-Stimulation and Activation

- Contact
- Developers
