# Supplementary material for: GeneInsight: Condensing gene set knowledge via language models
Source: PLoS Comput Biol. 2026 Aug 5;22(8):e1014500. doi: 10.1371/journal.pcbi.1014500 (PMC13440848; doi:10.1371/journal.pcbi.1014500)
Supplement: Supplementary file 6 [file pcbi.1014500.s004.zip › S4 Data/sphinx_builds.zip!/sphinx_builds/html_build_geneset3/build/html/summary.html]

Embedding map — GeneInsight documentation


Skip to content

GeneInsight


menu

Major themes:

- Theme 1 - Regulation of Biosynthetic Processes in Cancer
- Theme 2 - ER Stress and Apoptosis Mechanisms
- Theme 3 - Ethanol Effects on Ceramide Metabolism
- Theme 4 - Membrane-Bounded Organelle Functions and Assembly
- Theme 5 - Unfolded Protein Response in Disease
- Theme 6 - Transcription Factor Activity Regulation in Elongation
- Theme 7 - FGFR2 Mutation and Osteocyte Function in Osteoporosis
- Theme 8 - CD4+ T Cell Activation Mechanisms
- Theme 9 - Regulation of Transcription Factor Activity
- Theme 10 - Epithelial Cell Differentiation and Proliferation

Metadata:

- Embedding map
- Summary statistics
- Data Files
- Downloads


Toggle menu contents

1. Docs
2. Embedding map

JavaScript is required to toggle light/dark mode..


 Light mode
 Dark mode


Edit on GitHub


View source

---

# Embedding map¶

This figure is a two-dimensional map where each theme heading is positioned based on the combined semantic information from the main topics and all enriched gene set themes.

# Summary statistics¶

The following table provides a summary of the key statistics derived from the analysis:

| Statistic | Value |
| --- | --- |
| Number of genes considered | 100 |
| Documents considered | 33820 |
| Average topics | 4 |
| Range of max topics | 1 to 6 |
| Api calls made | 6786 |
| Number of themes after filtering | 25 |
| Number of clusters | 10 |
| Compression ratio | 1352.8 |
| Time of analysis | 2025-04-21T20:15:52.765704 |

# Data Files¶

The enriched gene sets represent all AI-generated themes, without condensing and filtering. The subheading data represents a detailed breakdown and information of all API calls for subheading generation.

- `enriched_genesets.csv`
- `subheading_data.csv`


← Theme 10 - Epithelial Cell Differentiation and Proliferation

Downloads  →

Page contents

- Embedding map
- Summary statistics
- Data Files

- Contact
- Developers
