# Supplementary material for: GeneInsight: Condensing gene set knowledge via language models
Source: PLoS Comput Biol. 2026 Aug 5;22(8):e1014500. doi: 10.1371/journal.pcbi.1014500 (PMC13440848; doi:10.1371/journal.pcbi.1014500)
Supplement: Supplementary file 6 [file pcbi.1014500.s004.zip › S4 Data/sphinx_builds.zip!/sphinx_builds/html_build_geneset3/build/html/cluster_9.html]

Theme 10 - Epithelial Cell Differentiation and Proliferation — GeneInsight documentation


Skip to content

GeneInsight


menu

Major themes:

- Theme 1 - Regulation of Biosynthetic Processes in Cancer
- Theme 2 - ER Stress and Apoptosis Mechanisms
- Theme 3 - Ethanol Effects on Ceramide Metabolism
- Theme 4 - Membrane-Bounded Organelle Functions and Assembly
- Theme 5 - Unfolded Protein Response in Disease
- Theme 6 - Transcription Factor Activity Regulation in Elongation
- Theme 7 - FGFR2 Mutation and Osteocyte Function in Osteoporosis
- Theme 8 - CD4+ T Cell Activation Mechanisms
- Theme 9 - Regulation of Transcription Factor Activity
- Theme 10 - Epithelial Cell Differentiation and Proliferation

Metadata:

- Embedding map
- Summary statistics
- Data Files
- Downloads


Toggle menu contents

1. Docs
2. Theme 10 - Epithelial Cell Differentiation and Proliferation

JavaScript is required to toggle light/dark mode..


 Light mode
 Dark mode


Edit on GitHub


View source

---

# Theme 10 - Epithelial Cell Differentiation and Proliferation¶

The gene set is significantly associated with pathways involved in the positive regulation of epithelial cell differentiation and proliferation, indicating its role in enhancing these critical biological processes. This suggests that the genes within this set may contribute to mechanisms that promote epithelial tissue development and maintenance, potentially influencing processes such as wound healing and tissue regeneration.

## Positive regulation of epithelial cell differentiation and proliferation¶

Key information

Key genes: MAFG, NFE2L1, MAFF, BMP4, BMP6

Odds Ratio: 58.81

FDR: 0.000

Combined Score: 746.17

The gene set is significantly associated with pathways involved in the positive regulation of epithelial cell differentiation and proliferation, indicating its role in enhancing these critical biological processes. This suggests that the genes within this set may contribute to mechanisms that promote epithelial tissue development and maintenance, potentially influencing processes such as wound healing and tissue regeneration.

StringDB references

- Regulation of epithelial cell differentiation (GO:0030856)
- Positive regulation of epithelial cell differentiation (GO:0030858)
- Positive regulation of epithelial cell proliferation (GO:0050679)

Ontology genesets

- Positive Regulation Of Epithelial Cell Proliferation (GO:0050679)
- Regulation Of Epithelial Cell Differentiation (GO:0030856)
- Positive Regulation Of Cell Differentiation (GO:0045597)
- Positive Regulation Of Glial Cell Differentiation (GO:0045687)
- Negative Regulation Of Cell Differentiation (GO:0045596)

Gene overlap matrix

Gene overlap matrix

## Regulation of Epithelial Cell Fate Commitment and Differentiation¶

Key information

Key genes: BMP4, MAFG, NFE2L1, MAFF, BMPR1A

Odds Ratio: 76.92

FDR: 0.000

Combined Score: 1040.71

The gene set is significantly associated with pathways involved in the regulation of epithelial cell fate commitment and differentiation, indicating its role in influencing how epithelial cells develop and specialize. This suggests that the genes in this set may be crucial for modulating epithelial cell behavior, potentially impacting processes such as tissue repair and regeneration through their involvement in the positive regulation of epithelial cell differentiation.

StringDB references

- Regulation of cell fate commitment (GO:0010453)
- Regulation of epithelial cell differentiation (GO:0030856)
- Positive regulation of epithelial cell differentiation (GO:0030858)
- Epithelial cell fate commitment (GO:0072148)

Ontology genesets

- Regulation Of Epithelial Cell Differentiation (GO:0030856)
- Positive Regulation Of Epithelial Cell Proliferation (GO:0050679)
- Regulation Of Epidermal Cell Differentiation (GO:0045604)
- Positive Regulation Of Epithelial To Mesenchymal Transition (GO:0010718)
- Positive Regulation Of Cell Differentiation (GO:0045597)

Gene overlap matrix

Gene overlap matrix


← Theme 9 - Regulation of Transcription Factor Activity

Embedding map  →

Page contents

- Theme 10 - Epithelial Cell Differentiation and Proliferation
  - Positive regulation of epithelial cell differentiation and proliferation
  - Regulation of Epithelial Cell Fate Commitment and Differentiation

- Contact
- Developers
