# Supplementary material for: GeneInsight: Condensing gene set knowledge via language models
Source: PLoS Comput Biol. 2026 Aug 5;22(8):e1014500. doi: 10.1371/journal.pcbi.1014500 (PMC13440848; doi:10.1371/journal.pcbi.1014500)
Supplement: Supplementary file 6 [file pcbi.1014500.s004.zip › S4 Data/sphinx_builds.zip!/sphinx_builds/html_build_geneset3/build/html/download.html]

Downloads — GeneInsight documentation


Skip to content

GeneInsight


menu

Major themes:

- Theme 1 - Regulation of Biosynthetic Processes in Cancer
- Theme 2 - ER Stress and Apoptosis Mechanisms
- Theme 3 - Ethanol Effects on Ceramide Metabolism
- Theme 4 - Membrane-Bounded Organelle Functions and Assembly
- Theme 5 - Unfolded Protein Response in Disease
- Theme 6 - Transcription Factor Activity Regulation in Elongation
- Theme 7 - FGFR2 Mutation and Osteocyte Function in Osteoporosis
- Theme 8 - CD4+ T Cell Activation Mechanisms
- Theme 9 - Regulation of Transcription Factor Activity
- Theme 10 - Epithelial Cell Differentiation and Proliferation

Metadata:

- Embedding map
- Summary statistics
- Data Files
- Downloads


Toggle menu contents

1. Docs
2. Downloads

JavaScript is required to toggle light/dark mode..


 Light mode
 Dark mode


Edit on GitHub


View source

---

# Downloads¶

Select the themes you want to download by checking the boxes. Click “Download Selected” to get a ZIP file of the selected themes.

0

Themes selected

Theme 1 - Regulation of Biosynthetic Processes in Cancer

Theme 2 - ER Stress and Apoptosis Mechanisms

Theme 3 - Ethanol Effects on Ceramide Metabolism

Theme 4 - Membrane-Bounded Organelle Functions and Assembly

Theme 5 - Unfolded Protein Response in Disease

Theme 6 - Transcription Factor Activity Regulation in Elongation

Theme 7 - FGFR2 Mutation and Osteocyte Function in Osteoporosis

Theme 8 - CD4+ T Cell Activation Mechanisms

Theme 9 - Regulation of Transcription Factor Activity

Theme 10 - Epithelial Cell Differentiation and Proliferation

Select All Themes
Download Selected


← Embedding map

- Contact
- Developers
