# Supplementary material for: GeneInsight: Condensing gene set knowledge via language models
Source: PLoS Comput Biol. 2026 Aug 5;22(8):e1014500. doi: 10.1371/journal.pcbi.1014500 (PMC13440848; doi:10.1371/journal.pcbi.1014500)
Supplement: Supplementary file 6 [file pcbi.1014500.s004.zip › S4 Data/sphinx_builds.zip!/sphinx_builds/html_build_geneset3/build/html/cluster_2.html]

Theme 3 - Ethanol Effects on Ceramide Metabolism — GeneInsight documentation


Skip to content

GeneInsight


menu

Major themes:

- Theme 1 - Regulation of Biosynthetic Processes in Cancer
- Theme 2 - ER Stress and Apoptosis Mechanisms
- Theme 3 - Ethanol Effects on Ceramide Metabolism
- Theme 4 - Membrane-Bounded Organelle Functions and Assembly
- Theme 5 - Unfolded Protein Response in Disease
- Theme 6 - Transcription Factor Activity Regulation in Elongation
- Theme 7 - FGFR2 Mutation and Osteocyte Function in Osteoporosis
- Theme 8 - CD4+ T Cell Activation Mechanisms
- Theme 9 - Regulation of Transcription Factor Activity
- Theme 10 - Epithelial Cell Differentiation and Proliferation

Metadata:

- Embedding map
- Summary statistics
- Data Files
- Downloads


Toggle menu contents

1. Docs
2. Theme 3 - Ethanol Effects on Ceramide Metabolism

JavaScript is required to toggle light/dark mode..


 Light mode
 Dark mode


Edit on GitHub


View source

---

# Theme 3 - Ethanol Effects on Ceramide Metabolism¶

The gene set is significantly associated with pathways involved in ceramide metabolism and cellular responses to ethanol, indicating its role in energy balance, neuroprotection, and alcohol metabolism. These genes may regulate the balance between neuroprotective and toxic effects of ethanol, influencing metabolic processes in the brain and liver, and mediating acute responses to ethanol exposure.

## Impact of Ethanol on Ceramide Metabolism in Alcohol-Preferring Rats¶

Key information

Key genes: ASAH1, CERS6, SPHK1, SPHK2, SGPL1

Odds Ratio: 24.37

FDR: 0.001

Combined Score: 236.55

The gene set is significantly associated with pathways involved in ceramide metabolism and its regulation by ethanol, suggesting a role in energy balance and neuroprotection. These genes may influence the balance between neuroprotective and toxic effects of ethanol through their involvement in ceramide pathways, potentially impacting metabolic processes in the brain and liver.

StringDB references

- Acute effects of oral and intravenous ethanol on rat hepatic enzyme activities. (PMID:179581)
- Effects of ethanol metabolites on exocytosis of pancreatic acinar cells in rats. (PMID:22710192)
- Ceramide metabolism analysis in a model of binge drinking reveals both neuroprotective and toxic effects of ethanol. (PMID:25060779)
- Chronic Voluntary Ethanol Consumption Induces Favorable Ceramide Profiles in Selectively Bred Alcohol-Preferring (P) Rats. (PMID:26405804)
- The Role of Fatty Acids in Ceramide Pathways and Their Influence on Hypothalamic Regulation of Energy Balance: A Systematic Review. (PMID:34069652)

Ontology genesets

- Ceramide Metabolic Process (GO:0006672)
- Regulation Of Ceramide Biosynthetic Process (GO:2000303)
- Ceramide Catabolic Process (GO:0046514)
- Ceramide Biosynthetic Process (GO:0046513)
- Hexose Metabolic Process (GO:0019318)

Gene overlap matrix

Gene overlap matrix

## Cellular Mechanisms of Ethanol-Induced Responses in Rats¶

Key information

Key genes: LEP, PPARA, ADIPOQ, CAT, SOD1

Odds Ratio: 35.09

FDR: 0.000

Combined Score: 473.91

The gene set is significantly associated with pathways related to cellular responses to ethanol, indicating its involvement in biological processes such as alcohol metabolism and the regulation of hepatic enzyme activities. These genes may play a crucial role in mediating the acute effects of ethanol exposure, potentially influencing mechanisms like exocytosis in pancreatic cells and overall metabolic responses in the liver.

StringDB references

- Response to ethanol (GO:0045471)
- Cellular response to alcohol (GO:0097306)
- Acute effects of oral and intravenous ethanol on rat hepatic enzyme activities. (PMID:179581)
- Effects of ethanol metabolites on exocytosis of pancreatic acinar cells in rats. (PMID:22710192)

Ontology genesets

- Cellular Response To Alcohol (GO:0097306)
- Cellular Response To Monoamine Stimulus (GO:0071868)
- Cellular Response To Ketone (GO:1901655)
- Cellular Response To Superoxide (GO:0071451)
- Negative Regulation Of Carbohydrate Metabolic Process (GO:0045912)

Gene overlap matrix

Gene overlap matrix

## Impact of Ethanol on Ceramide Profiles in Rats¶

Key information

Key genes: ASAH1, CERS6, CERS4, SPHK1, SPHK2

Odds Ratio: 24.37

FDR: 0.001

Combined Score: 236.55

The gene set is significantly associated with pathways related to ceramide metabolism and ethanol’s impact on metabolic processes, suggesting a role in both neuroprotection and toxicity. These genes may be involved in regulating ceramide profiles that influence metabolic diseases and the physiological response to ethanol consumption.

StringDB references

- Acute effects of oral and intravenous ethanol on rat hepatic enzyme activities. (PMID:179581)
- Effects of ethanol metabolites on exocytosis of pancreatic acinar cells in rats. (PMID:22710192)
- Ceramide metabolism analysis in a model of binge drinking reveals both neuroprotective and toxic effects of ethanol. (PMID:25060779)
- Chronic Voluntary Ethanol Consumption Induces Favorable Ceramide Profiles in Selectively Bred Alcohol-Preferring (P) Rats. (PMID:26405804)
- Contribution of specific ceramides to obesity-associated metabolic diseases. (PMID:35789435)

Ontology genesets

- Ceramide Metabolic Process (GO:0006672)
- Ceramide Catabolic Process (GO:0046514)
- Regulation Of Ceramide Biosynthetic Process (GO:2000303)
- Ceramide Biosynthetic Process (GO:0046513)
- Bioactive Lipid Receptor Activity (GO:0045125)

Gene overlap matrix

Gene overlap matrix


← Theme 2 - ER Stress and Apoptosis Mechanisms

Theme 4 - Membrane-Bounded Organelle Functions and Assembly  →

Page contents

- Theme 3 - Ethanol Effects on Ceramide Metabolism
  - Impact of Ethanol on Ceramide Metabolism in Alcohol-Preferring Rats
  - Cellular Mechanisms of Ethanol-Induced Responses in Rats
  - Impact of Ethanol on Ceramide Profiles in Rats

- Contact
- Developers
