# Supplementary material for: GeneInsight: Condensing gene set knowledge via language models
Source: PLoS Comput Biol. 2026 Aug 5;22(8):e1014500. doi: 10.1371/journal.pcbi.1014500 (PMC13440848; doi:10.1371/journal.pcbi.1014500)
Supplement: Supplementary file 6 [file pcbi.1014500.s004.zip › S4 Data/sphinx_builds.zip!/sphinx_builds/html_build_geneset3/build/html/cluster_1.html]

Theme 2 - ER Stress and Apoptosis Mechanisms — GeneInsight documentation


Skip to content

GeneInsight


menu

Major themes:

- Theme 1 - Regulation of Biosynthetic Processes in Cancer
- Theme 2 - ER Stress and Apoptosis Mechanisms
- Theme 3 - Ethanol Effects on Ceramide Metabolism
- Theme 4 - Membrane-Bounded Organelle Functions and Assembly
- Theme 5 - Unfolded Protein Response in Disease
- Theme 6 - Transcription Factor Activity Regulation in Elongation
- Theme 7 - FGFR2 Mutation and Osteocyte Function in Osteoporosis
- Theme 8 - CD4+ T Cell Activation Mechanisms
- Theme 9 - Regulation of Transcription Factor Activity
- Theme 10 - Epithelial Cell Differentiation and Proliferation

Metadata:

- Embedding map
- Summary statistics
- Data Files
- Downloads


Toggle menu contents

1. Docs
2. Theme 2 - ER Stress and Apoptosis Mechanisms

JavaScript is required to toggle light/dark mode..


 Light mode
 Dark mode


Edit on GitHub


View source

---

# Theme 2 - ER Stress and Apoptosis Mechanisms¶

The gene set is significantly associated with pathways involved in the endoplasmic reticulum (ER) stress response and apoptosis, indicating its role in managing cellular stress and programmed cell death. These genes may regulate the balance between survival and apoptosis, particularly in contexts like cancer and retinal degeneration, by influencing key signaling pathways such as XBP1 and IRE1.

## Mechanisms of ER Stress Response in Cellular Apoptosis¶

Key information

Key genes: BIRC7, BIRC3, CYCS, CASP9, XIAP

Odds Ratio: 33.19

FDR: 0.000

Combined Score: 440.38

The gene set is significantly associated with pathways involved in the endoplasmic reticulum (ER) stress response and apoptosis, highlighting its role in cellular stress management and programmed cell death. These genes may contribute to the regulation of pro-survival and pro-apoptotic signals, potentially influencing cell fate decisions in conditions such as cancer and retinal degeneration.

StringDB references

- Adaptation to ER stress is mediated by differential stabilities of pro-survival and pro-apoptotic mRNAs and proteins. (PMID:17090218)
- Evasion of apoptosis as a cellular stress response in cancer. (PMID:20182539)
- ER stress and apoptosis: a new mechanism for retinal cell death. (PMID:22216020)
- ER-stress-induced transcriptional regulation increases protein synthesis leading to cell death. (PMID:23624402)
- The Inhibitor of Apoptosis (IAPs) in Adaptive Response to Cellular Stress. (PMID:24710527)

Ontology genesets

- Intrinsic Apoptotic Signaling Pathway In Response To Endoplasmic Reticulum Stress (GO:0070059)
- Regulation Of Endoplasmic Reticulum Stress-Induced Intrinsic Apoptotic Signaling Pathway (GO:1902235)
- Response To Endoplasmic Reticulum Stress (GO:0034976)
- Positive Regulation Of Response To Endoplasmic Reticulum Stress (GO:1905898)
- Positive Regulation Of Programmed Cell Death (GO:0043068)

Gene overlap matrix

Gene overlap matrix

## Mechanisms of ER Stress-Induced Apoptosis via XBP1 Signaling¶

Key information

Key genes: XBP1, HSPA5, ATF6, DNAJB9, EDEM1

Odds Ratio: 54.08

FDR: 0.000

Combined Score: 982.84

The gene set is significantly associated with pathways involved in ER stress-induced apoptosis, particularly highlighting the role of XBP1 signaling in cellular responses to stress. These genes may play a crucial role in modulating cell survival and death mechanisms during ER stress, potentially influencing pathways such as IRE1-JNK signaling and the regulation of pro-survival versus pro-apoptotic factors.

StringDB references

- Adaptation to ER stress is mediated by differential stabilities of pro-survival and pro-apoptotic mRNAs and proteins. (PMID:17090218)
- HSP72 protects cells from ER stress-induced apoptosis via enhancement of IRE1alpha-XBP1 signaling through a physical interaction. (PMID:20625543)
- ER-stress-induced transcriptional regulation increases protein synthesis leading to cell death. (PMID:23624402)
- Cab45S inhibits the ER stress-induced IRE1-JNK pathway and apoptosis via GRP78BiP. (PMID:24810055)
- XBP1 mitigates aminoglycoside-induced endoplasmic reticulum stress and neuronal cell death. (PMID:25973683)

Ontology genesets

- Intrinsic Apoptotic Signaling Pathway In Response To Endoplasmic Reticulum Stress (GO:0070059)
- Regulation Of Endoplasmic Reticulum Stress-Induced Intrinsic Apoptotic Signaling Pathway (GO:1902235)
- Negative Regulation Of Intrinsic Apoptotic Signaling Pathway (GO:2001243)
- Positive Regulation Of Response To Endoplasmic Reticulum Stress (GO:1905898)
- Response To Endoplasmic Reticulum Stress (GO:0034976)

Gene overlap matrix

Gene overlap matrix

## Mechanisms of ER Stress Response and Apoptosis Regulation¶

Key information

Key genes: BCL2, XBP1, ATF4, ATF6, BCL2L11

Odds Ratio: 35.60

FDR: 0.000

Combined Score: 572.77

The gene set is significantly associated with pathways involved in the ER stress response and apoptosis regulation, highlighting its role in cellular stress adaptation and cell death mechanisms. These genes may contribute to the balance between survival and apoptosis during ER stress, potentially influencing retinal cell survival and the broader implications of cellular stress responses.

StringDB references

- Adaptation to ER stress is mediated by differential stabilities of pro-survival and pro-apoptotic mRNAs and proteins. (PMID:17090218)
- ER stress and apoptosis: a new mechanism for retinal cell death. (PMID:22216020)
- ER-stress-induced transcriptional regulation increases protein synthesis leading to cell death. (PMID:23624402)
- The Inhibitor of Apoptosis (IAPs) in Adaptive Response to Cellular Stress. (PMID:24710527)
- ER Stress-Sensor Proteins and ER-Mitochondrial Crosstalk-Signaling Beyond (ER) Stress Response. (PMID:33525374)

Ontology genesets

- Intrinsic Apoptotic Signaling Pathway In Response To Endoplasmic Reticulum Stress (GO:0070059)
- Regulation Of Endoplasmic Reticulum Stress-Induced Intrinsic Apoptotic Signaling Pathway (GO:1902235)
- Positive Regulation Of Response To Endoplasmic Reticulum Stress (GO:1905898)
- Response To Endoplasmic Reticulum Stress (GO:0034976)
- Positive Regulation Of Programmed Cell Death (GO:0043068)

Gene overlap matrix

Gene overlap matrix

## Mechanisms of ER Stress-Induced Apoptosis via XBP1 and IRE1¶

Key information

Key genes: XBP1, HSPA5, ATF6, DNAJB9, EDEM1

Odds Ratio: 45.50

FDR: 0.000

Combined Score: 664.20

The gene set is significantly associated with pathways involved in ER stress-induced apoptosis, particularly highlighting the roles of XBP1 and IRE1 in cellular responses to stress. These genes may function by modulating apoptotic signaling pathways, such as enhancing IRE1alpha-XBP1 signaling and interacting with Bcl-2 homologs, ultimately influencing cell survival during ER stress.

StringDB references

- HSP72 protects cells from ER stress-induced apoptosis via enhancement of IRE1alpha-XBP1 signaling through a physical interaction. (PMID:20625543)
- ER-stress-induced transcriptional regulation increases protein synthesis leading to cell death. (PMID:23624402)
- Cab45S inhibits the ER stress-induced IRE1-JNK pathway and apoptosis via GRP78BiP. (PMID:24810055)
- XBP1 mitigates aminoglycoside-induced endoplasmic reticulum stress and neuronal cell death. (PMID:25973683)
- IRBIT controls apoptosis by interacting with the Bcl-2 homolog, Bcl2l10, and by promoting ER-mitochondria contact. (PMID:27995898)

Ontology genesets

- Intrinsic Apoptotic Signaling Pathway In Response To Endoplasmic Reticulum Stress (GO:0070059)
- Regulation Of Endoplasmic Reticulum Stress-Induced Intrinsic Apoptotic Signaling Pathway (GO:1902235)
- Regulation Of IRE1-mediated Unfolded Protein Response (GO:1903894)
- IRE1-mediated Unfolded Protein Response (GO:0036498)
- Positive Regulation Of Response To Endoplasmic Reticulum Stress (GO:1905898)

Gene overlap matrix

Gene overlap matrix

## Mechanisms of ER Stress-Induced Apoptosis in Cancer¶

Key information

Key genes: XBP1, ATF4, ATF6, BCL2L11, BCL2

Odds Ratio: 25.62

FDR: 0.001

Combined Score: 253.18

The gene set is significantly associated with pathways related to endoplasmic reticulum (ER) stress and apoptosis, indicating its involvement in critical cellular processes that regulate cell survival and death in cancer. These genes may play a role in modulating the balance between pro-survival and pro-apoptotic signals during ER stress, potentially influencing tumor progression and response to therapy.

StringDB references

- Adaptation to ER stress is mediated by differential stabilities of pro-survival and pro-apoptotic mRNAs and proteins. (PMID:17090218)
- Evasion of apoptosis as a cellular stress response in cancer. (PMID:20182539)
- ER stress and apoptosis: a new mechanism for retinal cell death. (PMID:22216020)
- ER-stress-induced transcriptional regulation increases protein synthesis leading to cell death. (PMID:23624402)
- Endoplasmic reticulum stress, cell death and tumor: Association between endoplasmic reticulum stress and the apoptosis pathway in tumors (Review). (PMID:33469681)

Ontology genesets

- Intrinsic Apoptotic Signaling Pathway In Response To Endoplasmic Reticulum Stress (GO:0070059)
- Regulation Of Endoplasmic Reticulum Stress-Induced Intrinsic Apoptotic Signaling Pathway (GO:1902235)
- Response To Endoplasmic Reticulum Stress (GO:0034976)
- Positive Regulation Of Response To Endoplasmic Reticulum Stress (GO:1905898)
- Intrinsic Apoptotic Signaling Pathway In Response To DNA Damage (GO:0008630)

Gene overlap matrix

Gene overlap matrix


← Theme 1 - Regulation of Biosynthetic Processes in Cancer

Theme 3 - Ethanol Effects on Ceramide Metabolism  →

Page contents

- Theme 2 - ER Stress and Apoptosis Mechanisms
  - Mechanisms of ER Stress Response in Cellular Apoptosis
  - Mechanisms of ER Stress-Induced Apoptosis via XBP1 Signaling
  - Mechanisms of ER Stress Response and Apoptosis Regulation
  - Mechanisms of ER Stress-Induced Apoptosis via XBP1 and IRE1
  - Mechanisms of ER Stress-Induced Apoptosis in Cancer

- Contact
- Developers
