# Supplementary material for: GeneInsight: Condensing gene set knowledge via language models
Source: PLoS Comput Biol. 2026 Aug 5;22(8):e1014500. doi: 10.1371/journal.pcbi.1014500 (PMC13440848; doi:10.1371/journal.pcbi.1014500)
Supplement: Supplementary file 6 [file pcbi.1014500.s004.zip › S4 Data/sphinx_builds.zip!/sphinx_builds/html_build_geneset3/build/html/cluster_5.html]

Theme 6 - Transcription Factor Activity Regulation in Elongation — GeneInsight documentation


Skip to content

GeneInsight


menu

Major themes:

- Theme 1 - Regulation of Biosynthetic Processes in Cancer
- Theme 2 - ER Stress and Apoptosis Mechanisms
- Theme 3 - Ethanol Effects on Ceramide Metabolism
- Theme 4 - Membrane-Bounded Organelle Functions and Assembly
- Theme 5 - Unfolded Protein Response in Disease
- Theme 6 - Transcription Factor Activity Regulation in Elongation
- Theme 7 - FGFR2 Mutation and Osteocyte Function in Osteoporosis
- Theme 8 - CD4+ T Cell Activation Mechanisms
- Theme 9 - Regulation of Transcription Factor Activity
- Theme 10 - Epithelial Cell Differentiation and Proliferation

Metadata:

- Embedding map
- Summary statistics
- Data Files
- Downloads


Toggle menu contents

1. Docs
2. Theme 6 - Transcription Factor Activity Regulation in Elongation

JavaScript is required to toggle light/dark mode..


 Light mode
 Dark mode


Edit on GitHub


View source

---

# Theme 6 - Transcription Factor Activity Regulation in Elongation¶

The gene set is significantly associated with the regulation of DNA-binding transcription factor activity, particularly in the context of transcription elongation. This suggests that the genes may play a crucial role in enhancing the transcriptional activity of specific factors, potentially influencing gene expression patterns during cellular processes such as development or response to environmental stimuli.

## Regulation of DNA-binding Transcription Factor Activity in Elongation¶

Key information

Key genes: NFKBIA, NFKBIE, NFKBIB, CHUK, RELA

Odds Ratio: 35.09

FDR: 0.000

Combined Score: 473.91

The gene set is significantly associated with the regulation of DNA-binding transcription factor activity, particularly in the context of transcription elongation. This suggests that the genes may play a crucial role in enhancing the transcriptional activity of specific factors, potentially influencing gene expression patterns during cellular processes such as development or response to environmental stimuli.

StringDB references

- Positive regulation of DNA-templated transcription, elongation (GO:0032786)
- Regulation of DNA-binding transcription factor activity (GO:0051090)

Ontology genesets

- Regulation Of DNA-templated Transcription Elongation (GO:0032784)
- Positive Regulation Of DNA-templated Transcription, Elongation (GO:0032786)
- Negative Regulation Of DNA-binding Transcription Factor Activity (GO:0043433)
- Positive Regulation Of DNA-binding Transcription Factor Activity (GO:0051091)
- Positive Regulation Of DNA-templated Transcription (GO:0045893)

Gene overlap matrix

Gene overlap matrix


← Theme 5 - Unfolded Protein Response in Disease

Theme 7 - FGFR2 Mutation and Osteocyte Function in Osteoporosis  →

Page contents

- Theme 6 - Transcription Factor Activity Regulation in Elongation
  - Regulation of DNA-binding Transcription Factor Activity in Elongation

- Contact
- Developers
