# Supplementary material for: GeneInsight: Condensing gene set knowledge via language models
Source: PLoS Comput Biol. 2026 Aug 5;22(8):e1014500. doi: 10.1371/journal.pcbi.1014500 (PMC13440848; doi:10.1371/journal.pcbi.1014500)
Supplement: Supplementary file 6 [file pcbi.1014500.s004.zip › S4 Data/sphinx_builds.zip!/sphinx_builds/html_build_geneset3/build/html/cluster_3.html]

Theme 4 - Membrane-Bounded Organelle Functions and Assembly — GeneInsight documentation


Skip to content

GeneInsight


menu

Major themes:

- Theme 1 - Regulation of Biosynthetic Processes in Cancer
- Theme 2 - ER Stress and Apoptosis Mechanisms
- Theme 3 - Ethanol Effects on Ceramide Metabolism
- Theme 4 - Membrane-Bounded Organelle Functions and Assembly
- Theme 5 - Unfolded Protein Response in Disease
- Theme 6 - Transcription Factor Activity Regulation in Elongation
- Theme 7 - FGFR2 Mutation and Osteocyte Function in Osteoporosis
- Theme 8 - CD4+ T Cell Activation Mechanisms
- Theme 9 - Regulation of Transcription Factor Activity
- Theme 10 - Epithelial Cell Differentiation and Proliferation

Metadata:

- Embedding map
- Summary statistics
- Data Files
- Downloads


Toggle menu contents

1. Docs
2. Theme 4 - Membrane-Bounded Organelle Functions and Assembly

JavaScript is required to toggle light/dark mode..


 Light mode
 Dark mode


Edit on GitHub


View source

---

# Theme 4 - Membrane-Bounded Organelle Functions and Assembly¶

The gene set is significantly associated with the assembly and function of membrane-bounded organelles, highlighting its role in crucial cellular processes related to organelle formation and maintenance. This suggests that the genes in this set may be vital for maintaining organelle structure and functionality, influencing cellular metabolism and signaling pathways.

## Membrane-Bounded Organelle Component Assembly and Function¶

Key information

Key genes: EIF2S1, ATF4, RPS23, RPL38, RPS28

Odds Ratio: 18.00

FDR: 0.000

Combined Score: 225.64

The gene set is significantly associated with the assembly and function of membrane-bounded organelles, indicating its involvement in crucial cellular processes related to organelle formation and maintenance. These genes may play essential roles in the structural integrity and functionality of organelles, potentially influencing cellular metabolism and signaling pathways.

StringDB references

- Non-membrane-bounded organelle assembly (GO:0140694)
- Membrane-bounded organelle (GOCC:0043227)

Ontology genesets

- Intracellular Membrane-Bounded Organelle (GO:0043231)
- Cytoplasmic Vesicle Lumen (GO:0060205)
- Positive Regulation Of Endoplasmic Reticulum Unfolded Protein Response (GO:1900103)
- Mitochondrial Respiratory Chain Complex III (GO:0005750)
- Ribosome (GO:0005840)

Gene overlap matrix

Gene overlap matrix

## Membrane-Bounded Organelles and Their Intracellular Functions¶

Key information

Key genes: CYCS, CANX, HSPA5, HSPA9, ITPR1

Odds Ratio: 22.16

FDR: 0.000

Combined Score: 394.93

The gene set is significantly associated with pathways related to membrane-bounded organelles, indicating its involvement in critical intracellular functions such as transport, signaling, and metabolic processes. This suggests that the genes in this set may play essential roles in maintaining cellular organization and function by regulating the dynamics and interactions of these organelles.

StringDB references

- Intracellular membrane-bounded organelle (GOCC:0043231)

Ontology genesets

- Intracellular Organelle Lumen (GO:0070013)
- Endoplasmic Reticulum Membrane (GO:0005789)
- Lumenal Side Of Endoplasmic Reticulum Membrane (GO:0098553)
- Endocytic Vesicle Membrane (GO:0030666)
- Phagocytic Vesicle Membrane (GO:0030670)

Gene overlap matrix

Gene overlap matrix

## Membrane-Bounded Organelle Components and Their Functions¶

Key information

Key genes: XBP1, EIF2S1, ATF4, MAPK14, CEBPB

Odds Ratio: 21.66

FDR: 0.000

Combined Score: 382.58

The gene set is significantly associated with pathways related to membrane-bounded organelles, indicating its involvement in essential cellular processes such as compartmentalization and intracellular transport. These genes may play critical roles in maintaining organelle structure and function, potentially influencing processes like protein sorting and lipid metabolism within the cell.

StringDB references

- Membrane-bounded organelle (GOCC:0043227)
- Intracellular membrane-bounded organelle (GOCC:0043231)

Ontology genesets

- Intracellular Membrane-Bounded Organelle (GO:0043231)
- Intracellular Organelle Lumen (GO:0070013)
- Endoplasmic Reticulum Membrane (GO:0005789)
- Cytoplasmic Vesicle Lumen (GO:0060205)
- Endoplasmic Reticulum To Cytosol Transport (GO:1903513)

Gene overlap matrix

Gene overlap matrix


← Theme 3 - Ethanol Effects on Ceramide Metabolism

Theme 5 - Unfolded Protein Response in Disease  →

Page contents

- Theme 4 - Membrane-Bounded Organelle Functions and Assembly
  - Membrane-Bounded Organelle Component Assembly and Function
  - Membrane-Bounded Organelles and Their Intracellular Functions
  - Membrane-Bounded Organelle Components and Their Functions

- Contact
- Developers
