# Supplementary material for: GeneInsight: Condensing gene set knowledge via language models
Source: PLoS Comput Biol. 2026 Aug 5;22(8):e1014500. doi: 10.1371/journal.pcbi.1014500 (PMC13440848; doi:10.1371/journal.pcbi.1014500)
Supplement: Supplementary file 6 [file pcbi.1014500.s004.zip › S4 Data/sphinx_builds.zip!/sphinx_builds/html_build_geneset3/build/html/cluster_6.html]

Theme 7 - FGFR2 Mutation and Osteocyte Function in Osteoporosis — GeneInsight documentation


Skip to content

GeneInsight


menu

Major themes:

- Theme 1 - Regulation of Biosynthetic Processes in Cancer
- Theme 2 - ER Stress and Apoptosis Mechanisms
- Theme 3 - Ethanol Effects on Ceramide Metabolism
- Theme 4 - Membrane-Bounded Organelle Functions and Assembly
- Theme 5 - Unfolded Protein Response in Disease
- Theme 6 - Transcription Factor Activity Regulation in Elongation
- Theme 7 - FGFR2 Mutation and Osteocyte Function in Osteoporosis
- Theme 8 - CD4+ T Cell Activation Mechanisms
- Theme 9 - Regulation of Transcription Factor Activity
- Theme 10 - Epithelial Cell Differentiation and Proliferation

Metadata:

- Embedding map
- Summary statistics
- Data Files
- Downloads


Toggle menu contents

1. Docs
2. Theme 7 - FGFR2 Mutation and Osteocyte Function in Osteoporosis

JavaScript is required to toggle light/dark mode..


 Light mode
 Dark mode


Edit on GitHub


View source

---

# Theme 7 - FGFR2 Mutation and Osteocyte Function in Osteoporosis¶

The gene set is significantly associated with pathways related to bone metabolism, particularly highlighting the roles of FGFR2 mutations in osteocyte function and their link to osteoporosis. These genes may influence osteoclastogenesis and osteoblast activity through pathways involving RANKL signaling and NF-kappaB, suggesting that they play a critical role in maintaining bone health and regulating bone density.

## FGFR2 Mutation Impact on Osteocyte Function and Osteoporosis¶

Key information

Key genes: NFKBIA, RELB, NFKB1, NFKBIE, REL

Odds Ratio: 23.24

FDR: 0.001

Combined Score: 221.65

The gene set is significantly associated with pathways related to bone metabolism, particularly highlighting the roles of FGFR2 mutations in osteocyte function and their link to osteoporosis. These genes may influence osteoclastogenesis and osteoblast activity through pathways involving RANKL signaling and NF-kappaB, suggesting that they play a critical role in maintaining bone health and regulating bone density.

StringDB references

- The osteogenic transcription factor Runx2 regulates components of the fibroblast growth factorproteoglycan signaling axis in osteoblasts. (PMID:19259985)
- IGFBP7 acts as a negative regulator of RANKL-induced osteoclastogenesis and oestrogen deficiency-induced bone loss. (PMID:31889368)
- Critical Roles of NF-kappaB Signaling Molecules in Bone Metabolism Revealed by Genetic Mutations in Osteopetrosis. (PMID:35887342)
- An FGFR2 mutation as the potential cause of a new phenotype including early-onset osteoporosis and bone fractures: a case report. (PMID:38098042)

Ontology genesets

- Regulation Of Bone Resorption (GO:0045124)
- Positive Regulation Of Bone Resorption (GO:0045780)
- Recurrent fractures (HP:0002757)
- Positive Regulation Of NF-kappaB Transcription Factor Activity (GO:0051092)
- Regulation Of I-kappaB kinase/NF-kappaB Signaling (GO:0043122)

Gene overlap matrix

Gene overlap matrix


← Theme 6 - Transcription Factor Activity Regulation in Elongation

Theme 8 - CD4+ T Cell Activation Mechanisms  →

Page contents

- Theme 7 - FGFR2 Mutation and Osteocyte Function in Osteoporosis
  - FGFR2 Mutation Impact on Osteocyte Function and Osteoporosis

- Contact
- Developers
