# Supplementary material for: GeneInsight: Condensing gene set knowledge via language models
Source: PLoS Comput Biol. 2026 Aug 5;22(8):e1014500. doi: 10.1371/journal.pcbi.1014500 (PMC13440848; doi:10.1371/journal.pcbi.1014500)
Supplement: Supplementary file 6 [file pcbi.1014500.s004.zip › S4 Data/sphinx_builds.zip!/sphinx_builds/html_build_geneset3/build/html/cluster_0.html]

Theme 1 - Regulation of Biosynthetic Processes in Cancer — GeneInsight documentation


Skip to content

GeneInsight


menu

Major themes:

- Theme 1 - Regulation of Biosynthetic Processes in Cancer
- Theme 2 - ER Stress and Apoptosis Mechanisms
- Theme 3 - Ethanol Effects on Ceramide Metabolism
- Theme 4 - Membrane-Bounded Organelle Functions and Assembly
- Theme 5 - Unfolded Protein Response in Disease
- Theme 6 - Transcription Factor Activity Regulation in Elongation
- Theme 7 - FGFR2 Mutation and Osteocyte Function in Osteoporosis
- Theme 8 - CD4+ T Cell Activation Mechanisms
- Theme 9 - Regulation of Transcription Factor Activity
- Theme 10 - Epithelial Cell Differentiation and Proliferation

Metadata:

- Embedding map
- Summary statistics
- Data Files
- Downloads


Toggle menu contents

1. Docs
2. Theme 1 - Regulation of Biosynthetic Processes in Cancer

JavaScript is required to toggle light/dark mode..


 Light mode
 Dark mode


Edit on GitHub


View source

---

# Theme 1 - Regulation of Biosynthetic Processes in Cancer¶

The gene set is significantly associated with various pathways related to the regulation of macromolecule and organic compound biosynthetic processes, indicating its crucial role in synthesizing essential biological compounds and influencing metabolic pathways. These genes may enhance cellular growth and development, particularly in the context of glucose metabolism reprogramming in breast cancer, suggesting that they could be targeted for therapeutic strategies to combat cancer therapy resistance.

## Regulation of macromolecule biosynthetic processes involving organic compounds¶

Key information

Key genes: XBP1, ATF6, DDIT3, CCND1, MAPK8IP1

Odds Ratio: 17.28

FDR: 0.000

Combined Score: 180.07

The gene set is significantly associated with the regulation of macromolecule biosynthetic processes, indicating its involvement in the synthesis and regulation of essential biological compounds. This suggests that the genes within this set may play a crucial role in enhancing the production of macromolecules, potentially influencing metabolic pathways and cellular growth.

StringDB references

- Regulation of macromolecule biosynthetic process (GO:0010556)
- Positive regulation of macromolecule biosynthetic process (GO:0010557)

Ontology genesets

- Regulation Of Lipid Biosynthetic Process (GO:0046890)
- Positive Regulation Of Cellular Biosynthetic Process (GO:0031328)
- Positive Regulation Of Biosynthetic Process (GO:0009891)
- Positive Regulation Of Lipid Biosynthetic Process (GO:0046889)
- Positive Regulation Of Macromolecule Metabolic Process (GO:0010604)

Gene overlap matrix

Gene overlap matrix

## Glucose Metabolism Reprogramming in Breast Cancer Therapy¶

Key information

Key genes: PFKP, PFKL, PFKM, ALDOC, ALDOA

Odds Ratio: 39.98

FDR: 0.000

Combined Score: 456.37

The gene set is significantly associated with pathways related to glucose metabolism reprogramming in breast cancer, indicating a crucial role in the altered metabolic processes that support tumor growth and survival. These genes may contribute to the mechanisms of cancer therapy resistance by enhancing the glycolytic pathway, particularly in triple-negative breast cancer, suggesting that targeting glucose metabolism could be a potential therapeutic strategy.

StringDB references

- Reprogramming glucose metabolism in cancer: can it be exploited for cancer therapy? (PMID:27634447)
- The Role of Reprogrammed Glucose Metabolism in Cancer. (PMID:36984785)
- Glucose metabolism in triple-negative breast cancer cells (WP5211)

Ontology genesets

- Glucose Metabolic Process (GO:0006006)
- Fructose Metabolic Process (GO:0006000)
- Fructose 6-Phosphate Metabolic Process (GO:0006002)
- Abnormality of glycolysis (HP:0004366)
- Pyruvate Metabolic Process (GO:0006090)

Gene overlap matrix

Gene overlap matrix

## Regulation of organic compound biosynthetic processes in cells¶

Key information

Key genes: PPARGC1A, MAPK1, EIF4E2, RPTOR, EIF4EBP1

Odds Ratio: 15.93

FDR: 0.000

Combined Score: 160.30

The gene set is significantly associated with the regulation of organic compound biosynthetic processes, indicating its involvement in essential cellular functions related to the synthesis of various biomolecules. These genes may play critical roles in modulating metabolic pathways that govern the production of organic compounds, potentially influencing cellular growth and development.

StringDB references

- Regulation of cellular biosynthetic process (GO:0031326)

Ontology genesets

- Positive Regulation Of Cellular Biosynthetic Process (GO:0031328)
- Positive Regulation Of Macromolecule Biosynthetic Process (GO:0010557)
- Positive Regulation Of Biosynthetic Process (GO:0009891)
- Negative Regulation Of Biosynthetic Process (GO:0009890)
- Regulation Of Lipid Biosynthetic Process (GO:0046890)

Gene overlap matrix

Gene overlap matrix

## Regulation of macromolecule biosynthetic and metabolic processes¶

Key information

Key genes: CCND1, MAPK8IP1, NEUROG3, RCOR1, ASCL1

Odds Ratio: 17.57

FDR: 0.000

Combined Score: 217.92

The gene set is significantly associated with the regulation of macromolecule biosynthetic and metabolic processes, indicating its involvement in essential biological functions that govern the synthesis and breakdown of large biomolecules. This suggests that the genes in this set may play critical roles in cellular growth and metabolism, potentially influencing pathways related to protein synthesis and energy production.

StringDB references

- Regulation of macromolecule biosynthetic process (GO:0010556)
- Regulation of macromolecule metabolic process (GO:0060255)

Ontology genesets

- Regulation Of Protein Metabolic Process (GO:0051246)
- Regulation Of Lipid Biosynthetic Process (GO:0046890)
- Positive Regulation Of Macromolecule Metabolic Process (GO:0010604)
- Positive Regulation Of Lipid Biosynthetic Process (GO:0046889)
- Positive Regulation Of Lipid Metabolic Process (GO:0045834)

Gene overlap matrix

Gene overlap matrix


← GeneInsight Topic Descriptions

Theme 2 - ER Stress and Apoptosis Mechanisms  →

Page contents

- Theme 1 - Regulation of Biosynthetic Processes in Cancer
  - Regulation of macromolecule biosynthetic processes involving organic compounds
  - Glucose Metabolism Reprogramming in Breast Cancer Therapy
  - Regulation of organic compound biosynthetic processes in cells
  - Regulation of macromolecule biosynthetic and metabolic processes

- Contact
- Developers
