# Supplementary material for: GeneInsight: Condensing gene set knowledge via language models
Source: PLoS Comput Biol. 2026 Aug 5;22(8):e1014500. doi: 10.1371/journal.pcbi.1014500 (PMC13440848; doi:10.1371/journal.pcbi.1014500)
Supplement: Supplementary file 6 [file pcbi.1014500.s004.zip › S4 Data/sphinx_builds.zip!/sphinx_builds/html_build_geneset3/build/html/cluster_8.html]

Theme 9 - Regulation of Transcription Factor Activity — GeneInsight documentation


Skip to content

GeneInsight


menu

Major themes:

- Theme 1 - Regulation of Biosynthetic Processes in Cancer
- Theme 2 - ER Stress and Apoptosis Mechanisms
- Theme 3 - Ethanol Effects on Ceramide Metabolism
- Theme 4 - Membrane-Bounded Organelle Functions and Assembly
- Theme 5 - Unfolded Protein Response in Disease
- Theme 6 - Transcription Factor Activity Regulation in Elongation
- Theme 7 - FGFR2 Mutation and Osteocyte Function in Osteoporosis
- Theme 8 - CD4+ T Cell Activation Mechanisms
- Theme 9 - Regulation of Transcription Factor Activity
- Theme 10 - Epithelial Cell Differentiation and Proliferation

Metadata:

- Embedding map
- Summary statistics
- Data Files
- Downloads


Toggle menu contents

1. Docs
2. Theme 9 - Regulation of Transcription Factor Activity

JavaScript is required to toggle light/dark mode..


 Light mode
 Dark mode


Edit on GitHub


View source

---

# Theme 9 - Regulation of Transcription Factor Activity¶

The gene set is significantly associated with the regulation of DNA-binding transcription factor activity, indicating its involvement in key biological processes that control gene expression. This suggests that the genes in this set may play critical roles in modulating repressor activity, potentially influencing cellular responses to various stimuli and maintaining cellular homeostasis.

## Regulation of DNA-binding Transcription Factor Activity in Repressors¶

Key information

Key genes: CD40, TRAF1, TRAF3, NFKBIA, NFKBIE

Odds Ratio: 28.55

FDR: 0.000

Combined Score: 292.92

The gene set is significantly associated with the regulation of DNA-binding transcription factor activity, indicating its involvement in key biological processes that control gene expression. This suggests that the genes in this set may play critical roles in modulating repressor activity, potentially influencing cellular responses to various stimuli and maintaining cellular homeostasis.

StringDB references

- Regulation of DNA-binding transcription factor activity (GO:0051090)

Ontology genesets

- Negative Regulation Of DNA-binding Transcription Factor Activity (GO:0043433)
- Positive Regulation Of DNA-binding Transcription Factor Activity (GO:0051091)
- Regulation Of DNA-templated Transcription (GO:0006355)
- Positive Regulation Of DNA-templated Transcription (GO:0045893)
- Positive Regulation Of Nucleic Acid-Templated Transcription (GO:1903508)

Gene overlap matrix

Gene overlap matrix

## Regulation of DNA-binding Transcription Factor Activity by Repressors¶

Key information

Key genes: NFKBIA, NFKBIE, NFKBIB, CHUK, RELA

Odds Ratio: 32.24

FDR: 0.000

Combined Score: 344.30

The gene set is significantly associated with the regulation of DNA-binding transcription factor activity, indicating its involvement in critical biological processes that control gene expression. This suggests that the genes within this set may act as repressors, modulating transcriptional activity and influencing cellular responses to various stimuli.

StringDB references

- Regulation of DNA-binding transcription factor activity (GO:0051090)

Ontology genesets

- Negative Regulation Of DNA-binding Transcription Factor Activity (GO:0043433)
- Positive Regulation Of DNA-binding Transcription Factor Activity (GO:0051091)
- Regulation Of DNA-templated Transcription (GO:0006355)
- Positive Regulation Of DNA-templated Transcription (GO:0045893)
- Positive Regulation Of Nucleic Acid-Templated Transcription (GO:1903508)

Gene overlap matrix

Gene overlap matrix


← Theme 8 - CD4+ T Cell Activation Mechanisms

Theme 10 - Epithelial Cell Differentiation and Proliferation  →

Page contents

- Theme 9 - Regulation of Transcription Factor Activity
  - Regulation of DNA-binding Transcription Factor Activity in Repressors
  - Regulation of DNA-binding Transcription Factor Activity by Repressors

- Contact
- Developers
