# Supplementary material for: GeneInsight: Condensing gene set knowledge via language models
Source: PLoS Comput Biol. 2026 Aug 5;22(8):e1014500. doi: 10.1371/journal.pcbi.1014500 (PMC13440848; doi:10.1371/journal.pcbi.1014500)
Supplement: Supplementary file 6 [file pcbi.1014500.s004.zip › S4 Data/sphinx_builds.zip!/sphinx_builds/html_build_geneset3/build/html/cluster_4.html]

Theme 5 - Unfolded Protein Response in Disease — GeneInsight documentation


Skip to content

GeneInsight


menu

Major themes:

- Theme 1 - Regulation of Biosynthetic Processes in Cancer
- Theme 2 - ER Stress and Apoptosis Mechanisms
- Theme 3 - Ethanol Effects on Ceramide Metabolism
- Theme 4 - Membrane-Bounded Organelle Functions and Assembly
- Theme 5 - Unfolded Protein Response in Disease
- Theme 6 - Transcription Factor Activity Regulation in Elongation
- Theme 7 - FGFR2 Mutation and Osteocyte Function in Osteoporosis
- Theme 8 - CD4+ T Cell Activation Mechanisms
- Theme 9 - Regulation of Transcription Factor Activity
- Theme 10 - Epithelial Cell Differentiation and Proliferation

Metadata:

- Embedding map
- Summary statistics
- Data Files
- Downloads


Toggle menu contents

1. Docs
2. Theme 5 - Unfolded Protein Response in Disease

JavaScript is required to toggle light/dark mode..


 Light mode
 Dark mode


Edit on GitHub


View source

---

# Theme 5 - Unfolded Protein Response in Disease¶

The gene set is significantly associated with the mechanisms of the unfolded protein response (UPR) in cancer and viral infections, indicating its involvement in critical biological processes related to cellular stress and protein misfolding. These genes may play a crucial role in modulating cancer cell survival and viral replication by regulating UPR pathways, suggesting their importance in maintaining cellular homeostasis and potential therapeutic targets in cancer and viral diseases.

## Mechanisms of Unfolded Protein Response in Cancer¶

Key information

Key genes: XBP1, ATF6, DDIT3, HSPA5, ERN1

Odds Ratio: 42.36

FDR: 0.000

Combined Score: 605.74

The gene set is significantly associated with the mechanisms of the unfolded protein response (UPR) in cancer, indicating its involvement in critical biological processes related to cellular stress and protein misfolding. These genes may play a crucial role in modulating cancer cell survival and proliferation by regulating the UPR pathways, which are essential for maintaining cellular homeostasis under stress conditions.

StringDB references

- Cellular response to unfolded protein (GO:0034620)
- Unfolded protein response in cancer: the physicians perspective. (PMID:21345215)
- Unfolded Protein Response and Cancer. (PMID:32309542)
- Unfolded protein response in colorectal cancer. (PMID:33514437)

Ontology genesets

- Response To Unfolded Protein (GO:0006986)
- IRE1-mediated Unfolded Protein Response (GO:0036498)
- Regulation Of IRE1-mediated Unfolded Protein Response (GO:1903894)
- Regulation Of PERK-mediated Unfolded Protein Response (GO:1903897)
- PERK-mediated Unfolded Protein Response (GO:0036499)

Gene overlap matrix

Gene overlap matrix

## Unfolded Protein Response Mechanisms in Coronavirus Infections¶

Key information

Key genes: XBP1, DNAJB9, HSPA5, EDEM1, ATF6

Odds Ratio: 76.92

FDR: 0.000

Combined Score: 1040.71

The gene set is significantly associated with the unfolded protein response (UPR) mechanisms, particularly in the context of coronavirus and herpesvirus infections, suggesting a critical role in managing cellular stress during viral replication. These genes may be involved in modulating the UPR pathways to enhance viral survival or replication, indicating potential targets for therapeutic intervention in viral diseases.

StringDB references

- A human coronavirus OC43 variant harboring persistence-associated mutations in the S glycoprotein differentially induces the unfolded protein response in human neurons as compared to wild-type virus. (PMID:19846189)
- Herpesviruses and the Unfolded Protein Response. (PMID:31877732)
- The Unfolded Protein Response and Autophagy on the Crossroads of Coronaviruses Infections. (PMID:33996638)
- The Role of Unfolded Protein Response in Coronavirus Infection and Its Implications for Drug Design. (PMID:35003039)

Ontology genesets

- Positive Regulation Of Endoplasmic Reticulum Unfolded Protein Response (GO:1900103)
- Regulation Of Endoplasmic Reticulum Unfolded Protein Response (GO:1900101)
- IRE1-mediated Unfolded Protein Response (GO:0036498)
- Negative Regulation Of Endoplasmic Reticulum Unfolded Protein Response (GO:1900102)
- Regulation Of IRE1-mediated Unfolded Protein Response (GO:1903894)

Gene overlap matrix

Gene overlap matrix

## Differential Unfolded Protein Response in Viral Infections¶

Key information

Key genes: XBP1, DNAJB9, HSPA5, EDEM1, ATF6

Odds Ratio: 53.42

FDR: 0.000

Combined Score: 814.99

The gene set is significantly associated with the Unfolded Protein Response (UPR) pathways, which play a crucial role in cellular stress responses during viral infections. These genes may be involved in modulating the UPR to enhance viral replication or evade host immune responses, particularly in the context of coronavirus and herpesvirus infections.

StringDB references

- Herpesviruses and the Unfolded Protein Response. (PMID:31877732)
- The endoplasmic reticulum unfolded protein response - homeostasis, cell death and evolution in virus infections. (PMID:33765123)
- The Unfolded Protein Response and Autophagy on the Crossroads of Coronaviruses Infections. (PMID:33996638)
- The Role of Unfolded Protein Response in Coronavirus Infection and Its Implications for Drug Design. (PMID:35003039)

Ontology genesets

- Positive Regulation Of Endoplasmic Reticulum Unfolded Protein Response (GO:1900103)
- Regulation Of Endoplasmic Reticulum Unfolded Protein Response (GO:1900101)
- Negative Regulation Of Endoplasmic Reticulum Unfolded Protein Response (GO:1900102)
- IRE1-mediated Unfolded Protein Response (GO:0036498)
- PERK-mediated Unfolded Protein Response (GO:0036499)

Gene overlap matrix

Gene overlap matrix


← Theme 4 - Membrane-Bounded Organelle Functions and Assembly

Theme 6 - Transcription Factor Activity Regulation in Elongation  →

Page contents

- Theme 5 - Unfolded Protein Response in Disease
  - Mechanisms of Unfolded Protein Response in Cancer
  - Unfolded Protein Response Mechanisms in Coronavirus Infections
  - Differential Unfolded Protein Response in Viral Infections

- Contact
- Developers
